# Design, Synthesis and Biological Evaluation of Quinoline-8-Sulfonamides as Inhibitors of the Tumor Cell-Specific M2 Isoform of Pyruvate Kinase: Preliminary Study

**DOI:** 10.3390/molecules28062509

**Published:** 2023-03-09

**Authors:** Krzysztof Marciniec, Zuzanna Rzepka, Elwira Chrobak, Stanisław Boryczka, Małgorzata Latocha, Dorota Wrześniok, Artur Beberok

**Affiliations:** 1 Department of Organic Chemistry, Medical University of Silesia, Jagiellońska 4, 41-200 Sosnowiec, Poland; 2 Department of Pharmaceutical Chemistry, Jagiellońska 4, 41-200 Sosnowiec, Poland; 3 Department of Molecular Biology, Jagiellońska 4, 41-200 Sosnowiec, Poland

**Keywords:** PKM2 modulators, quinolinesulfonamide, molecular docking, molecular dynamics, cell viability, PKM2 level

## Abstract

Cancer cells need to carefully regulate their metabolism to keep them growing and dividing under the influence of different nutrients and oxygen levels. Muscle isoform 2 of pyruvate kinase (PKM2) is a key glycolytic enzyme involved in the generation of ATP and is critical for cancer metabolism. PKM2 is expressed in many human tumors and is regulated by complex mechanisms that promote tumor growth and proliferation. Therefore, it is considered an attractive therapeutic target for modulating tumor metabolism. Various modulators regulate PKM2, shifting it between highly active and less active states. In the presented work, a series of 8-quinolinesulfonamide derivatives of PKM2 modulators were designed using molecular docking and molecular dynamics techniques. New compounds were synthesized using the copper-catalyzed azide-alkyne cycloaddition (CuAAC) reaction. Compound **9a** was identified in in silico studies as a potent modulator of muscle isoform 2 of pyruvate kinase. The results obtained from in vitro experiments confirmed the ability of compound **9a** to reduce the intracellular pyruvate level in A549 lung cancer cells with simultaneous impact on cancer cell viability and cell-cycle phase distribution. Moreover, compound **9a** exhibited more cytotoxicity on cancer cells than normal cells, pointing to high selectivity in the mode of action. These findings indicate that the introduction of another quinolinyl fragment to the modulator molecule may have a significant impact on pyruvate levels in cancer cells and provides further directions for future research to find novel analogs suitable for clinical applications in cancer treatment.

## 1. Introduction

The interest in quinoline derivatives as therapeutic substances is derived from research on natural compounds, i.e., alkaloids present in the bark of the cinchona tree (*Cinchona L*.), such as quinine and its right-handed stereoisomer quinidine. Quinidine has anti-arrhythmic activity, while quinine exhibits antimalarial activity and antipyretic, anti-inflammatory, and analgesic properties. Reports in the contemporary literature also describe the antiproliferative activity of quinine in relation to breast cancer cells (MCF-7) [1]. An important direction of modern research on the properties of quinoline and its derivatives is to use it in the treatment of multidrug resistance in patients with cancer. Clinical studies carried out for quinine in combination with anticancer drugs: idarubicin, cytarabine, and mitoxantron have shown the effectiveness of this combination in the treatment of acute myeloid leukemia [2]. Numerous natural and synthetic derivatives of quinoline also show anticancer activity. The following topoisomerase I inhibitors exhibit such effects: camptothecin and its semisynthetic derivatives topotecan and irinotecan [3,4], and tyrosine kinase inhibitors: bosutinib, lenvatinib, cabozanthinib [5,6,7], tipifarnib [8], and ammosamides [9].

There are many contemporary literature reports describing compounds containing a pharmacophore quinoline moiety modified by sulfonamide (sulfamoyl) fragment. Despite their often simple structure, these compounds are characterized by a wide spectrum of biological activity—anticancer, antidepressant, antiviral, or analgesic [10,11,12,13,14,15,16]. A significant group of sulfamoylquinoline derivatives showing antitumor activity are compounds, whose mechanism of action is based on the inhibition of lactate dehydrogenase A (LDHA) including 3-quinolinesulfonamide derivatives [17], while a significant group of pyruvate kinase M2 (PKM2) activators are derivatives of 6-, 7-, and 8-sulfamoylquinoline [18,19].

The biological differences between normal and cancer cells are essential in the development of strategies to selectively kill cancer cells. Aerobic glycolysis (Warburg effect) is an important hallmark of most cancers and is generally used by tumor cells to maintain malignant phenotypes, including cell proliferation, metastasis, and resistance to apoptosis [20,21]. Thus, the therapeutic targeting of glycolytic enzymes, e.g., LDHA or PKM2, represents an intriguing approach for the treatment of a broad array of cancers. Pyruvate kinase (PK) plays a crucial role in regulating the rate-limiting final step of glycolysis, catalyzing the formation of pyruvate from phosphoenolpyruvate. In mammals, there are four tissue-specific PK isoforms: L, R, M1, and M2. PKM2 is the embryonic isoform. Its expression is attenuated in adult normal tissues, while it is reactivated in tumors. Increasing evidence has shown that PKM2 is upregulated in numerous cancers, including lung, skin, liver, breast, cervical, colorectal, and gastric cancers [22,23,24]. In addition to mediating glycolytic metabolism, PKM2 has also been shown to promote cancer cell proliferation, resistance to apoptosis, and angiogenesis.

PKM2 exists in two catalytically different states and converts between high- (tetrameric, R-state) and low-activity state (dimeric, T-state). PKM2, in its low-catalytic state, is chiefly active in metabolically energetic cells, including cancer cells. PKM2 (dimer) activity can be inhibited by modulating PKM2 dimer–tetramer dynamics using either PKM2 inhibitors that bind at the ATP binding active site of PKM2 (dimer) or PKM2 activators that bind at the allosteric site of PKM2, thus activating PKM2 from the dimer formation to the tetrameric formation. This regulation of PKM2 activity can provide cancer cells with the flexibility to adapt to different microenvironments. Therefore, PKM2 agonists or inhibitors can inhibit oncogenesis by blocking PKM2 on or off [25]. Therefore, PKM2 modulators constitute a group of promising candidates for anti-cancer agents with selectivity to tumor cells, as shown by numerous preclinical studies [20,26,27].

**Figure 1:**
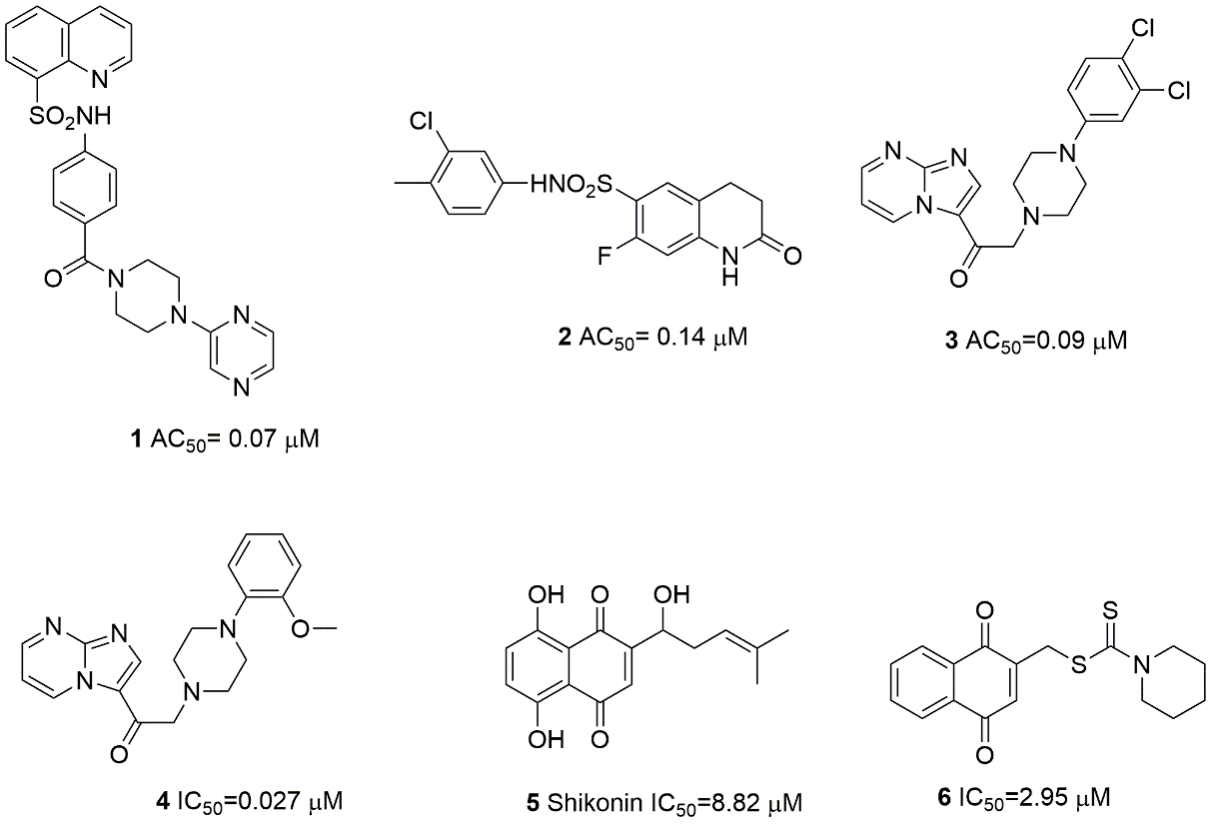
Reported pyruvate kinase M2 modulators (**1**–**3** activators, **4**–**6** inhibitors) [17,18,19].

As examples, quinoline sulfonamide derivatives belonging to the activator group [18,19] or naphthoquinone derivatives acting as inhibitors of PKM2 can be mentioned (Figure 1) [28]. A known group of PKM2 modulators are amino acids. The activators are serine, histidine, and cysteine. On the other hand, inhibitory activity is shown by tryptophan, phenylalanine, methionine, valine, and proline [29]. It should be noted that also among the newly discovered PKM2 modulators, small differences in the structure of the molecule cause a change in the way of interaction from activation to inhibition of PKM2 (compound **3** and **4** in Figure 1) [30]. A number of PKM2 modulators are 8-quinolinesulfonamide derivatives containing aryl carboxamide moieties [19]. Delightful for the research on the synthesis of 8-quinoline sulfonamide derivatives in terms of the behavior of aryl-1,2,3-triazole fragments was the fact that the 1,2,3-triazole system is a commonly used amide bioisoster in medicinal chemistry [31]. The introduction of this modification may result in better stabilization of the ligand–receptor complex, as a result of additional interactions of lone electron pairs of nitrogen atoms of the triazole fragment. Based on this hypothesis, a series of derivatives containing the 8-quinolinesulfonamide and 1,2,3-triazole moiety was designed, and then docking to PKM2 was performed. The results obtained confirmed the possibility of greater stabilization of the ligand–protein complex by the newly introduced 1,2,3-triazole group compared to the reference *N*-(4-{[4-(pyrazin-2-yl)piperazin-1-yl]carbonyl}phenyl)quinoline-8-sulfonamide **1**. Therefore, we synthesized a small series 8-quinolinesulfonamido-1,2,3-triazoles and identified compound with confirmed anticancer activity. To our knowledge, this represents the first successful attempt to click 8-sulfamoylquinoline with 1,2,3-triazoles as potent PKM2 modulators.

## 2. Results and Discussion

### 2.1. Design and In Silico Prediction of PKM-2 Modulators

**Figure 2:**
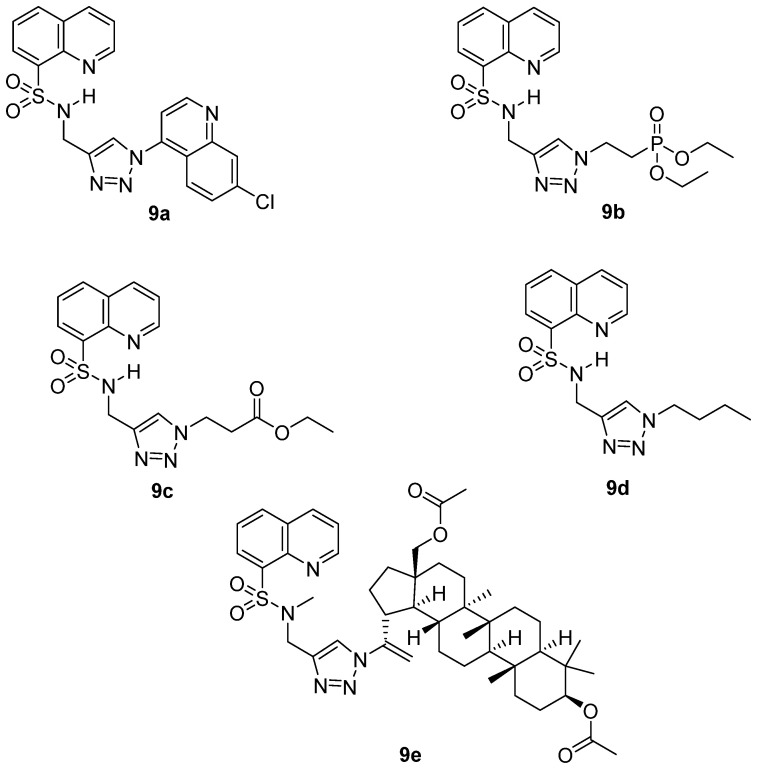
Molecular structures of novel 8-quinolinesulfonamide derivatives (**9a**–**e**) as potential PKM2 modulators.

The aim of the present study was to discover novel PKM2 modulators among novel 8-quinolinesulfonamide derivatives. From an in-house library of quinolinesulfoamoyl compounds, five derivatives were selected for synthesis (Figure 2) using GOLD software.

The single monomer of pyruvate kinase M2 is made of 531 amino acids and is composed of four domains: A, B, C, and one small N-terminal domain. The dimeric form is formed between the A domains of each monomer, whereas the tetrameric form exists due to dimerization along with the C subunit interfaces of each dimer. Activators bind to the active binding site of PKM2 (dimer), which is located between two A domains and is surrounded by key amino acid residues such as Phe26, Gly315, Tyr390, Leu394, and Leu353. On the other hand, the ATP binding site is a druggable pocket for PKM2 inhibitors. The amino acid residues corresponding to this region are Thr50, Ile51, Gly52, Pro53, His78, and Tyr83 [32].

In our in silico research, we used the PKM2 complex with an activator (PDB ID: 4G1N). At the same time, the PKM2 complex with ATP (PDB ID: 4FXF) was used to determine the potential inhibitory activity.

Pyruvate kinase M2 modulators **1**–**6** and derivatives of quinolinesulfonamide **9a**–**e** ranked by GOLD are presented in Table 1. The highest scores correspond to a strong binding affinity and the most likely ligand–protein system in the cellulo. The obtained results were presented in GOLD arbitrary units (a.u.). Later on, for the comparison and validation of the docking results, we recalculated these results using Equation (1) to ∆G [kJ/mol] [33], and then converted to kcal/mol.

y = −0.4502x – 9.4891 [kJ/mol](1)

In this case, the more negative the ∆G value of the binding reaction, the higher the binding affinity of the ligand for its specific target protein.

Comparing the docking score values obtained for 4G1N, it can be concluded that compounds **9a**–**e** show similar affinity values as the reference ligand **1** (NZT), with derivative **9a** having the lowest ∆G value (−10.72 kcal/mol). It should be noted that the remaining reference ligands **2**–**6** show a significantly lower affinity in relation to ligands **1** and **9a**–**e**. In the case of the 4FXF complex, the compound **9a** shows the lowest ∆G value and the highest binding affinity to target protein (−10.48 kcal/mol). The remaining derivatives **9b**–**e** also show a significantly higher affinity compared to the reference shikonin (**5**).

In the X-ray structure of 4G1N, the quinoline moiety of compound **1** (NZT) sits on a flat and mainly apolar surface defined by residues Phe26 and Leu394 from chain A and Phe26, Leu27, and Met 30 from chain B. One of the two oxygen atoms of the sulfonamide group accepts a hydrogen bond from the oxygen of Tyr390. Adding a methyl group to the sulfonamide nitrogen causes a steric clash with the oxygen of Leu353, and lack activity of methyl analogue of compound **1** [19]. Furthermore, oxygen of the amide moiety forms a hydrogen bond with the side chain nitrogen atom of Lys311.

**Figure 3:**
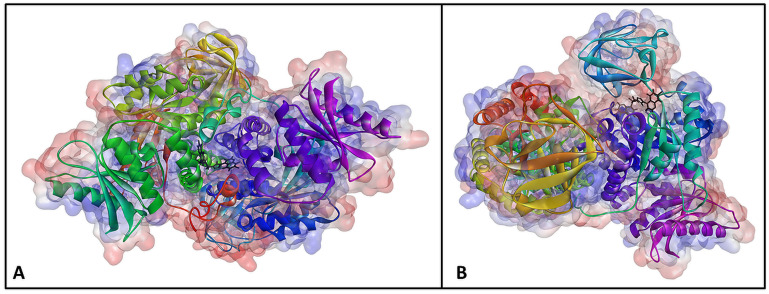
Predicted binding models of **9a** with dimeric form of PKM2 ((**A**): PDB ID 4G1N, (**B**): PDB ID **4FXF**), where black spheres represent the ligand.

**Figure 4:**
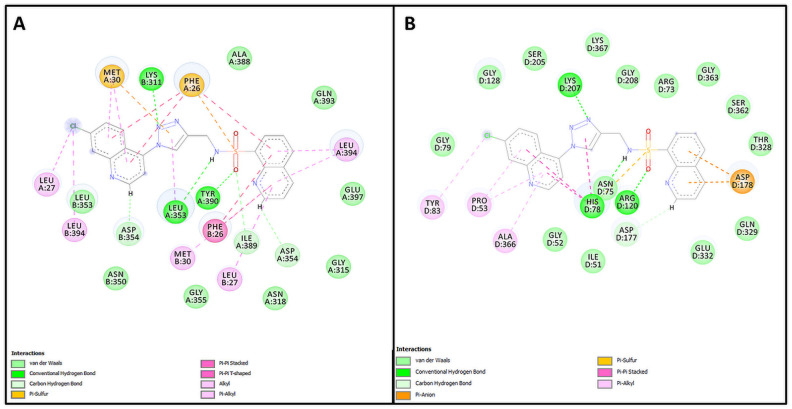
2D representation of docked molecule **9a** and its interactions on the active site of PKM2 ((**A**): PDB ID 4G1N, (**B**): PDB ID 4FXF).

**Figure 5:**
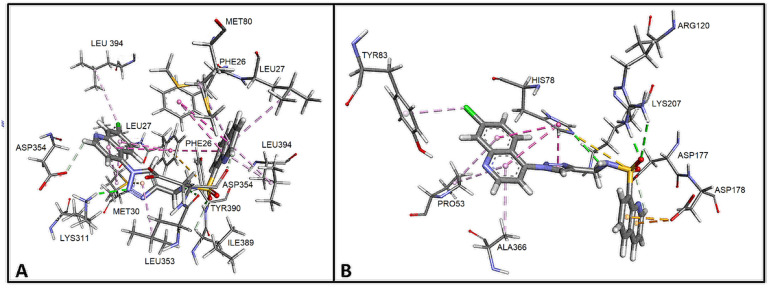
Three-dimensional representation of docked molecule **9a** and its interactions on the active site of PKM2 (**A**: PDB ID 4G1N, **B**: PDB ID 4FXF).

Comparing the **9a** ligand complex obtained in the GOLD program (Figure 3A, Table S1) with the crystallographic structure of 4G1N, it can be concluded that the interactions of the quinoline system with Phe26, Leu27, Met30 and Leu394 were preserved. Hydrogen bonds formed by the sulfonamide moiety of Leu353 and Tyr390 are also present. On the other hand, the hydrogen bond with Lys311, which in the case of ligand **1** was formed by an amide group, in compound **9a** was formed by one of the nitrogen atoms of the triazole system. It should be noted that the complex of compound **9a** is additionally stabilized by the interactions of the second quinolinyl fragment with Phe26, Leu27, and Met30 of the A chain and Leu353, Leu354, and Leu394 of the B chain (Figure 4A and Figure 5A).

An analysis of the binding modes of shikonin [32] showed that shikonin presented a binding pattern with amino acid residue such as Ile51, Asn75, His78, Arg120, and Lys207. Notably, shikonin exhibited π–π stacking interactions with His78. Thus, the binding patterns and modes of compound **5** is essential in controlling the PKM2 inhibitory activity [32].

The analyses of the complex **9a** and 4FXF (Figure 3B, Table S1) included calculations, distance measurements, and pose geometries that determined hydrogen bonding interactions between the pose of the sulfonamide group and the ligand with His78 and Arg120.

* The numbering system of atoms in the betulin fragment is shown in the Figure 10.

Moreover, Lys207 forms another hydrogen bonding with the nitrogen atom of triazole moiety (Figure 4B and Figure 5B). Additionally, His78 forms another interaction with qionoline and the triazole ring (π-π stacking) and with the sulfur atom of the sulfonamide group (π-sulfur). Asp178 forms an interaction with another quinoline moiety (π-anion); in addition, numerous hydrophobic interactions between quinoline moieties and Pro53, Tyr83, and Ala366 (alkyl-π) influence the increase in the stability of the complex, which indicates the high inhibitory potential of derivative **9a.**

**Figure 6:**
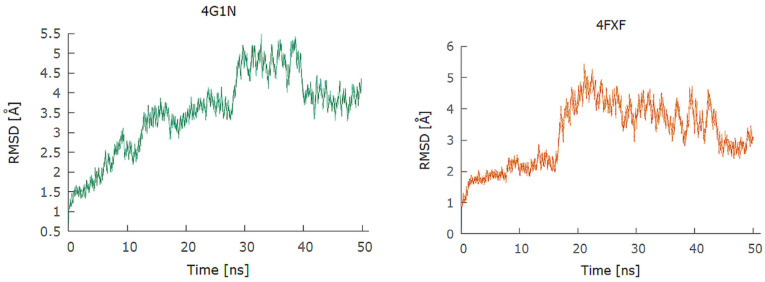
Root mean square deviation (RMSD) of the protein backbone atoms with respect to the equilibrated structure.

In order to verify the results obtained by molecular docking, calculations in the field of molecular dynamics were performed. The lowest energy complexes obtained in the GOLD program were used as input structures in these calculations. To verify the stability of the tests for each component of the system, both protein and ligand, the RMSD values were calculated on the time scale and appropriate graphs were prepared on their basis (Figure 6). RMSD values are expressed in angstroms (Å). The protein RMSD in the **9a**–4G1N complex increased to approximately 3 Å in 30 nanoseconds. In the range of 30 to 40 ns, the RMSD value increased to approx. 5 Å and then decreased and stabilized at approx. 3.5 Å.

In the **9a**–4XFX complex, the RMSD value of the protein increased steadily during the initial 15 ns of simulations (Figure 6) to 2 Å. After exceeding the 18 ns time, the RMSD value of the protein increased to approximately 5 and then, after 40 ns, this value decreased to approximately 3 Å and stabilized.

**Figure 7:**
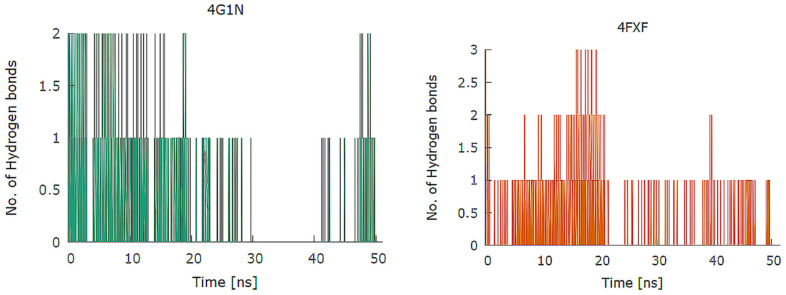
Number of H-bonds formed between protein–ligand complexes with respect to the production period.

Depending on the composition of the analyzed system, the number of formed hydrogen bonds ranged from one to three (Figure 7). In quantitative terms, the maximum number of hydrogen bonds simultaneously occurring at the time of measurement was three for the 4XFX complex and two for the 4G1N complex. Qualitatively, 25 and 31 different hydrogen bonds for 4G1N and 4XFX were detected in the human albumin systems, respectively. The **9a**–4G1N complex is characterized by persistent hydrogen bonds between the ligand and the residues Tyr390 and Lis318. The hydrogen bond of **9a** with the rest of Tyr390 occurred during 3.55% of the duration of the calculations and to Lys311 (1.13%). The key amino acid residues 4XFX for the formation of hydrogen bonds in the **9a**–4XFX complex are Lys367 (2.69% of the duration of the calculations), His78 (1.43%), and Asn75 (1.40%).

The in silico analyses carried out indicate a high potential of **9a**–**e** derivatives to modulate the activity of PKM2.

### 2.2. Chemistry

**Scheme 1:**
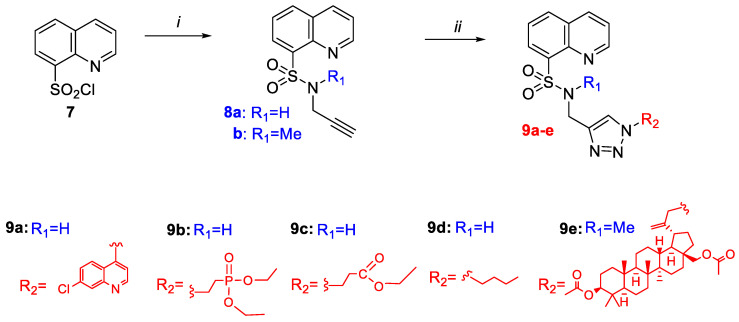
Synthesis of final compounds. Reagents and conditions: (i) 8-quinolinesulfochloride (1 eq), propargyl- or N-methylpropargylamine (2 eq), triethylamine (3 eq), CHCl_3_, 2 h; (ii) Procedure A: 8-quinolinesulfonamide (1 eq), organic azide (1.1. eq), CuSO_4_ · 5 H_2_O, sodium acorbate, DMF/H_2_O, ambient temperature, overnight; Procedure B: organic bromide (1 eq), NaN_3_ (1.2 eq), DMF, ambient temperature, overnight, and then 8-quinolinesulfonamide (1 eq), CuSO_4_ · 5 H_2_O, sodium acorbate, DMF/H_2_O, ambient temperature, overnight.

For the synthesis of triazole derivatives of 8-quinolinesulfonamide, 8-quinolinesulfonyl chloride **7** was used, which was treated with propargylamine or methylpropargylamine (Scheme 1). Sulfonamides **8a**,**b** were then subjected to a CuAAC reaction using copper(II) sulfate and sodium ascorbate as a copper ion reducing agent. Suitable organic azides were used in most cases as the second substrate. The exceptions were substrates butyl, acetate, and phosphonate. In the case of these compounds, because of the instability of their azides, *n*-butyl bromide, ethyl β-bromoacetate, or diethyl (2-bromoethyl)phosphonate and sodium azide were used as the precursors of the corresponding organic azides.

The structure of the obtained compounds **9a**–**e** was confirmed by NMR and HR MS techniques. Thanks to the use of two-dimensional NMR spectra, HSQC and HMBC, it was possible to precisely assign the resonance peaks in the ^13^C NMR spectra to individual carbon atoms. The LC analysis performed confirmed the high purity of the compounds, above 97%.

### 2.3. In Vitro Studies

**Figure 8:**
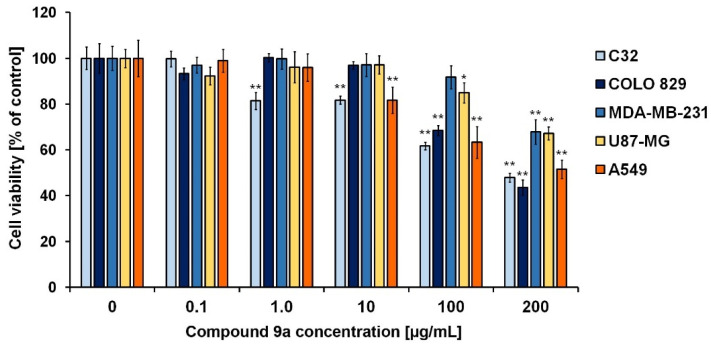
Cell viability data from panels of cancer cell lines treated with compound **9a** in the indicated concentrations for 72 h. Data expressed as a percentage of and presented as the mean values ± SD of three independent experiments. Statistical significance of difference was tested by one-way ANOVA with Dunnet’s post hoc test, * *p* < 0.05, ** *p* < 0.005 vs control.

Basing on the data obtained from in silico studies, a series of new 8-quinolinesulfonamide derivatives (**9a**–**e**) were examined in terms of potential anticancer activity. In the first stage of the in vitro experimental panel, the cytotoxic activity of the new synthesized compounds were tested. The quinolinesulfonamide derivatives were applied in the range of concentrations from 0.1 µg/mL to 200 µg/mL using the 72 h exposure model. In the performed analysis, five cancer cell lines: amelanotic melanoma (C32), melanotic melanoma (COLO829), triple-negative breast cancer (MDA-MB-231), glioblastoma multiforme (U87-MG), and lung cancer (A549) cells, were analyzed. The WST-1 assay revealed that from all the studied quinoline-8-sulfonamides derivatives, compound **9a** exerted high cytotoxicity toward all the studied cancer cell lines, with the corresponding values of the IC50 GI50 parameter found to be: (0.520 mM) 233.9 µg/mL (C32), (0.376 mM) 168.7 µg/mL (COLO829), (0.609) mM 273.5 µg/mL (MDA-MB-231), (0.756 mM) 339.7 µg/mL (U87-MG), and (0.496 mM) 223.1 µg/mL (A549) (Table 2, Figure 8). The new hybrid compounds, except for the derivative 9c, in the studied range of concentrations showed low cytotoxic activity toward normal human dermal fibroblasts, pointing to high selectivity in the mode of action. Moreover, when comparing with the reference compounds, similar cytotoxicity was detected for cisplatin and compound **9a** in the case of lung cancer cells. Therefore, based on the results from the WST-1 assays, compound **9a** was selected for further in vitro analysis with the use of the A549 cell line model.

**Figure 9:**
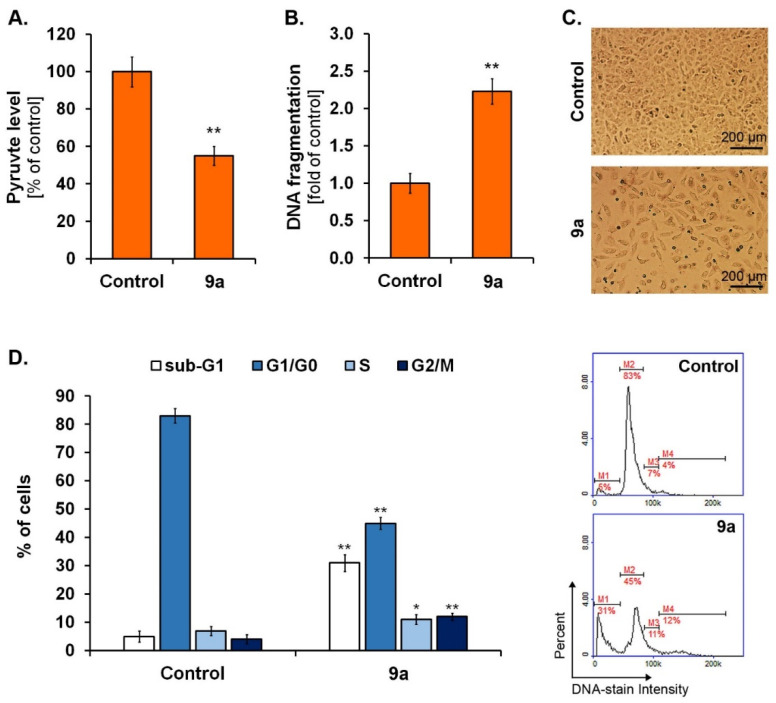
Multidirectional in vitro analysis of A549 cells response to compound **9a**. The effect of 72 h treatment with the compound (200 µg/mL) on an intracellular pyruvate level (**A**); bar graphs represent mean values ± SD of three independent experiments, ** *p* < 0.005 vs control (unpaired t-test). DNA fragmentation in A549 cells treated with compound **9a**. Bar graphs represent mean values ± SD of three independent experiments, ** *p* < 0.005 vs control (unpaired *t*-test) (**B**); representative micrographs (**C**) of A549 cells cultured in T-75 flasks and observed using light-inverted microscope Eclipse TS-100-F (Nikon, Japan). Cell cycle phase distribution of control and **9a**-treated A549 cells (**D**); bar graphs represent mean values ± SD of three independent experiments, * *p* < 0.05, ** *p* < 0.005 vs control (unpaired t-test); representative histograms: M1—cells in sub-G1 phase, M2—cells in G1/G0 phase, M3—cells in S phase, M4—cells in G2/M phase.

Since i/ PKM2 functions as a protein kinase and transcriptional coactivator to promote tumor proliferation and inhibit apoptosis [26], ii/ PKM2 is verified to be highly expressed and secreted in lung cancer cells and clinical samples [37] and iii/ inhibitors that specifically target PKM2 may comprise the new strategy for cancer therapy, we decided to assess the possible modulatory effect of compound **9a** on the intracellular pyruvate level in A549 lung cancer cells. The fluorometric analysis using a 72 h exposure model showed that compound **9a** in the studied concentration (200 µg/mL) reduced intracellular pyruvate level in A549 cells by about 50% compared to the control (Figure 9A). In order to confirm the correlation between PKM2 reduction and inhibitory effect on cell proliferation, microscopic observations were carried out during the exposure of cells to compound **9a**. As shown in Figure 9B, the new quinoline-8-sulfonamide derivatives significantly reduced the number of A549 cells, which indicates a high anti-proliferative effect. Moreover, these findings provide undeniable evidence pointing to the strong structure–activity relationship stated in in silico studies, where the quinolone structure was found to determine a strong capacity to interact with the PKM2 protein. Ning et al. [25] reported the synthesis and biological evaluation of novel naphthoquinone derivatives as selective molecule inhibitors of PKM2. It was noticed that the new compounds inhibited PKM2 activity and thus exerted antiproliferative activity toward a series of cancer cell lines, pointing to the foundation for the development of PKM2-targeted anticancer therapies.

In the last stage of the in vitro experimental panel, we conducted cell cycle and **DNA** fragmentation analysis in A549 lung cancer cells treated with the studied compound. Following the cytometric analysis, it was found compound **9a** caused both G2/M (cell fraction increase from about 4% to about 12%) and S phase blockade (cell fraction increase from about 5% to about 31%) (Figure 9C), confirming both i/ the ability of the tested compound to inhibit A549 cell growth and ii/ the activation of programmed cell death. Simultaneously, we detected about a 2-fold increase in DNA fragmentation (Figure 9B) as a late phase of apoptosis in A549 cells after the treatment with compound **9a**. Similar results were obtained by Ding et al. [38], where cynaropicrin, a natural sesquiterpene lactone compound from artichoke, decreased the cellular PKM2 expression in A549 lung cancer cells and subsequently caused cell cycle arrest and induced DNA fragmentation.

## 3. Materials and Methods

### 3.1. In Silico

The three-dimensional (3D) structures of the compounds studied were generated in their low-energy conformation using the Gaussian 16 computer code [39] in density functional theory (DFT, B3LYP) and 6–311 + G bases (d, p) bases. The target macromolecule for molecular docking studies was obtained from the Protein Data Bank (https://www.rcsb.org/, accessed on 1 February 2023). We used 3D crystal structures of PKM2 (PDB ID: 4G1N and 4FXF). Genetic Optimization for Ligand Docking (GOLD) 2020.1 [40] was used for the docking analysis. The region of interest used for GOLD docking was defined as the PKM2 protein residues within the 10 Å of the reference ATP (X = −4.54, Y = −26.19, Z = 130.75 Å) for 4FXF complex and the reference *N*-(4-{[4-(pyrazin-2-yl)piperazin-1-yl]carbonyl}phenyl)quinoline-8-sulfonamide 1 (X = 1.38, Y = −12.94, Z = 46.80 Å) for the 4G1N complex. Default values of all other parameters were used, and the complexes were submitted to 100 genetic algorithm runs using the GOLDScore fitness function. After calculations, only the ten poses that scored the highest were returned as a docking result for the ligand–cavity configuration. All obtained results were ranked according to their score value and presented in GOLD arbitrary units (a.u.). Molecular docking details were visualized using the BIOVIA Discovery Studio virtual environment [41].

On the basis of docking results, the lowest-energy and best-posed complexes were selected for the molecular dynamics (MD) simulation using Nanoscale Molecular Dynamics software ver. 2.13 (NAMD) [42]. All files were generated using visual molecular dynamics (VMD) [43]. The parameters of the ligands for the force field were obtained from the CGenFF server [44]. The parameterized ligands were inserted into the protein and saved in the form of a protein–ligand complex by user-friendly software, QwikMD [45], with the binding pocket residues. The protein–ligand complex was immersed in the center of a box of water molecules with a TIP3P water box. An amount of 0.15 M ions (Na^+^ and Cl^−^) were added to provide charge neutralization and electrostatic screening. A CHARMM (Chemistry at HARvard Macromolecular Mechanics) 36-parameter file for proteins and lipids and phi and psi cross-term map correction were used in the force field for proteins with similar chemical structures. For the minimization and equilibration of the complexes in the water box, we assumed force field parameters excluding the scaling of 1.0. All atoms, including those of hydrogen, were illustrated explicitly. The preliminary energy was minimized through 2000 steps at constant temperature (310 K), followed by the simulation of an additional 144,000 steps with Langevin dynamics to control the kinetic energy, temperature, and/or pressure of the system. Finally, the system of the solvated protein–ligand complex was equilibrated with 500,000 minimization steps and 25,000,000 runs for 50 ns. The resulted trajectory files obtained with NAMD were analyzed using the Visual Molecular Dynamics package (VMD, https://www.ks.uiuc.edu/Research/vmd/, accessed on 1 February 2023) [43].

### 3.2. Chemistry

#### 3.2.1. General Chemistry Methods

All commercially available reagents were of the highest purity (from Sigma-Aldrich, Fluorochem, and AlfaAesar).

**Figure 10:**
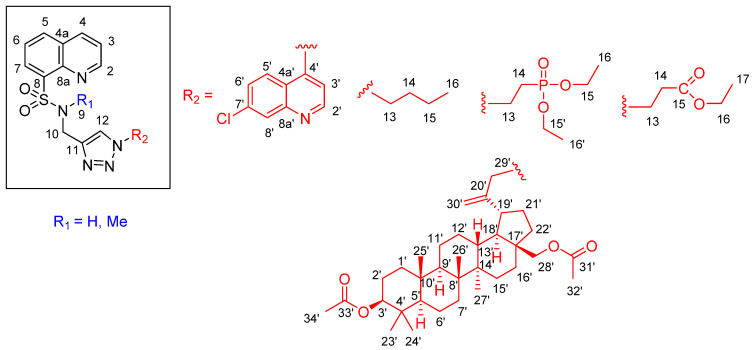
Compounds **9a**–**e** numbering system used in the description of the NMR spectra.

NMR spectra (^1^H, ^13^C, HSQC, and HMBC) were recorded on Bruker Fourier 300 (Bruker Corporation, Billerica, MA, USA) and were reported in ppm using deuterated solvents for calibration (CDCl_3_ or DMSO-*d6*). The *J* values were reported in hertz (Hz), and the splitting patterns were designated as follows: br s. (broad singlet), br d. (broad doublet), s (singlet), d (doublet), t (triplet), q (quartet), dd (doublet of doublets), dt (doublet of triplets), td (triplet of doublets), tt (triplet of triplets), ddd (doublet of doublet of doublets), dq (doublet of quartets), m (multiplet). The following numbering system was used in the description of the NMR spectra (Figure 10):

High-resolution mass spectra were recorded on a Bruker Impact II (Bruker Corporation, Billerica, MA, USA) equipped with an ESI source of ions.

The melting points were uncorrected and measured by an IA 9300 melting point apparatus (Electrothermal).

Thin-layer chromatography (TLC) was performed on 60 F254 silica gel plates (Merck, Darmstadt, Germany) using ethyl acetate as an eluent, and the spots were visualized by UV light (254 nm). All new compounds were purified by column chromatography. Silica gel 60 (Merck, Darmstadt, Germany) was used as a solid phase and ethyl acetate was used as an eluent. LC analysis was performed on a Dionex UltiMate 3000 kit (Thermo Fisher Scientific). An Accucore RP-MS HPLC column (Thermo Fisher Scientific) was used with a length of 150 mm and a diameter of 2.1 mm. ACN/1% aqueous HCOOH 80/20 *v*/*v* solution was used as the eluent. The determinations were made at a wavelength of 277 nm.

The starting compounds 4-azido-7-chloroquinoline and 3,28-di-*O*-acetyl-30-azidobetulin were obtained as described previously [46,47].

#### 3.2.2. Synthesis Sulfonamides (**8a**,**b**)

A solution of 0.126 mL (0.110 g, 2 mmol) of propargylamine or 0.166 mL (0.138 g, 2 mmol) of *N*-methylpropargylamine and 0.417 mL (0.303 g, 3 mmol) of triethylamine in 30 mL of chloroform was cooled to 5 °C. To the solution thus obtained, 0.226 g (1 mmol) of 8-quinolinesulfonyl chloride 7 was added in portions while stirring. The cooling bath was then removed and the mixture stirred for 2 h at room temperature. The contents of the flask were concentrated on a rotary evaporator under reduced pressure. 10 mL of water was added to the resulting residue, and the resulting precipitate was filtered under reduced pressure. The crude product was washed on the filter with 10 mL of water two times and dried in air. Compounds **8a**,**b** were purified by column chromatography and ethyl acetate was used as the eluent phase.

8-N-(prop-2-ynyl)quinolinesulfonamide (**8a**)

Yield: 91 %, m.p. 127–128 °C. ^1^H NMR (DMSO-d_6_, 300 MHz): 2.66 (t, *J* = 2.4 Hz, 1H, H-12), 3.85 (dd, *J* = 6.0 Hz, *J* = 2.4 Hz, 2H, H-10), 7.65–7.76 (m, 3H, H-3, H-6, and H-9), 8.28–8.33 (m, 2H, H-5 and H-7), 8.54 (dd, *J* = 8.4 Hz, *J* = 1.2 Hz, 1H, H-4), 9.08 (dd, *J* = 4.2 Hz, *J* = 1.2 Hz, 1H, H-2). ^13^C NMR (DMSO-d_6_, 75 MHz): 32.9 (C-10), 74.4 (C-12), 79.9 (C-11), 122.9 (C-3), 126.1 (C-6), 128.9 (C-4a), 130.9 (C-5), 134.1 (C-7), 137.5 (C-4), 137.5 (C-8), 143.4 (C-8a), 151.7 (C-2). HRMS (ESI) m/z: C_12_H_11_N_2_O_2_S [M + H]^+^, Calcd. 247.0541; Found 247.0553; C_12_H_10_N_2_NaO_2_S [M + Na]^+^, Calcd. 269.0361; Found 269.0362.

8-N-methyl-N-(prop-2-ynyl)quinolinesulfonamide (**8b**)

Yield: 95 %, m.p. 111–112 °C. ^1^H NMR (CDCl_3_, 300 MHz): 2.02 (t, *J* = 2.4 Hz, 1H, H-12), 3.00 (s, 3H, H-9), 4.40 (d, *J* = 2.4 Hz, 2H, H-10), 7.52 (dd, *J* = 8.4 Hz, *J* = 4.5 Hz, 1H, H-3), 7.63 dd, *J* = 8.1 Hz, *J* = 8.1 Hz, 1H, H-6), 8.05 (dd, *J* = 8.1 Hz, *J* = 1.2 Hz, 1H, H-5), 8.25 (*J* = 8.4 Hz, *J* = 1.8 Hz, 1H, H-4), 8.49 (dd, *J* = 8.1 Hz, *J* = 1.2 Hz, 1H, H-7), 9.06 (dd, *J* = 4.5 Hz, *J* = 1.8 Hz, 1H, H-2). ^13^C NMR (DMSO-d_6_, 75 MHz): 34.6 (C-9), 40.5 (C-10), 72.6 (C-12), 78.3 (C-11), 122.0 (C-3), 125.5 (C-6), 128.9 (C-4a), 133.1 (C-7), 133.6 (C-7), 136.4 (C-4), 136.8 (C-8), 144.2 (C-8a), 151.2 (C-2). HRMS (ESI) m/z: C_13_H_13_N_2_O_2_S [M + H]^+^, Calcd. 261.0698; Found 261.0699.

#### 3.2.3. Synthesis Triazoles (**9a**–**e**)

Procedure A:

To a solution of 0.5 mmol of the respective sulfonamide (**8a** or **8b**) in 5 mL of DMF was added 0.75 mmol of the 4-azido-7-chloroquinoline or 3,28-di-*O*-acetyl-30-azidobetulin. Meanwhile, solutions were prepared: 0.02 g, 0.10 mmol of sodium ascorbate in 0.5 mL of H_2_O and 0.0125 g (0.05 mmol) of CuSO_4_ · 5H_2_O in 0.5 mL of H_2_O. The aqueous solutions were then mixed and added to the DMF solution of the starting materials. The mixture was stirred overnight at room temperature, and then the contents of the flask were poured into 50 mL of water and filtered. Products **9a** or **9e** were purified by column chromatography with SiO_2_ and ethyl acetate was used as mobile phase.

8-N-{[1-(7-chloroquinolin-4-yl)-1H-1,2,3-triazol-4-yl]methyl}quinolinesulfonamide (**9a**)

Yield: 90 %, m.p. 181–182 °C, t_r_ = 0.96 min. ^1^H NMR (DMSO-d_6_, 300 MHz): 4.58 (d, *J* = 6.0 Hz, 2H, H-10), 7.68 (d, *J* = 4.5 Hz, 1H, H-3′), 7.90–8.02 (m, 5H, H-3, H-5′, H-6, H-6′, and H-9), 8.38 (dd, *J* = 8.2 Hz, *J* = 1.2 Hz, 1H, H-5), 8.44–8.51 (m, 3H, H-7, H-8′, and H-12), 8.63 (dd, *J* = 8.1 Hz, *J* = 1.5 Hz, 1H, H-4), 9.23 (dd, *J* = 4.2 Hz, *J* = 1.5 Hz, 1H, H-2), 9.27 (d, *J* = 4.5 Hz, 1H, H-2′). ^13^C NMR (DMSO-d_6_, 75 MHz) d: 38.7 (C-10), 117.0 (C-3′), 120.4 (C-4a’), 122.9 (C-3), 125.8 (C-5′), 126.1 (C-6 and C-12), 128.6 (C-8′), 128.7 (C-4a), 129.3 (C-6′), 131.1 (C-7), 134.1 (C-5), 135.9 (C-7′), 137.1 (C-8), 137.4 (C-4), 140.4 (C-4′), 143.0 (C-8a), 144.4 (C-11), 149.8 (C-8a’), 151.7 (C-2), 152.8 (C-2′). HRMS (ESI) m/z: C_21_H_16_ClN_6_O_2_S [M + H]^+^, Calcd.451.0744; Found 451.0741.

8-N-methyl-N-({1-[3β, 28-diacetoxylup-20(29)-en-30-yl]-1H-1,2,3-triazol-4-yl}methyl)-quinolinesulfonamide (**9e**)

Yield: 71 %, m.p. 136–137 °C, t_r_ = 5.72 min ^1^H NMR (CDCl_3_, 300 MHz): 0.79–0.87 (m, 10H, H-5′, 3xH-23′, 3xH-24′, and 3xH-25′), 0.98 (s, 3H, H-27′), 1.04 (s, 3H, H-26′), 1.05–1.32 (m, 8H, H-1′, H-6′, H-9′, H-11′, H-12′, H-15, H-16′, and H-22′), 1.40–1.50 (m, 5H, H-6′, 2xH-7′, H-11′, H-15′, and H-16′), 1.63–1.89 (m, 9H, H-1′, 2xH-2′, H-12′, H-13′, H-16′, H-18′, H-21′, H-22′), 2.05 (s, 3H, 3xH-34′), 2.08 (s, 3H, 3xH-32′), 2.27-2.38 (m, 1H, H-19′), 2.89 (s, 3H, 3xH-9), 3.50 (s, 2H, 2xH-10), 3.74 (d, *J* = 10.8 Hz, 1H, H-28′), 4.24-4.63 (m, 3H, H-10 and 2xH-28′), 4.85-5.05 (m, 5H, H-3, 2xH-29,’ and 2xH-30′), 7.56 (dd, t, *J* = 8.4 Hz, *J* = 4.2 Hz, 1H, H-3), 7.66 (dd, *J* = 8.4 Hz, *J* = 7.5 1H, H-6), 7.71 (s, 1H, H-12), 8.07 (dd, *J* = 8.4 Hz, *J* = 1.2 Hz, 1H, H-5), 8.30 (dd, *J* = 8.4 Hz, *J* = 1.5 Hz, 1H, H-4), 8.51 (dd, *J* = 7.5 Hz, *J* = 1.2 Hz, 1H, H-7), 9.09 (dd, *J* = 4.2 Hz, *J* = 1.5 Hz, 1H, H-2). ^13^C NMR (CDCl_3_, 75 MHz) d: 14.7 (C-27′), 16.0 (C-24′), 16.2 (C-26′), 16.5 (C-25′), 18.1 (C-6′), 20.9 (C-11′), 21.1 (C-32′), 21.4 (C-34′) 23.7 (C-2′), 26.8 (C-12′), 27.0 (C-15′), 28.0 (C-23′), 29.8 (C-16′), 31.2 (C-21′), 34.1 (C-7′), 34.4 (C-22′), 35.0 (C-9), 37.1 (C-10′), 37.4 (C-13′), 37.8 (C-4′), 38.4 (C-1′), 40.9 (C-8′), 42.7 (C-14′), 43.9 (C-19′), 46.4 (C-17′), 46.7 (C-9), 49.9 (C-18′), 50.1 (C-9′), 54.6 (C-30′), 55.3 (C-5′), 62.4 (C-28′), 80.9 (C-3′), 122.2 (C-3), 123.5 (C-12), 125.6 (C-6), 129.0 (C-4a), 133.2 (C-7), 133.6 (C-5), 136.6 (C-4), 136.9 (C-8), 144.1 (C-8a), 145.5 (C-11), 148.8 (C-20′), 151.3 (C-2), 171.0 (C-33′), 171.5 (C-31′). HRMS (ESI) m/z: C_47_H_66_N_5_O_6_S [M + H]^+^, Calcd. 828.4734; Found 828.4732.

#### 3.2.4. Procedure B

To a solution of 0.5 mmol of the respective bromide in 5 mL of DMF, 0.036 g (0.55 mmol) of the sodium azide was added. The mixture was stirred overnight at room temperature, and then respective sulfonamide (0.5 mmol) was added. Meanwhile, solutions were prepared: 0.02 g (0.10 mmol) of sodium ascorbate in 0.5 mL of H_2_O and 0.0125 g (0.05 mmol) of CuSO_4_ · 5H_2_O in 0.5 mL of H_2_O. The aqueous solutions were then mixed and added to the DMF solution of the starting materials. The mixture was stirred overnight at room temperature, and then the contents of the flask were poured into 50 mL of water and filtered. Products **9b**–**d** were purified by column chromatography with SiO_2_, and ethyl acetate was used as mobile phase.

Diethyl 2-{4-[methyl-(8-sulfamoylquinolyl)-1H-1,2,3-triazol-1-yl]}ethylphosphonate (**9b**)

Yield: 85 %, oil, t_r_ = 0.84 min. ^1^H NMR (DMSO-d_6_, 300 MHz): 1.20 (t, *J* = 7.2 Hz, 6H, H-16 and H-16′), 2.20 (dt, ^2^*J*_P-H_ = 18.6 Hz, *J* = 7.8 Hz 2H, H-14), 3.96 (dq, *J* = 7.2 Hz, ^3^*J*_P-H_ = 2.4 Hz, 2H,, 4H, H-15 and H-15′), 4.17 (d, *J* = 6.0 Hz, 2H, H-10), 4.25 (dt, *J* = 7.8 Hz, ^3^*J*_P-H_ = 3.3 Hz, 2H, H-13), 7.62 (t, *J* = 6.0 Hz, 1H, H-9), 7.68-7.76 (m, 3H, H-3, H-6, and H-12), 8.25-8-29 (m, 2H, H-5 and H-7), 8.52 (dd, *J* = 8.1 Hz, *J* = 1.8 Hz, 1H, H-4), 9.05 (dd, *J* = 4.2 Hz, *J* = 1.8 Hz, 1H, H-2). ^13^C NMR (DMSO-d_6_, 75 MHz) d:16.6 (d, ^3^*J*_C-P_ = 6 Hz, C-16 and C-16′), 26.4 (d, ^1^*J*_C-P_ = 137.3 Hz, C-14), 39.0 (C-10), 44.0 (C-13), 61.8 (d, ^2^*J*_C-P_ = 6 Hz, C-15 and C-15′), 122.9 (C-3), 123.6 (C-12), 126.1 (C-6), 128.8 (C-4a), 131.0 (C-7), 134.0 (C-5), 137.0 (C-8), 137.4 (C-4), 143.0 (C-8a), 143.8 (C-11), 151.7 (C-2). HRMS (ESI) m/z: C_18_H_24_N_5_NaO_5_PS [M + Na]^+^, Calcd. 476.1133; Found 476.1136.

Ethyl 3-{[4-(8-sulfamoylquinolyl)methyl]-1H-1,2,3-triazol-1-yl}propanoate (**9c**)

Yield: 81 %, oil, t_r_ = 0.87 min. ^1^H NMR (DMSO-d_6_, 300 MHz): 1.20 (t, *J* = 7.2 Hz, 3H, H-17), 2.77 (t, *J* = 6.9 Hz, 2H, H-13), 4.04 (q, *J* = 7.2 Hz, 2H, H-16), 4.17 (d, *J* = 6.0 Hz, 2H, H-10), 4.34 (t, *J* = 6.9 Hz, 2H, H-14), 7.66-7.74 (m, 4H, H-3, H-6, H-9, and H-12), 8.25-8.31 (m, 2H, H-5 and H-7), 8.52 (dd, *J* = 8.1 Hz, *J* = 1.8 Hz, 1H, H-4), 9.05 (dd, *J* = 4.2 Hz, *J* = 1.8 Hz, 1H, H-2). ^13^C NMR (DMSO-d_6_, 75 MHz) d:14.5 (C-17), 34.3 (C-13), 39.0 (C-10), 45.3 (C-14), 60.8 (C-16), 122.9 (C-3), 123.7 (C-12), 126.1 (C-6), 128.8 (C-4a), 131.0 (C-7), 134.0 (C-5), 137.0 (C-8), 137.4 (C-4), 143.0 (C-8a), 143.8 (C-11), 151.7 (C-2), 170.7 (C-15). HRMS (ESI) m/z: C_17_H_20_N_5_O_4_S [M + H]^+^, Calcd. 390.1236; Found 390.1239.

8-N-{[1-(1-butyl)-1H-1,2,3-triazol-4-yl]methyl}quinolinesulfonamide (**9d**)

Yield: 94 %, m.p. 80-81 °C, t_r_ = 0.90 min. ^1^H NMR (DMSO-d_6_, 300 MHz): 0.83 (t, *J* = 7.2 Hz, 3H, H-16), 1.06 (sext, *J* = 7.2 Hz, 2H, H-15), 1.50 (quint, *J* = 7.2 Hz, 2H, H-14), 4.07 (t, *J* = 7.2 Hz, 2H, H-13), 4.18 (d, *J* = 6.0 Hz, 2H, H-10), 7.56 (s, 1H, H-12), 7.64-7.73 (m, 3H, H-3, H-6, and H-9), 8.24-8.32 (m, 2H, H-5 and H-7), 8.51 (dd, *J* = 8.1 Hz, *J* = 1.8 Hz, 1H, H-4), 9.04 (dd, *J* = 4.2 Hz, *J* = 1.8 Hz, 1H, H-2). ^13^C NMR (DMSO-d_6_, 75 MHz) d:13.7 (C-16), 19.4 (C-15), 32.0 (C-14), 390 (C-10), 49.1 (C-13), 122.9 (C-3), 123.2 (C-12), 126.1 (C-6), 128.8 (C-4a), 130.9 (C-7), 134.0 (C-5), 137.0 (C-8), 137.4 (C-4), 143.0 (C-8a), 143.6 (C-11), 151.7 (C-2). HRMS (ESI) m/z: C_16_H_20_N_5_O_2_S [M + H]^+^, Calcd. 346.1338; Found 346.1336.

### 3.3. In Vitro Study

#### 3.3.1. Chemicals

Dulbecco’s phosphate-buffered saline, Halt Protease Inhibitor Cocktail, Halt Phosphatase Inhibitor Single-Use Cocktail, Pierce BCA Protein Assay Kit, and Trypsin/EDTA solution were purchased from Thermo Fisher Scientific (Waltham, MA, USA). Amphotericin B, penicillin G, and RIPA Buffer were purchased from Sigma-Aldrich Inc. (St. Luis, MO, USA). NC-Slides A8, Solution 3, and Via1-Cassettes were obtained from ChemoMetec (Lillerød, Denmark). Growth media (DMEM and RPMI 1640) and fetal bovine serum were acquired from CytoGen (Zgierz, Poland). Neomycin sulfate was obtained from 1Amara (Kraków, Poland). The remaining chemicals were purchased from POCH S.A. (Gliwice, Poland).

#### 3.3.2. Cell Culture

All tested cell lines were purchased from ATCC (USA). C32 cells (human amelanotic melanoma), MDA-MB-321 cells (human breast carcinoma), U87-MG cells (human glioblastoma), and A549 cells (human lung carcinoma) were cultured in DMEM medium. COLO 829 cells (human melanotic melanoma) were cultured in RPMI 1640 medium. Both media were supplemented with 10% fetal bovine serum, penicillin (100 U/mL), neomycin (10 µg/mL), and amphotericin B (0.25 µg/mL). Cells were maintained at 37 °C in a 5% CO_2_ incubator.

#### 3.3.3. WST-1 Assay

The impact of compounds **9a**–**e** on C32, COLO 829, MDA-MB-321, U87-MG, and A549 cell viability was assessed using a WST-1 reagent (Roche Diagnostics GmbH, Germany) according to the manufacturer’s protocol. In brief, cells were seeded into 96-well microplates (2.5 × 10^3^ cells/well) and grown for 24 h. Then, the cells were treated with the compounds in the concentration range from 0.1–200 µg/mL 

(0.444 mM) for 72 h. WST-1 reagent was added 2 h before the end of the incubation time. The absorbance was measured at 440 nm (with reference wavelength of 650 nm) using a microplate reader Infinite 200 Pro. GI_50_ values were extrapolated from the obtained cell viability data using GraphPad Prism software.

#### 3.3.4. Quantitative Analysis of Cellular Pyruvate Level

The impact of **9a** on pyruvate kinase activity was determined using commercially available enzyme assay kit: Pyruvate Assay Kit (Cat. No. ab65342; Abcam, USA), according to the manufacturer’s instruction. In brief, pyruvate concentration in cell lysates obtained from A549 cells treated with compound **9a** (200 µg/mL, 0.444 mM) for 72 h was measured by a coupled enzyme assay, which resulted in a fluorometric (λex = 535/λem = 587 nm) product, proportional to the pyruvate present. The fluorescence of the samples was measured using a microplate reader Infinite 200 Pro. The results were calculated per mg of protein and expressed as percentage of the untreated control.

#### 3.3.5. DNA Fragmentation and Cell Cycle Assay

After treatment of A549 cells with 9a in a concentration 200 μg/mL (0.444 mM) for 72 h, the cells were analyzed in terms of cell cycle progression using the fluorescence image cytometer NucleoCounter NC-3000 (Chemometec, Denmark), according to the previously described method [48]. In brief, the cells following fixation were stained with DAPI (Solution 3 containing 1 μg/mL DAPI and Triton X-100 in PBS) and analyzed using NucleoView NC-3000 software (ChemoMetec, Denmark).

## 4. Conclusions

Herein, for the first time, we synthesized new quinoline-8-sulfonamide derivatives with the capacity to inhibit the tumor-cell-specific M2 isoform of pyruvate kinase. Basing on both in silico and in vitro studies, the compound **9a**, in contrast to other studied derivatives, was characterized by an improved profile against the tumor-cell-specific M2 isoform of pyruvate kinase as a biological pathway target, and thus exerted ability to reduce lung cancer cell viability and apoptosis induction. Moreover, we demonstrated that the introduction of another quinolinyl fragment to the modulator molecule may have a significant impact on the greater activity of the **9a** derivative compared to other compounds. In addition, it may comprise the basis and further directions for extensive research to find novel analogs suitable for clinical applications in cancer treatment.

## Figures and Tables

**Table 1:** Molecular docking results obtained with GOLD for PKM2 modulators (**1**–**6**) and newly designed 8-quinolinesulfonamide derivatives (**9a**–**e**).

Compound	PKM2 Structure			
	PDB ID: 4G1N		PDB ID: 4XFX	
	GOLD Score [a.u.]	ΔG [kcal/mol]	GOLD Score [a.u.]	ΔG [kcal/mol]
**1**	75.03	−10.34	65.82	−9.35
**2**	62.95	−9.04	54.05	−8.08
**3**	63.77	−9.13	56.78	−8.38
**4**	62.99	−9.04	62.30	−8.97
**5**	58.05	−8.51	53.64	−8.04
**6**	60.65	−8.79	52.92	−7.96
**9a**	78.56	−10.72	76.33	−10.48
**9b**	72.64	−10.08	75.57	−10.40
**9c**	73.17	−10.14	64.99	−9.26
**9d**	70.82	−9.89	61.35	−8.87
**9e**	71.35	−9.94	60.36	−8.76

**Table 2:** The anticancer potential of synthesized compounds **9a**–**e** expressed as GI50, i.e., the concentration inhibiting cell growth by 50% as compared with control cells.

Compound	Analyzed Cell Lines—GI_50_					
	C32	COLO 829	MDA-MB-231	U87-MG	A549	NHDF
**9a**	234 µg/mL (0.520 mM)	169 µg/mL (0.376 mM)	274 µg/mL (0.609 mM)	340 µg/mL (0.756 mM)	223 µg/mL (0.496 mM)	Negative
**9b**	200 µg/mL (0.441 mM)	266 µg/mL (0.587 mM)	Negative	347 µg/mL (0.765 mM)	Negative	Negative
**9c**	269 µg/mL (0.691 mM)	179 µg/mL (0.460 mM)	Negative	Negative	Negative	87 µg/mL (0.223 mM)
**9d**	209 µg/mL (0.605 mM)	130 µg/mL (0.376 mM)	Negative	328 µg/mL (0.949 mM)	Negative	Negative
**9e**	303 µg/mL (0.373 mM)	Negative	314 µg/mL (0.386 mM)	Negative	Negative	Negative
**Reference compound**	Dacarbazine 9 µg/mL (0.049 mM) [34]	Dacarbazine negative [35]	Cisplatin 4 µg/mL (0.013 mM)	Temozolomide negative [36]	Cisplatin 196 µg/mL (0.653 mM)	N/A

C32: amelanotic melanoma, COLO 829: melanotic melanoma, MDA-MB-231: triple-negative breast cancer, U87-MG: glioblastoma multiforme, A549: lung cancer.

## Data Availability

The data presented in this study are available in the Supplementary Materials.

## Supplementary Materials

The following supporting information can be downloaded at https://www.mdpi.com/article/10.3390/molecules28062509/s1. Interaction of compounds **9a***–***e** with target proteins, (Table S1), HR MS, ^1^H NMR, ^13^C NMR, HSQC, and HMBC spectra of all intermediates and final compounds (Figures S1–S35) and HPLC analysis of compouds **9a**–**e** (Figures S36–S40).

## Sample Availability

Samples of the compounds are available from the authors.
