# Supplementary material for: Design, Synthesis and Biological Evaluation of Quinoline-8-Sulfonamides as Inhibitors of the Tumor Cell-Specific M2 Isoform of Pyruvate Kinase: Preliminary Study
Source: Molecules. 2023 Mar 9;28(6):2509. doi: 10.3390/molecules28062509 (PMC10058396; doi:10.3390/molecules28062509)
Supplement: Supplementary file 1 [file molecules-28-02509-s001.zip › molecules-2227264-supplementary.pdf]

Design, synthesis, and biological evaluation of quinoline-8-sulfonamides as inhibitors of the tumor cell specific M2 isoform of pyruvate kinase

Krzysztof Marciniec [1,*], Artur Beberok [2], Zuzanna Rzepka [2], Elwira Chrobak [1], Małgorzata Latocha [3], Dorota Wrześniok [2], and Stanisław Boryczka[1]

[1] Department of Organic Chemistry, Medical University of Silesia, Jagiellońska 4, 41-200 Sosnowiec, Poland
[2] Department of Pharmaceutical Chemistry, Jagiellońska 4, 41-200 Sosnowiec, Poland
[3] Department of Molecular Biology, Jagiellońska 4, 41-200 Sosnowiec, Poland

Correspondence: kmarciniec@um.edu.pl

Table S1. Interaction of compounds **9a**–**e** with target proteins

Protein		Ligand		Interaction	
Name	Residue	Name	Residue	Type	Distance [Å]
**G1N**	A:Tyr390	**9a**	Sulfonamide oxygen	Conventional hydrogen bond	1.76
	B:Lys311		Triazole nitrogen	Conventional hydrogen bond	2.67
	A:Leu353		Sulfonamide nitrogen	Conventional hydrogen bond	2.42
	A:Ile389		Sulfonamide oxygen	Carbon hydrogen bond	2.75
	A:Asp354		Pyridine ring C2	Carbon hydrogen bond	2.29
	B:Asp354		Pyridine ring C2′	Carbon hydrogen bond	2.82
	A:Met30		Triazole ring	π-Sulfur	4.07
	A:Phe26		Benzene ring	π-Sulfur	5.89
	A:Phe26		Benzene ring	π-π- Stacked	5.36
	A:Phe26		Pyridine ring	π-π T-shaped	5.94
	A:Phe26		Benzene ring	π-π T-shaped	4.53
	A:Phe26		Benzene ring	π-π T-shaped	5.18
	A:Phe26		Pyridine ring	π-π T-shaped	5.13
	A:Leu27		Chlorine atom	Alkyl	4.42
	B:Leu394		Chlorine atom	Alkyl	5.43
	A:Leu394		Benzene ring	π-Alkyl	4.44
	A:Leu394		Pyridine ring	π-Alkyl	4.87
	B:Leu27		Pyridine ring	π-Alkyl	5.36
	B:Met30		Pyridine ring	π-Alkyl	5.49
	A:Leu353		Triazole ring	π-Alkyl	4.41
	A:Met30		Benzene ring	π-Alkyl	5.13
	A:Met30		Pyridine ring	π-Alkyl	4.12
	A:Tyr390	**9b**	Sulfonamide oxygen	Conventional hydrogen bond	1.97
	A:Leu353		Sulfonamide oxygen	Carbon hydrogen bond	2.58
	A:Ile389		Sulfonamide oxygen	Carbon hydrogen bond	2.35
	A:Leu353		Pyridine ring C2	Carbon hydrogen bond	2.70
	A:Asp354		Pyridine ring C2	Carbon hydrogen bond	2.27
	B:Leu353		Ethyl group	Carbon hydrogen bond	2.73
	A:Phe26		Sulfonamide sulfur	π-Sulfur	5.16
	B:Phe26		Benzene ring	π-π T-shaped	5.45
	B:Phe26		Pyridine ring	π-π T-shaped	5.14
	B:Leu394		Ethyl group	Alkyl	4.66
	B:Leu394		Ethyl group	Alkyl	4.87
	A:Leu394		Benzene ring	π-Alkyl	4.48
	A:Leu394		Pyridine ring	π-Alkyl	5.20
	A:Met30		Triazole ring	π-Alkyl	4.59
	A:Leu353		Triazole ring	π-Alkyl	5.44
	B:Lys311		Triazole ring	π-Alkyl	4.86
	B:Lys311	**9c**	Sulfonamide oxygen	Conventional hydrogen bond	1.95
	B:Leu394		Ester carbonyl oxygen	Carbon hydrogen bond	2.42
	A:Leu353		Benzene ring	π -Sigma	2.47
	A:Met30		Triazole ring	π -Sulfur	4.82
	A:Phe26		Triazole ring	π-π-Stacked	3.16
	A:Phe26		Benzene ring	π-π T-shaped	4.80
	B:Phe26		Triazole ring	π-π T-shaped	5.38
	A:Leu27		Ethyl group	Alkyl	4.64

	B:Leu394		Ethyl group	Alkyl	5.44
	A:Leu353		Pyridine ring	π-Alkyl	3.97
	B:Lys311	9d	Sulfonamide oxygen	Conventional Hydrogen Bond	1.83
	A:Met30		Triazole ring	π-Sulfur	4.95
	A:Phe26		Triazole ring	π-π-Stacked	3.33
	A:Phe26		Benzene ring	π-π T-shaped	5.01
	A:Phe26		Pyridine ring	π-π T-shaped	5.21
	B:Phe26		Triazole ring	π-π T-shaped	5.37
	B:Ile389		Butyl group	Alkyl	4.77
	B:Leu394		Butyl group	Alkyl	4.07
	A:Leu353		Benzene ring	π-Alkyl	3.79
	A:Leu353		Pyridine ring	π-Alkyl	4.37
	A:Lys311	9e	Ester carbonyl oxygen	Conventional hydrogen bond	1.55
	A:Leu353		Betuline C29′*	Carbon hydrogen bond	2.01
	A:Leu353		Betuline C29′	Carbon hydrogen bond	1.87
	B:Asn350		Betuline C28′	Carbon hydrogen bond	2.16
	A:Gln393		Sulfonamide methyl group	Carbon hydrogen bond	2.32
	A:Leu394		Pyridine ring	π-Sigma	2.26
	B:Met30		Triazole ring	π-Sulfur	5.70
	B:Met30		Benzene ring	π-Sulfur	4.13
	B:Met30		Pyridine ring	π-Sulfur	5.67
	B:Phe26		Sulfonamide sulfur	π-Sulfur	5.76
	B:Leu353		Betuline C15′	Alkyl	5.29
	B:Leu394		Betuline C1′	Alkyl	5.45
	A:Leu353		Betuline C21′	Alkyl	4.30
	A:Leu27		Betuline C23′	Alkyl	4.84
	B:Leu394		Betuline C23′	Alkyl	3.82
	B:Leu394		Betuline C24′	Alkyl	3.02
	B:Leu353		Betuline C25′	Alkyl	4.79
	A:Met30		Betuline C27′	Alkyl	4.61
	A:Leu353		Betuline C30′	Alkyl	3.44
	A:Phe26		Betuline C1′	π-Alkyl	4.90
	A:Phe26		Betuline C11′	π-Alkyl	4.81
	B:Phe26		Betuline C12′	π-Alkyl	4.72
	B:Phe26		Betuline C25′	π-Alkyl	4.35
	B:Phe26		Betuline C26′	π-Alkyl	4.76
	A:Leu394		Benzene ring	π-Alkyl	4.88
	B:Leu27		Benzene ring	π-Alkyl	5.08
**4FXF**	A:Arg120	**9a**	Sulfonamide oxygen	Conventional hydrogen bond	2.82
	A:Arg120		Sulfonamide oxygen	Conventional hydrogen bond	1.67
	A:Lys207		Triazole nitrogen	Conventional hydrogen bond	2.10
	A:His78		Sulfonamide nitrogen	Conventional hydrogen bond	2.73
	A:Asp177		Pyridine ring C2	Carbon hydrogen bond	2.65
	A:Asp178		Benzene ring	π-Anion	3.21
	A:Asp178		Pyridine ring	π-Anion	2.64
	A:His78		Sulfonamide sulfur	π-Sulfur	5.74
	A:His78		Triazole ring	π-π-Stacked	3.29
	A:His78		Pyridine ring	π-π-Stacked	4.47
	A:His78		Benzene ring	π-π-Stacked	4.43
	A:Tyr83		Chlorine atom	π-Alkyl	4.41
	A:Pro53		Pyridine ring	π-Alkyl	5.19
	A:Ala366		Pyridine ring	π-Alkyl	4.66

A:Pro53		Benzene ring	π-Alkyl	4.93
A:Arg73	**9b**	Phosphonate oxygen	Conventional hydrogen bond	1.81
A:Arg73		Phosphonate oxygen	Conventional hydrogen bond	3.09
A:Ser205		Sulfonamide oxygen	Conventional hydrogen bond	3.03
A:Ser205		Sulfonamide oxygen	Conventional hydrogen bond	2.63
A:Lys207		Sulfonamide oxygen	Carbon hydrogen bond	2.15
A:Arg120		Triazole ring	π-Cation	4.00
A:His78		Pyridine ring	π-π-Stacked	3.62
A:His78		Benzene ring	π-π-Stacked	3.57
A:His78		Triazole ring	π-π-Stacked	5.30
A:Ala293		Ethyl	Alkyl	4.06
A:Ala327		Ethyl	Alkyl	4.34
A:Met291		Ethyl	Alkyl	4.76
A:Met360		Ethyl	Alkyl	5.24
A:Phe244		Ethyl	π-Alkyl	5.26
A:Pro53		Pyridine ring	π-Alkyl	5.44
A:Ala366		Benzene ring	π-Alkyl	4.83
A:Arg73	**9c**	Ester carbonyl oxygen	Conventional hydrogen bond	2.09
A:Arg73		Triazole nitrogen	Conventional hydrogen bond	1.65
A:Arg73		Ester carbonyl oxygen	Conventional hydrogen bond	2.86
A:Arg120		Ester oxygen	Conventional hydrogen bond	2.73
A:Lys207		Sulfonamide oxygen	Conventional hydrogen bond	2.38
A:His78		Sulfonamide nitrogen	Conventional hydrogen bond	2.86
A:Asn75		Ethyl group	Carbon hydrogen bond	2.15
A:Asp113		Ethyl group	Carbon hydrogen bond	3.07
A:Asp113		Ethyl group	Carbon hydrogen bond	2.63
A:His78		Sulfonamide sulfur	π-Sulfur	4.43
A:His78		Pyridine ring	π-π-Stacked	3.97
A:His78		Benzene ring	π-π-Stacked	4.93
A:Pro53		Pyridine ring	π-Alkyl	5.19
A:Pro53		Benzene ring	π-Alkyl	4.62
A:Ala366		Pyridine ring	π-Alkyl	5.23
A:Arg73	**9d**	Triazole nitrogen	Conventional hydrogen bond	2.70
A:Arg73		Triazole nitrogen	Conventional hydrogen bond	2.05
A:Lys207		Sulfonamide oxygen	Conventional hydrogen bond	1.72
A:His78		Sulfonamide nitrogen	Conventional hydrogen bond	2.02
A:His78		Sulfonamide oxygen	Carbon hydrogen bond	2.95
A:His78		Methylene linker	Carbon hydrogen bond	2.92
A:His78		Benzene ring	π-π-Stacked	3.14
A:His78		Pyridine ring	π-π-Stacked	3.80
A:Phe244		Butyl group	π-Alkyl	5.39
A:Ala366		Pyridine ring	π-Alkyl	4.77
A:Lys367	**9e**	Triazole nitrogen	Conventional hydrogen bond	2.22
B:Lys337		Sulfonamide oxygen	Carbon hydrogen bond	2.89
A:Thr129		Betuline C33′	Carbon hydrogen bond	2.20
A:Lys367		Triazole nitrogen	Carbon hydrogen bond	2.53
A:Asp177		Betuline C29′	Carbon hydrogen bond	2.82
A:Glu332		Betuline C29′	Carbon hydrogen bond	3.07
A:Asp178		Betuline C28′	Carbon hydrogen bond	2.64
A:Glu332		Triazole carbon	Carbon hydrogen bond	2.71
B:Lys337		Benzene ring	π-Cation	3.55
B:Lys337		Pyridine ring	π-Cation	3.99

A:His78	Betuline C6′	π-Sigma	2.09
A:Pro53	Betuline C1′	Alkyl	4.93
A:Ala366	Betuline C27′	Alkyl	3.82
A:Lys367	Betuline C1′	Alkyl	4.80
A:Lys367	Betuline C10′	Alkyl	5.44
A:Lys367	Betuline C11′	Alkyl	4.80
A:Pro53	Betuline C23′	Alkyl	3.90
A:Ile335	Betuline C30′	Alkyl	4.04
A:His78	Betuline C5′	π-Alkyl	4.85
A:His78	Betuline C24′	π-Alkyl	3.86
A:His78	Betuline C25′	π-Alkyl	3.36
A:His78	Betuline C26′	π-Alkyl	3.33
A:Ile335	Triazole ring	π-Alkyl	4.94
B:Lys337	Benzene ring	π-Alkyl	5.08
B:Lys336	Pyridine ring	π-Alkyl	4.84

Compound Spectrum List Report

Analysis Info

Analysis Name	D:\Data\Service_20220831\8QSAProp2.d	Acquisition Date	9/13/2022 1:13:51 PM
Method	low_mass.m	Operator	KM
Sample Name	tune_poshigh_test1	Instrument	impact II 1825265.10082
Comment			

Acquisition Parameter

Source Type	ESI	Ion Polarity	Positive	Set Nebulizer	0.3 Bar
Focus	Active	Set Capillary	4000 V	Set Dry Heater	200 °C
Scan Begin	100 m/z	Set End Plate Offset	-500 V	Set Dry Gas	3.0 l/min
Scan End	1000 m/z	Set Charging Voltage	2000 V	Set Divert Valve	Source
		Set Corona	0 nA	Set APCI Heater	0 °C

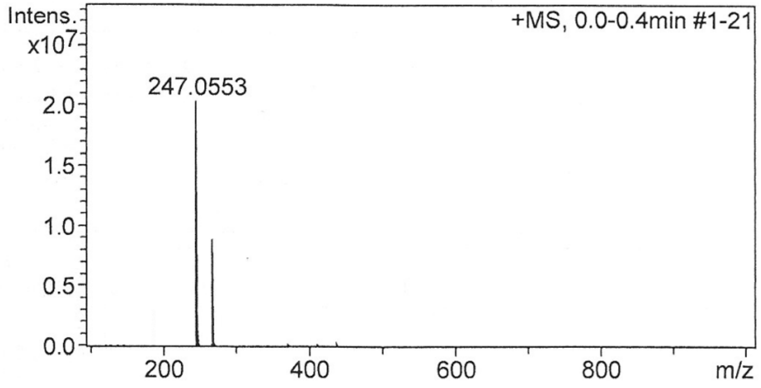

#	m/z	Res.	S/N	I	I %	FWHM
1	247.0553	15207	81856.7	20377568	100.0	0.0162
2	269.0362	36498	31220.0	8915149	43.7	0.0074

Figure S1. HR MS spectra of 8-*N*-(prop-2-ynyl)quinolinesulfonamide (**8a**)

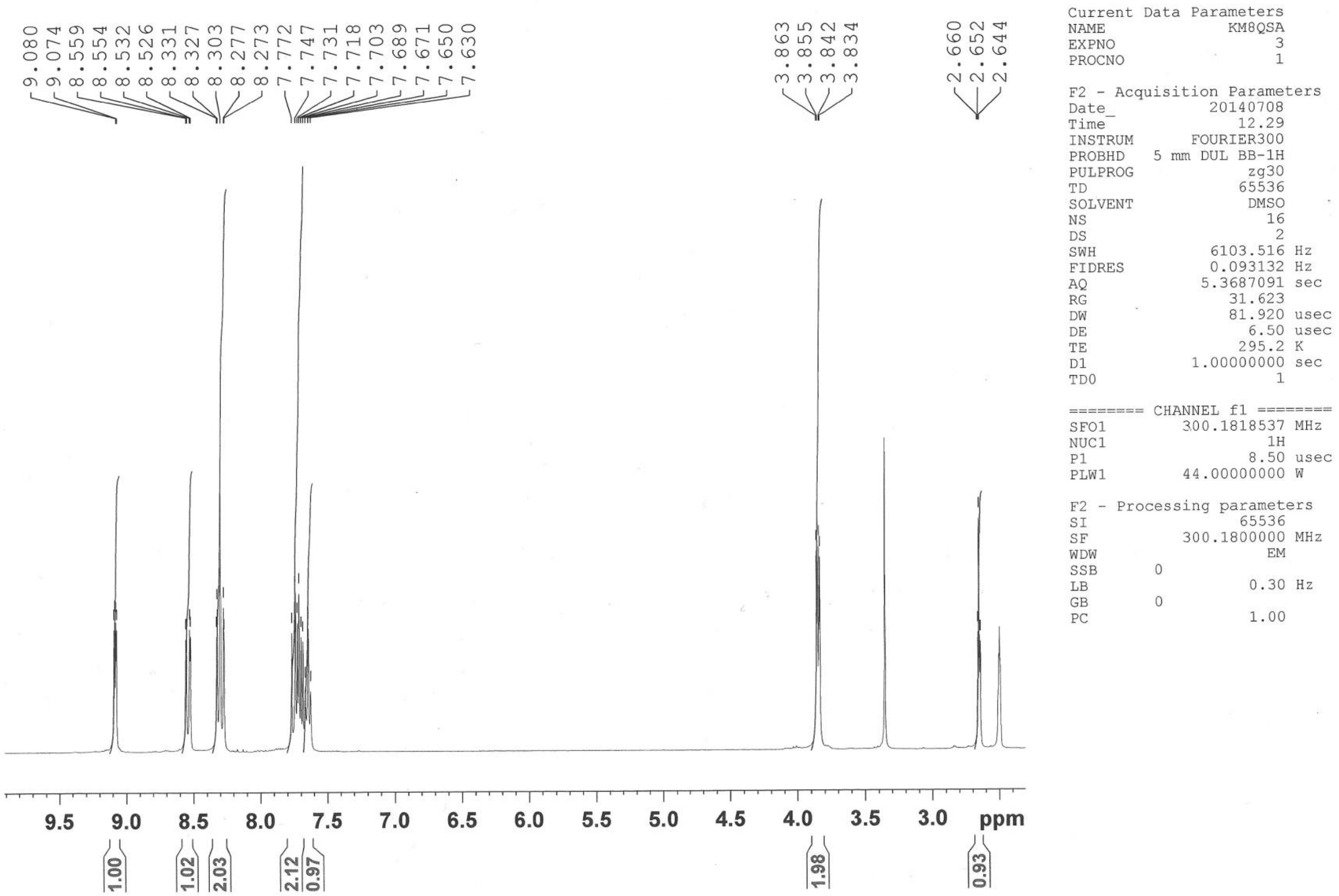

Figure S2. ^{1}H NMR spectra of 8-*N*-(prop-2-ynyl)quinolinesulfonamide **(8a)**

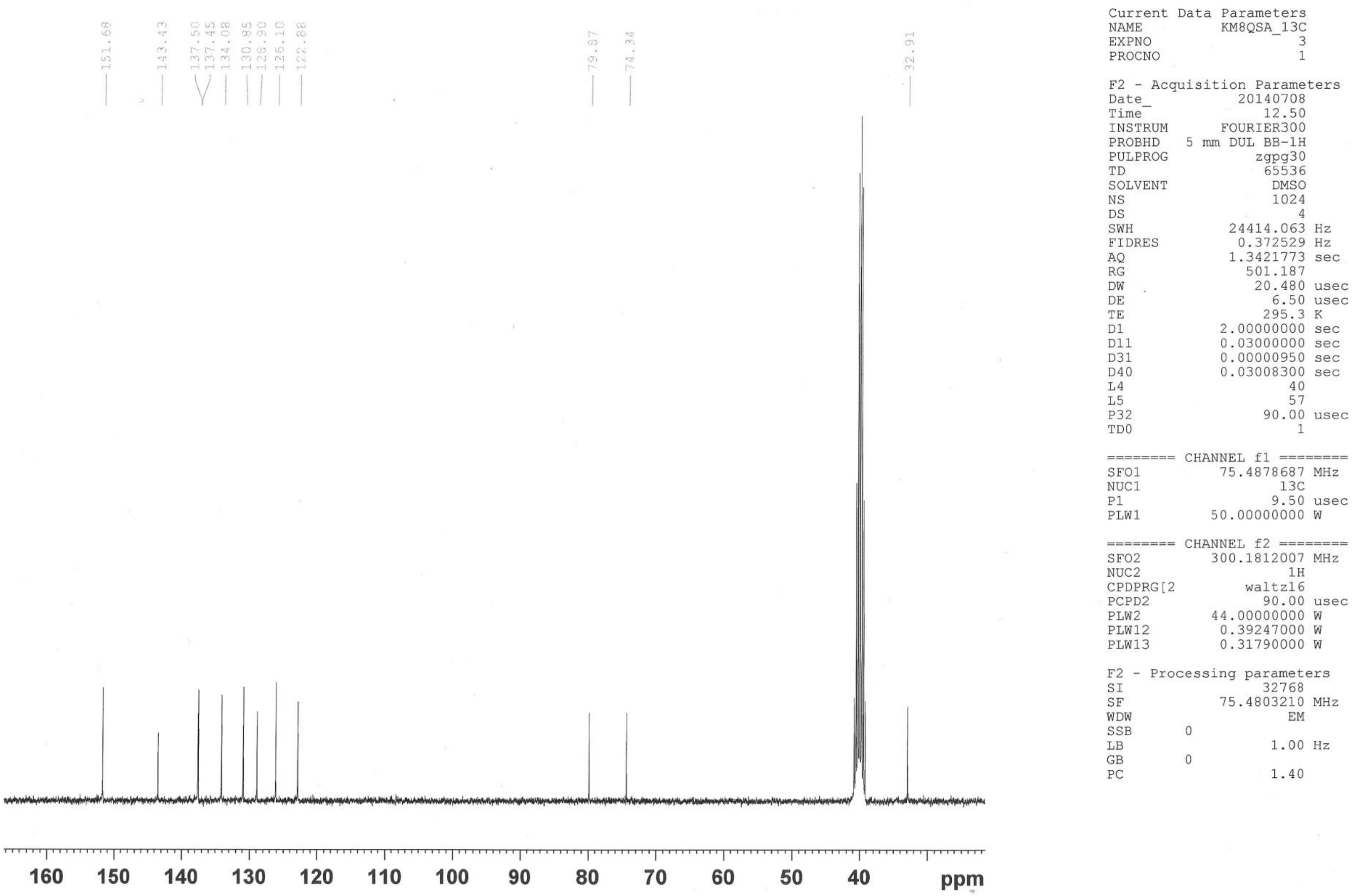

Figure S3. ^{13}C NMR spectra of 8-*N*-(prop-2-ynyl)quinolinesulfonamide (**8a**)

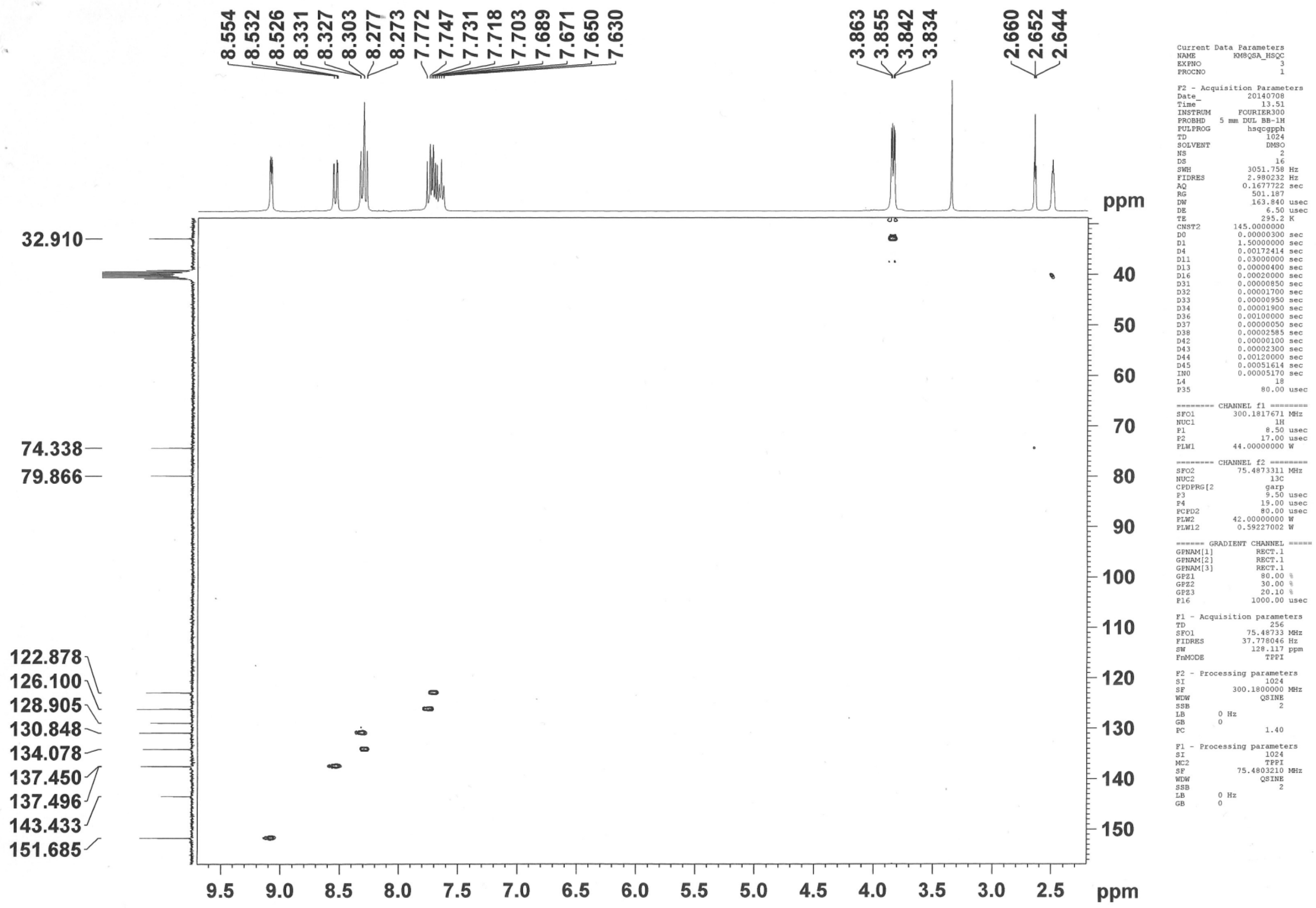

Figure S4. HSQC spectra of 8-*N*-(prop-2-ynyl)quinolinesulfonamide (**8a**)

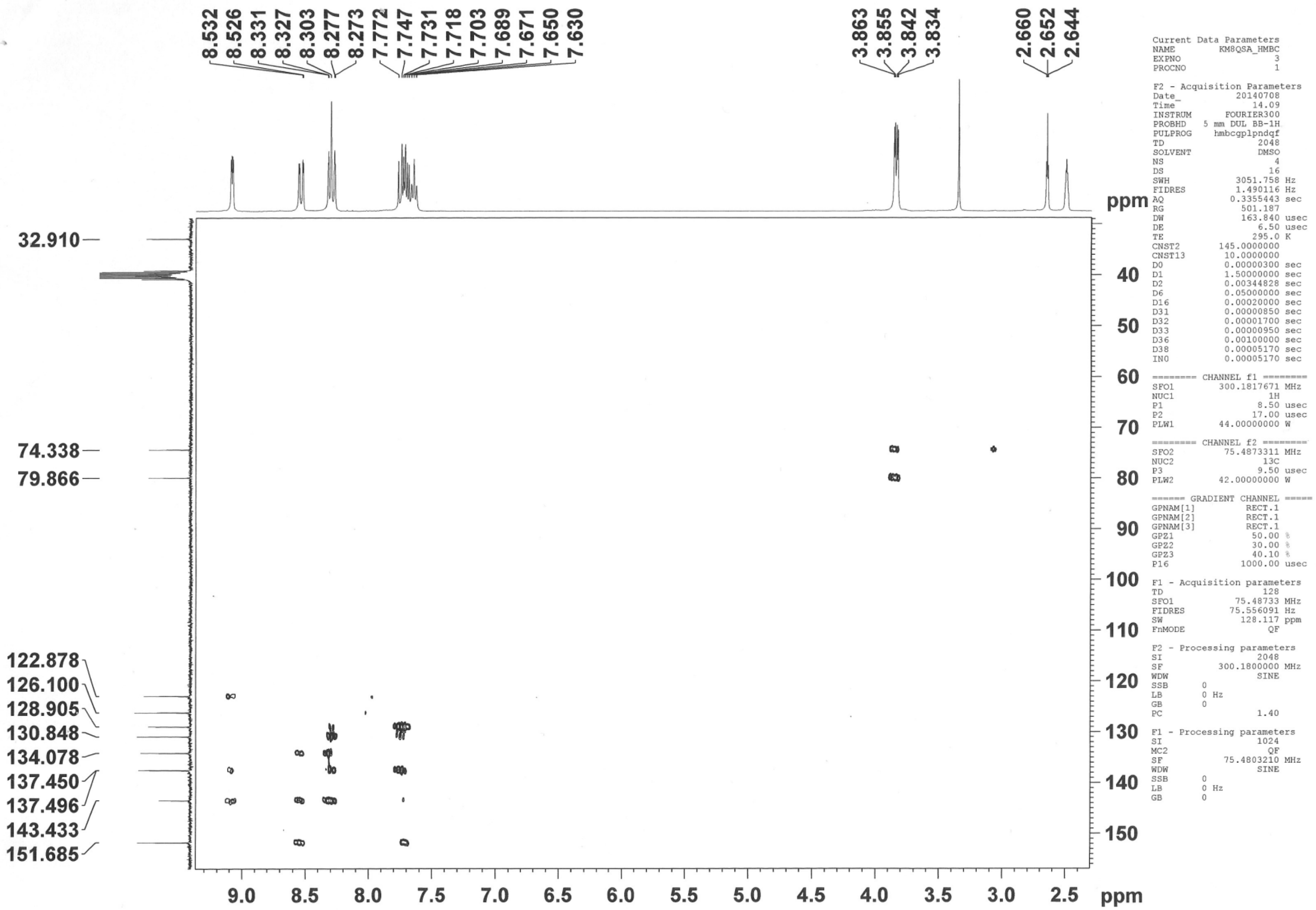

Figure S5. HMBC spectra of 8-*N*-(prop-2-ynyl)quinolinesulfonamide (**8a**)

Compound Spectrum List Report

Analysis Info

Analysis Name	D:\Data\Service_20220831\8QSAProp.d
Method	low_mass.m
Sample Name	tune_poshigh_test1
Comment	

Acquisition Date 9/13/2022 1:03:48 PM

Operator KM

Instrument impact II 1825265.10082

Acquisition Parameter

Source Type	ESI	Ion Polarity	Positive	Set Nebulizer	0.3 Bar
Focus	Active	Set Capillary	4000 V	Set Dry Heater	200 °C
Scan Begin	100 m/z	Set End Plate Offset	-500 V	Set Dry Gas	3.0 l/min
Scan End	1000 m/z	Set Charging Voltage	2000 V	Set Divert Valve	Source
		Set Corona	0 nA	Set APCI Heater	0 °C

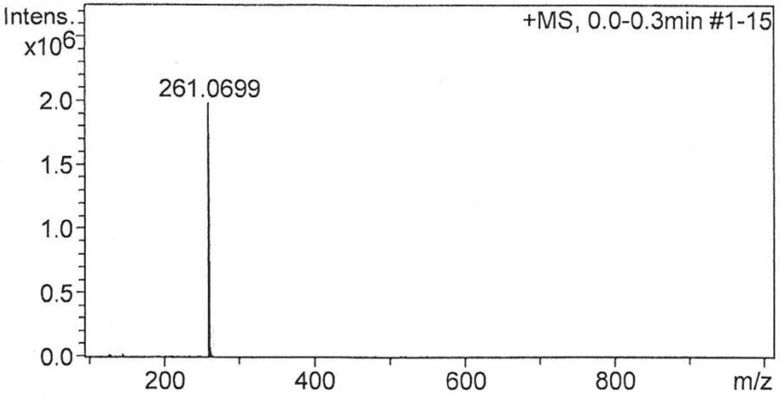

#	m/z	Res.	S/N	I	I %	FWHM
1	261.0699	32410	60509.1	1975905	100.0	0.0081

Figure S6. HR MS spectra of 8-*N*-methyl-*N*-(prop-2-ynyl)quinolinesulfonamide (**8b)**

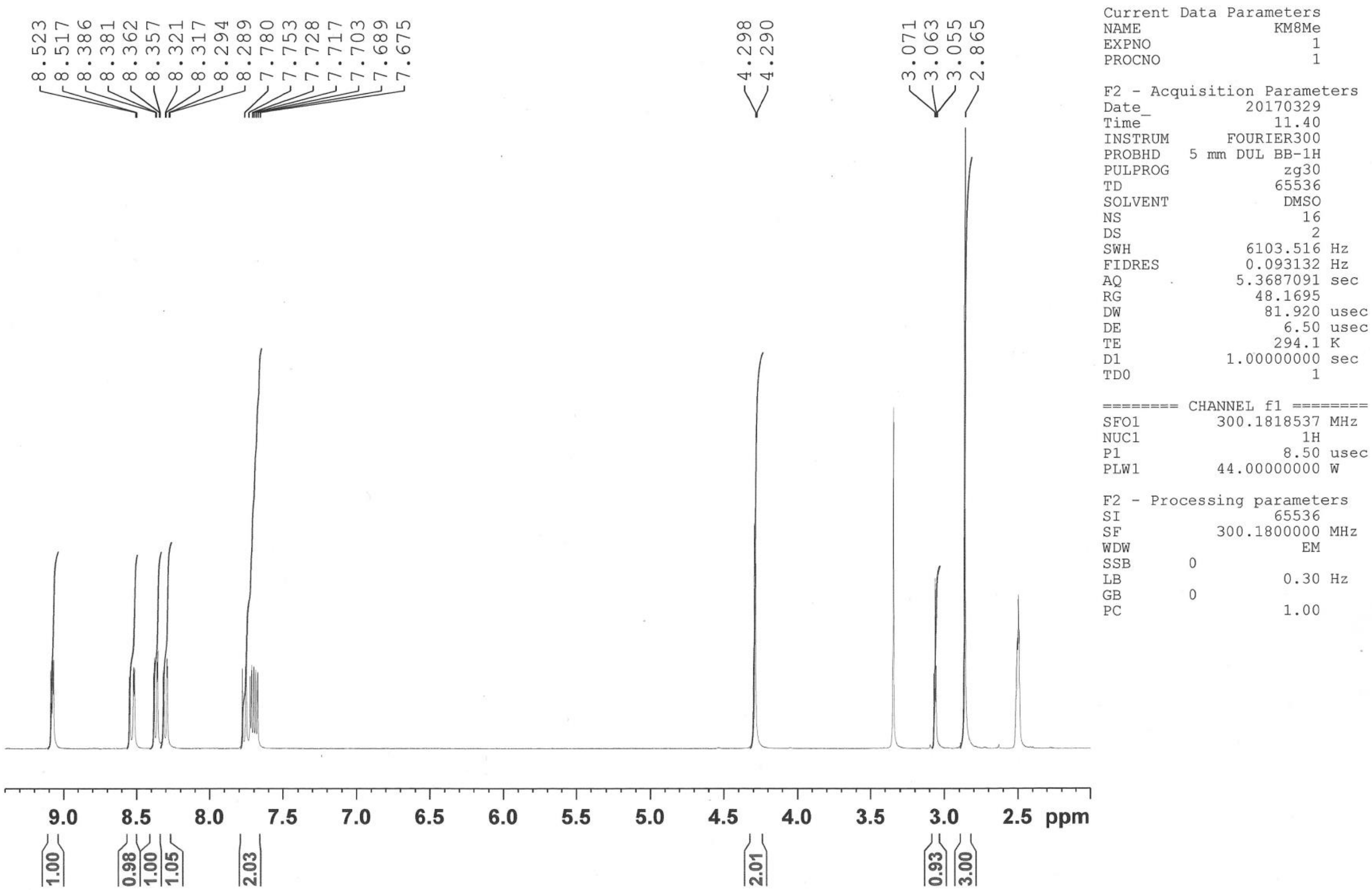

Figure S7. ^{1}H NMR spectra of 8-*N*-methyl-*N*-(prop-2-ynyl)quinolinesulfonamide (**8b)**

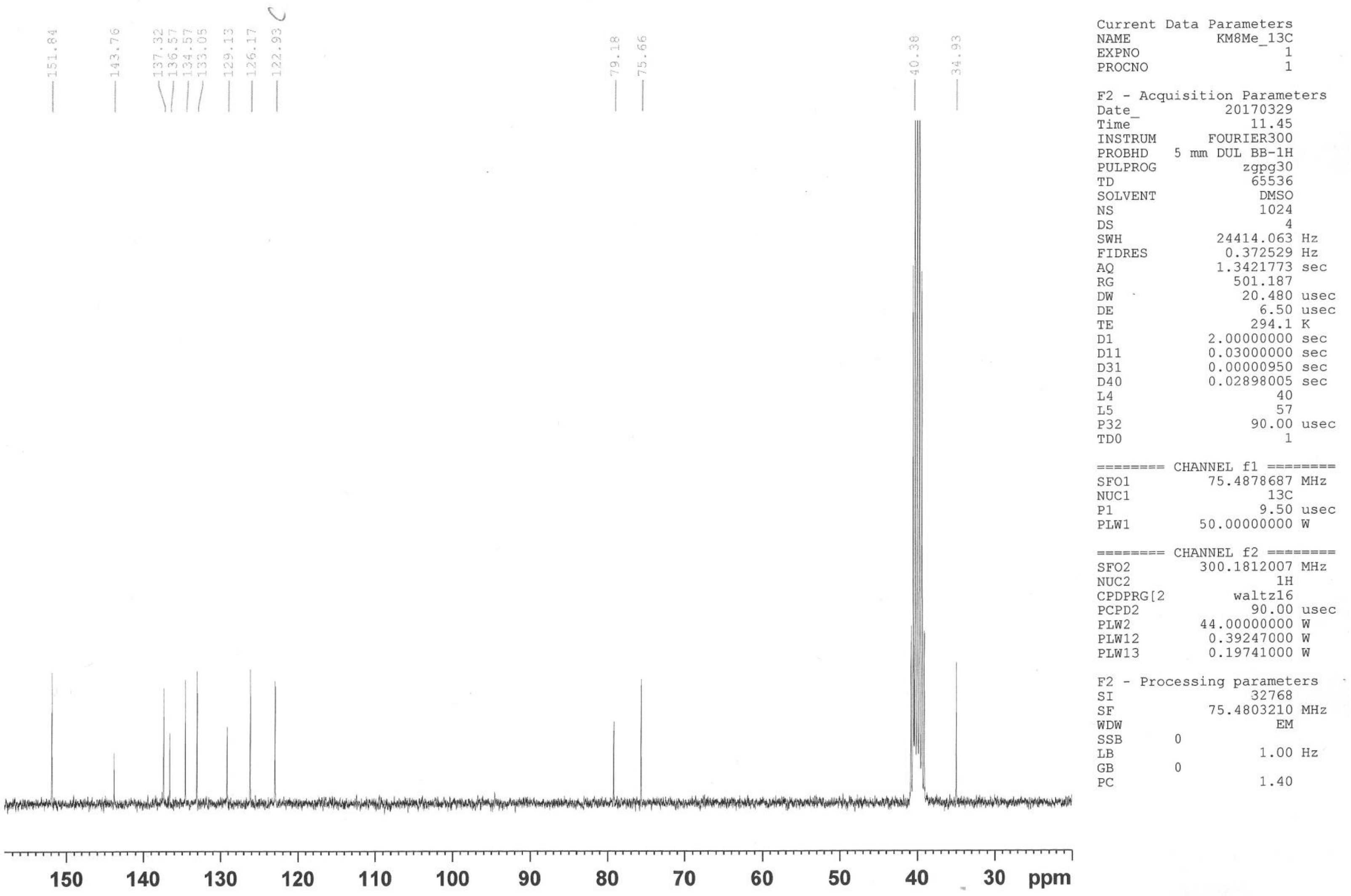

Figure S8. ^{13}C NMR spectra of 8-*N*-methyl-*N*-(prop-2-ynyl)quinolinesulfonamide (**8b**)

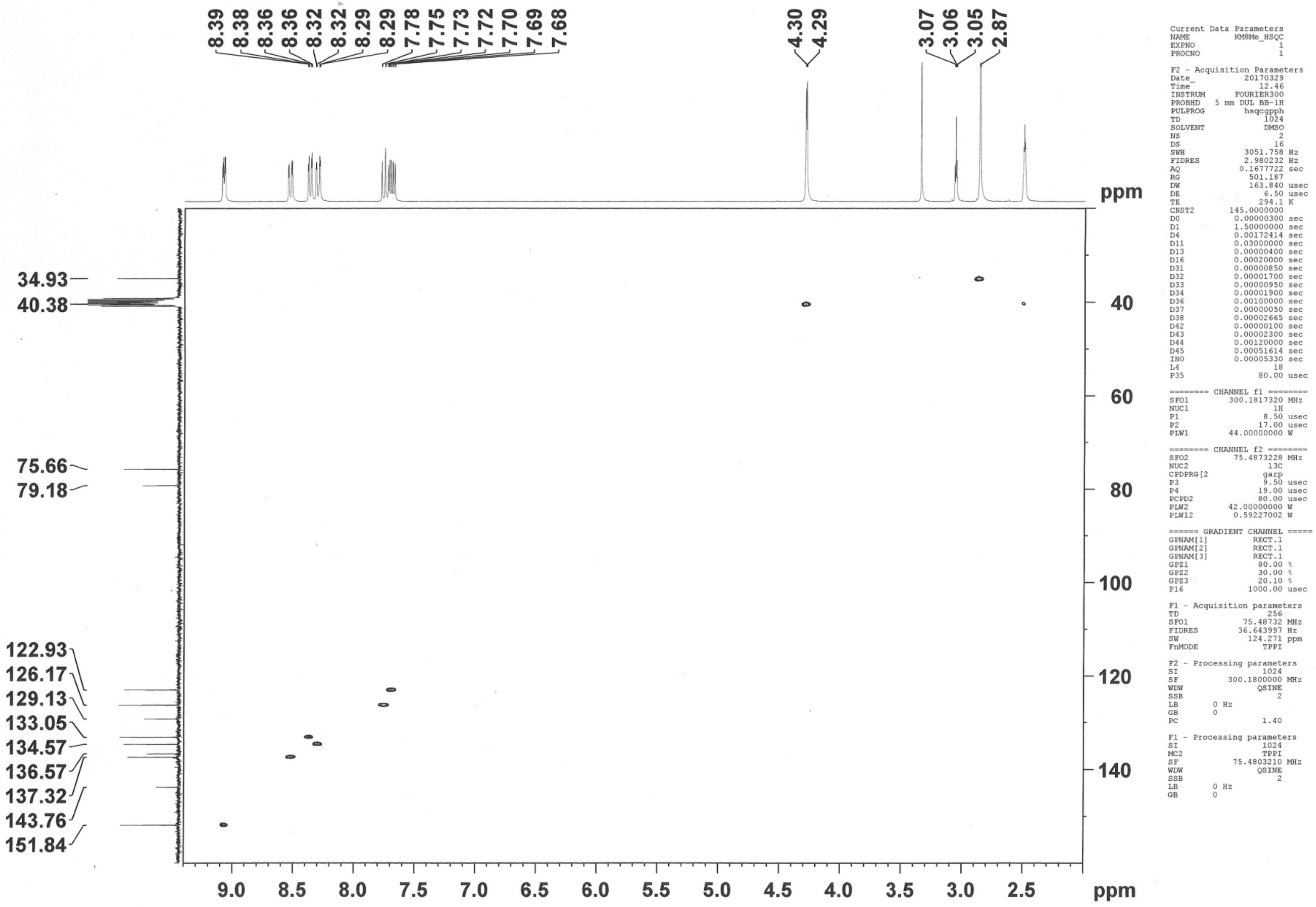

Figure S9. HSQC spectra of 8-*N*-methyl-*N*-(prop-2-ynyl)quinolinesulfonamide (**8b**)

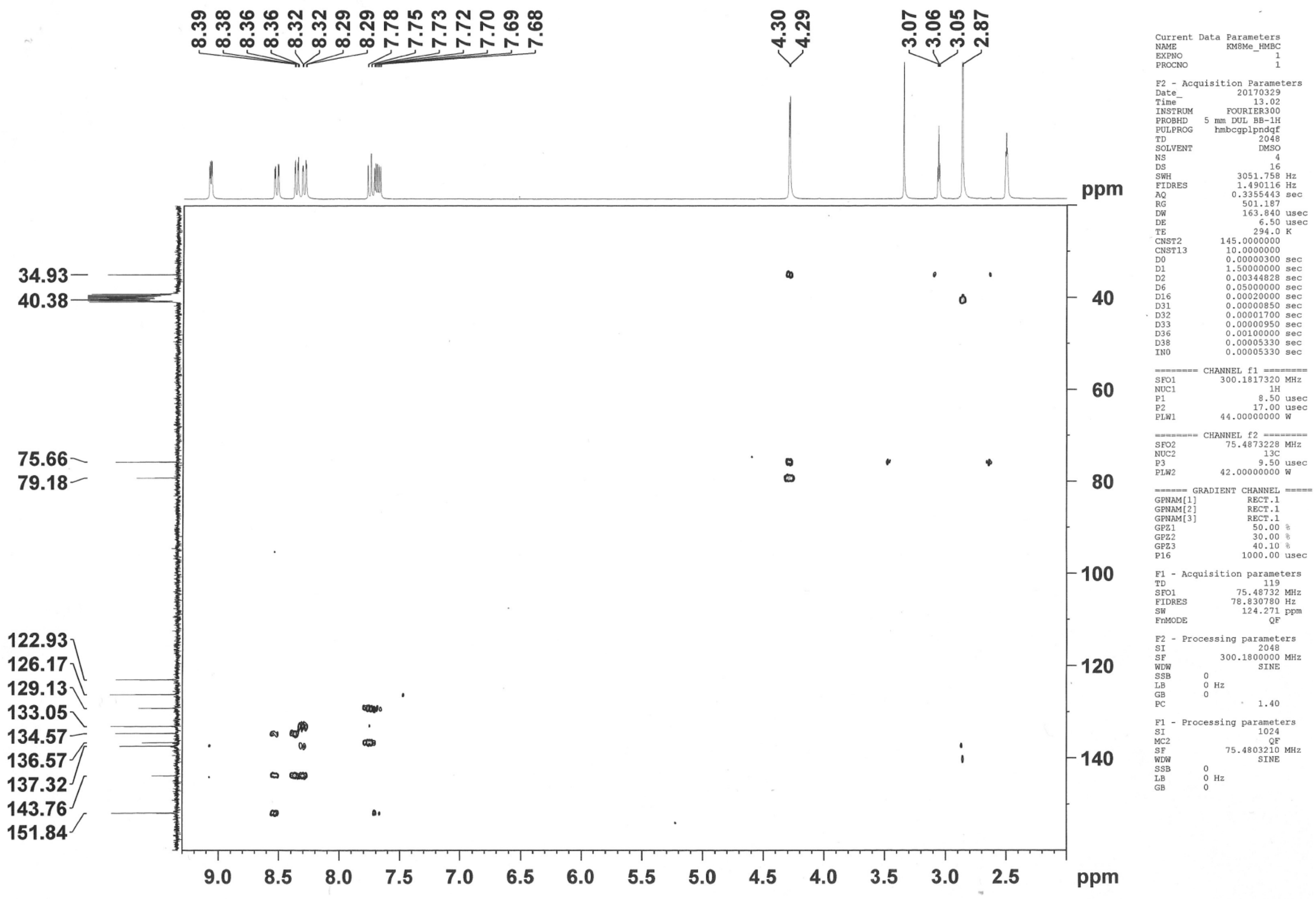

Figure S10. HMBC spectra of 8-*N*-methyl-*N*-(prop-2-ynyl)quinolinesulfonamide (**8b**)

Compound Spectrum List Report

Analysis Info

Analysis Name	D:\Data\KM7QCl.d			
Method	low_mass.m	Operator	KM	
Sample Name	TM Low concentration	Instrument	impact II	1825265.10082
Comment				

Acquisition Parameter

Source Type	ESI	Ion Polarity	Positive	Set Nebulizer	0.3 Bar
Focus	Active	Set Capillary	4000 V	Set Dry Heater	240 °C
Scan Begin	100 m/z	Set End Plate Offset	-500 V	Set Dry Gas	4.0 l/min
Scan End	1000 m/z	Set Charging Voltage	2000 V	Set Divert Valve	Source
		Set Corona	0 nA	Set APCI Heater	0 °C

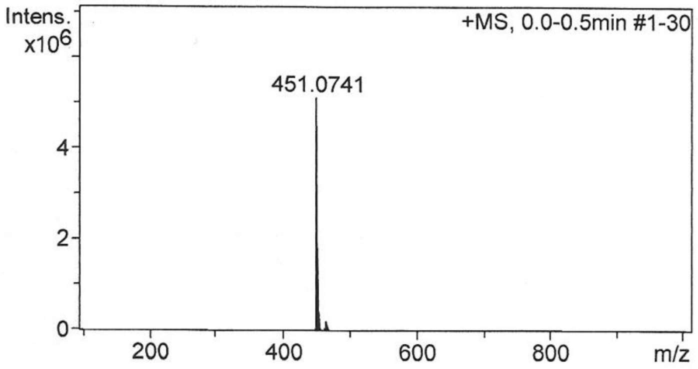

#	m/z	Res.	S/N	I	I %	FWHM
1	451.0741	46889	14570.0	5115211	100.0	0.0096
2	453.0711	42091	5173.1	1821962	35.6	0.0108
3	473.0557	37315	1641.0	630401	12.3	0.0127
4	475.0531	31446	613.5	237045	4.6	0.0151

Figure S11. HR MS spectra of 8-*N*-{[1-(7-chloroquinolin-4-yl)-1*H*-1,2,3-triazol-4-yl]methyl}quinolinesulfonamide (**9a**)

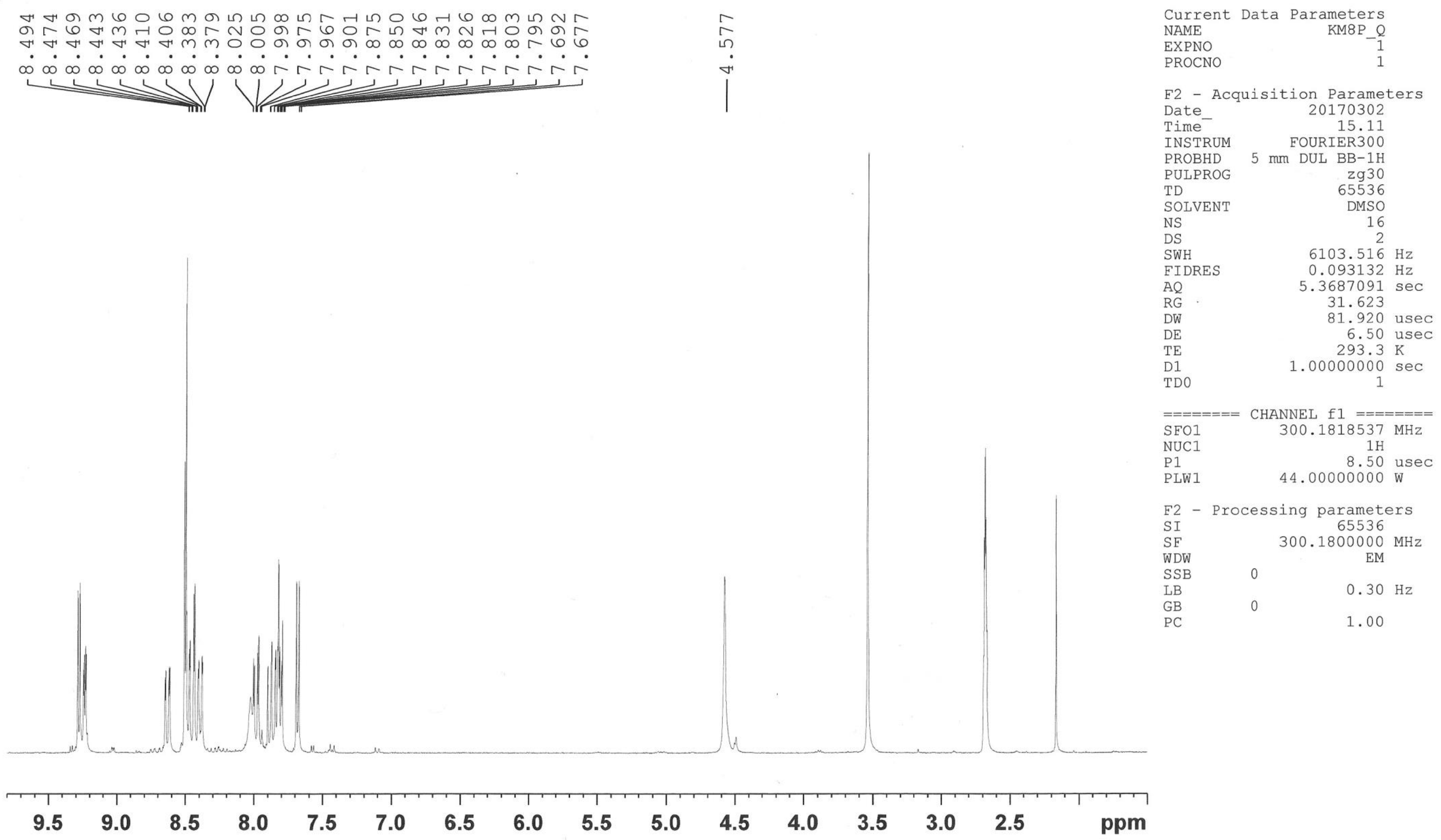

Figure S12. ^{1}H NMR spectra of 8-*N*-{[1-(7-chloroquinolin-4-yl)-1*H*-1,2,3-triazol-4-yl]methyl}quinolinesulfonamide (**9a**)

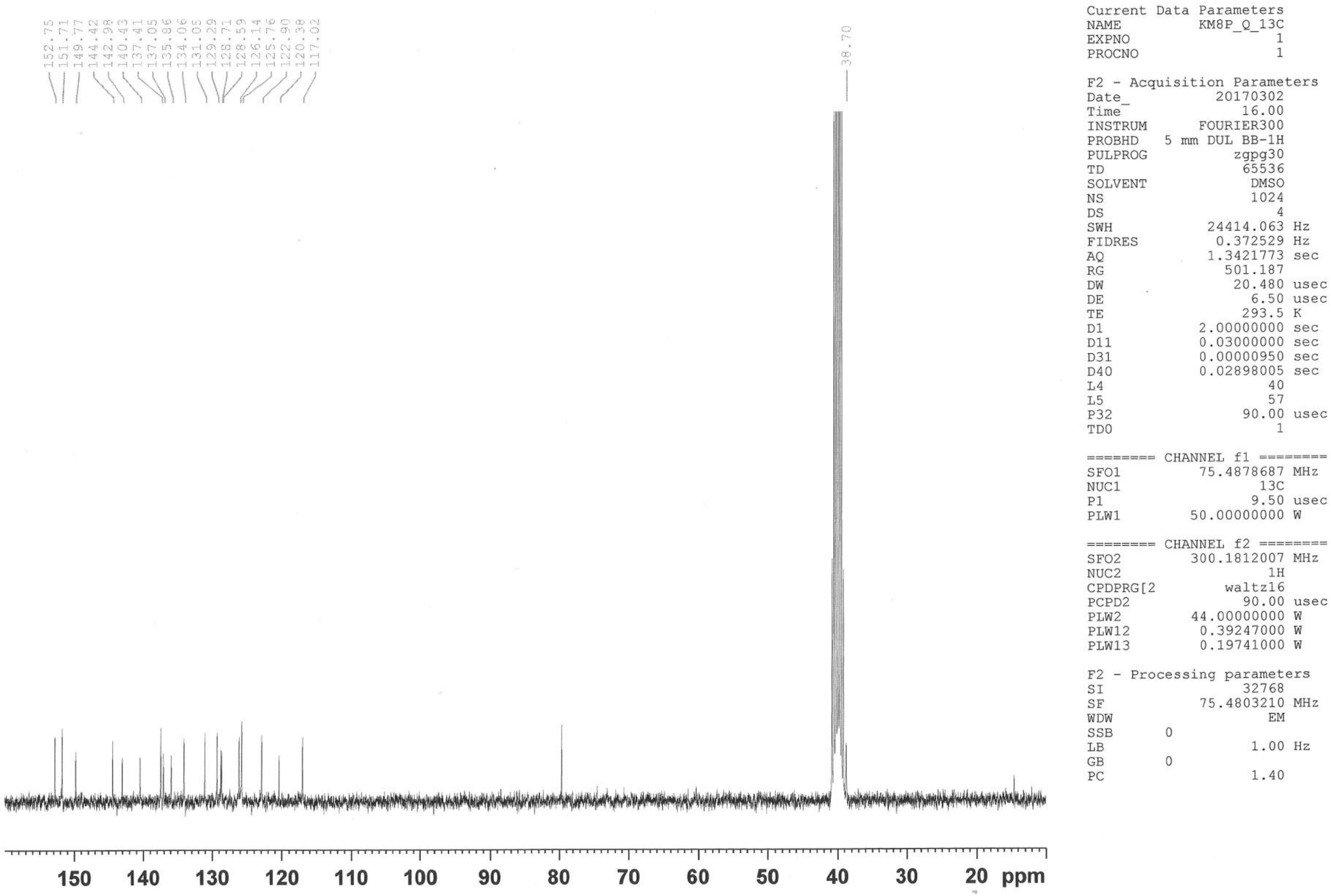

Figure S13. ^{13}C NMR spectra of 8-*N*-{[1-(7-chloroquinolin-4-yl)-1*H*-1,2,3-triazol-4-yl]methyl}quinolinesulfonamide (**9a**)

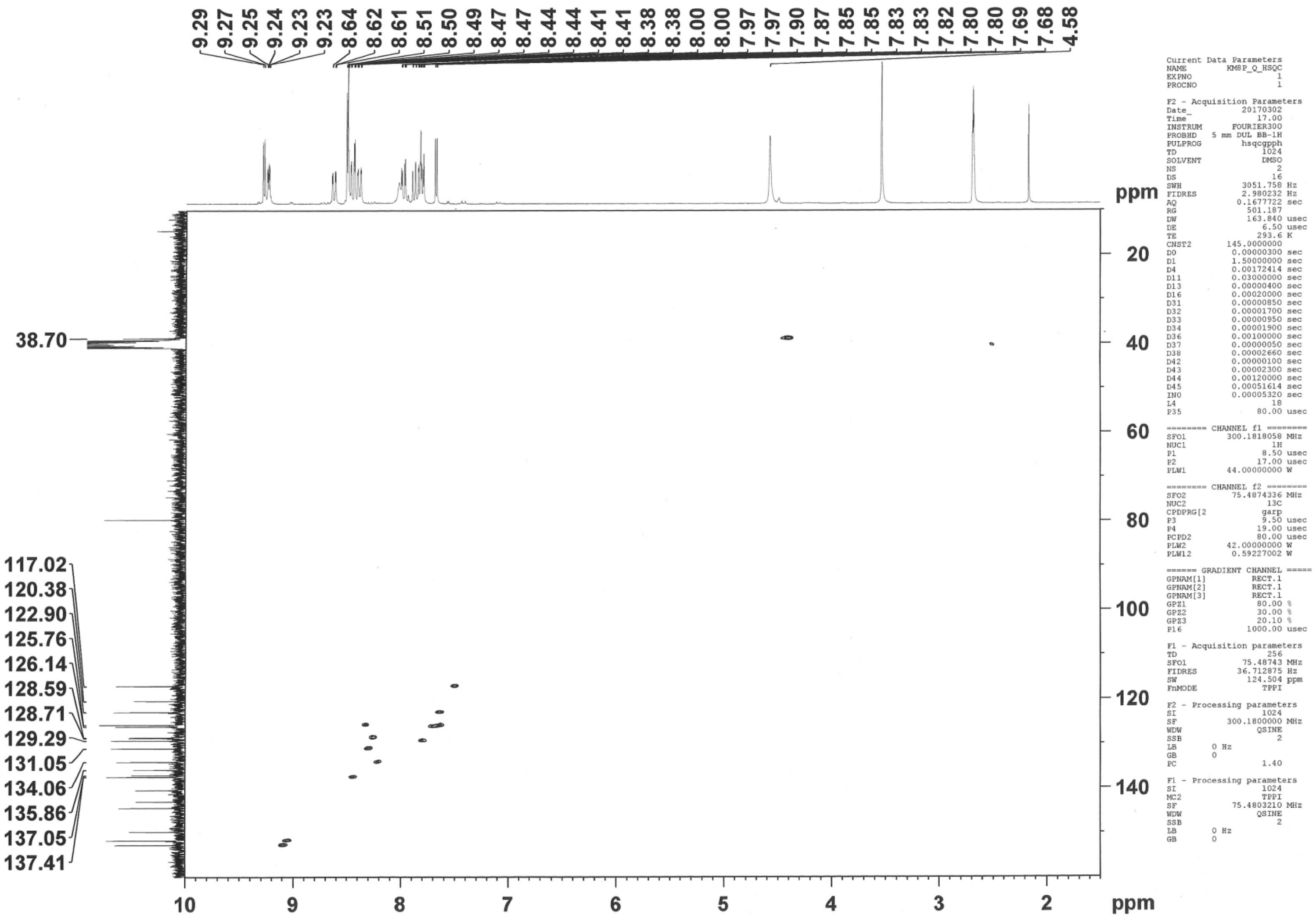

Figure S14. HSQC spectra of 8-*N*-{[1-(7-chloroquinolin-4-yl)-1*H*-1,2,3-triazol-4-yl]methyl}quinolinesulfonamide (**9a**)

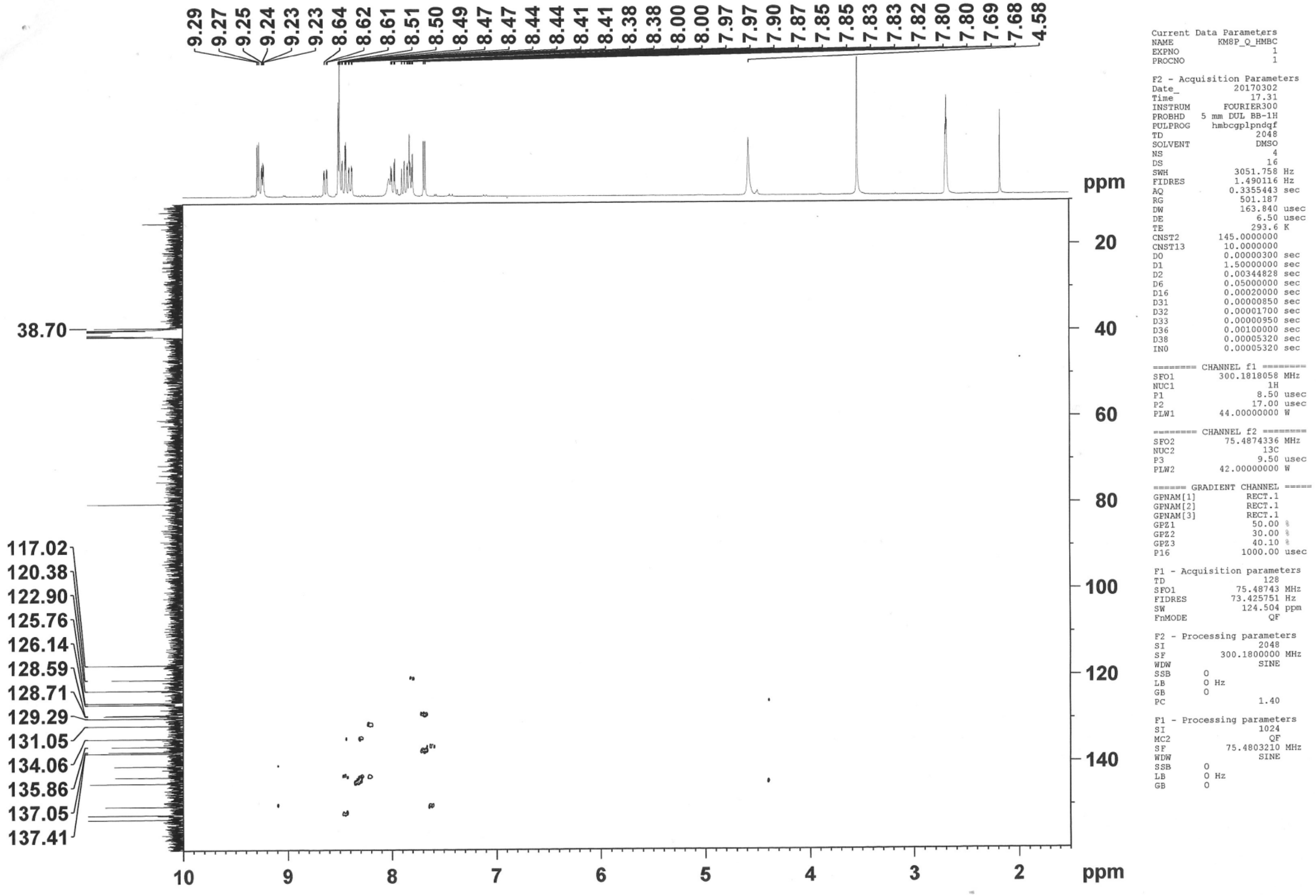

Figure S15. HMBC spectra of 8-*N*-{[1-(7-chloroquinolin-4-yl)-1*H*-1,2,3-triazol-4-yl]methyl}quinolinesulfonamide (**9a**)

Compound Spectrum List Report

Analysis Info

Analysis Name	D:\Data\KMFosf.d			
Method	low_mass.m	Operator	KM	
Sample Name	TM Low concentration	Instrument	impact II	1825265.10082
Comment				

Acquisition Parameter

Source Type	ESI	Ion Polarity	Positive	Set Nebulizer	0.3 Bar
Focus	Active	Set Capillary	4000 V	Set Dry Heater	240 °C
Scan Begin	100 m/z	Set End Plate Offset	-500 V	Set Dry Gas	4.0 l/min
Scan End	1000 m/z	Set Charging Voltage	2000 V	Set Divert Valve	Source
		Set Corona	0 nA	Set APCI Heater	0 °C

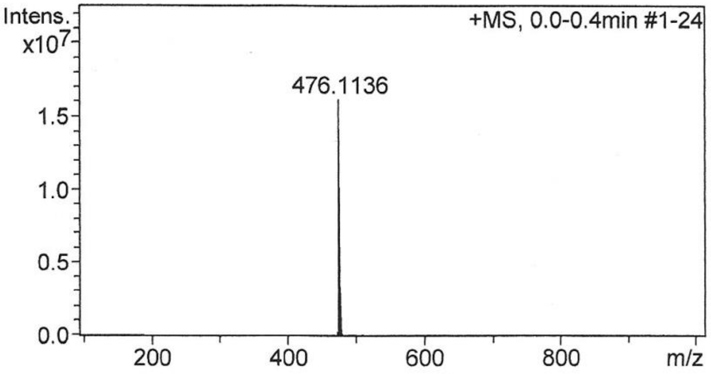

#	m/z	Res.	S/N	I	I %	FWHM
1	476.1136	48479	36474.8	16198640	100.0	0.0098

Figure S16. HR MS spectra of diethyl 2-{4-[methyl-(8-sulfamoylquinolyl)-1*H*-1,2,3-triazol-1-yl]}ethylphosphonate (**9b**)

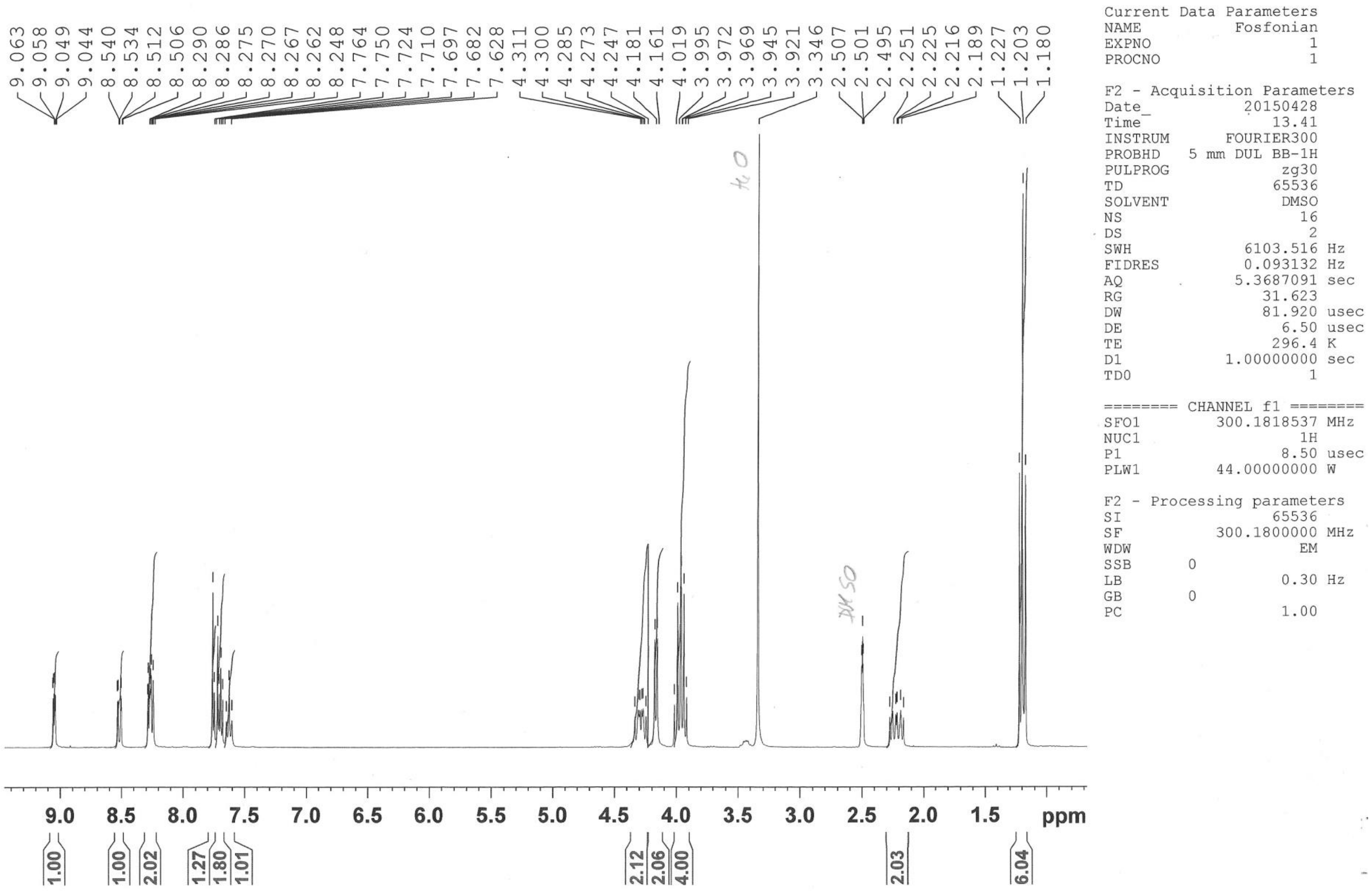

Figure S17. ^{1}H NMR spectra of diethyl 2-{4-[methyl-(8-sulfamoylquinolyl)-1*H*-1,2,3-triazol-1-yl]}ethylphosphonate (**9b**)

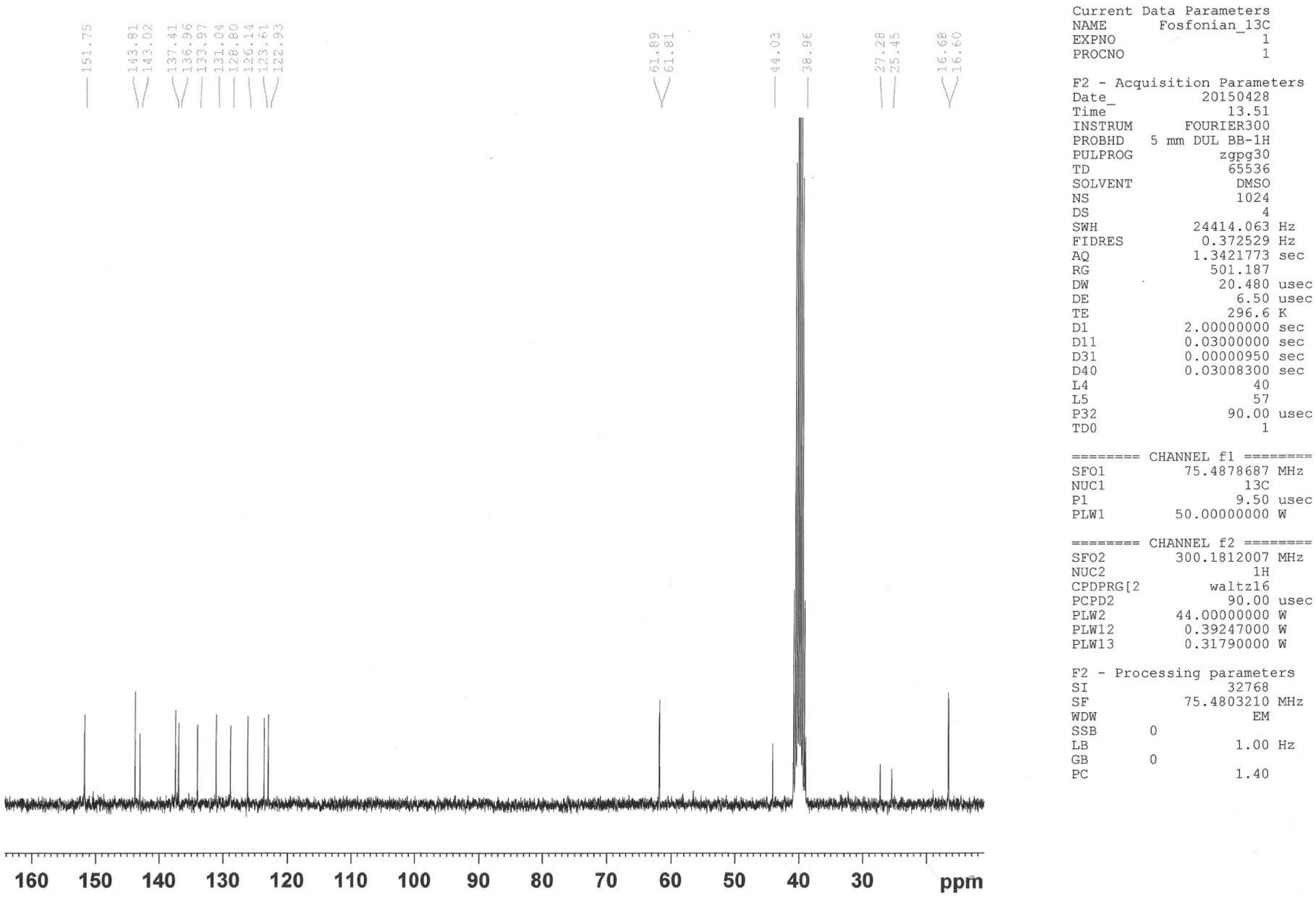

Figure S18. ^{13}C NMR spectra of diethyl 2-{4-[methyl-(8-sulfamoylquinolyl)-1*H*-1,2,3-triazol-1-yl]}ethylphosphonate (**9b**)

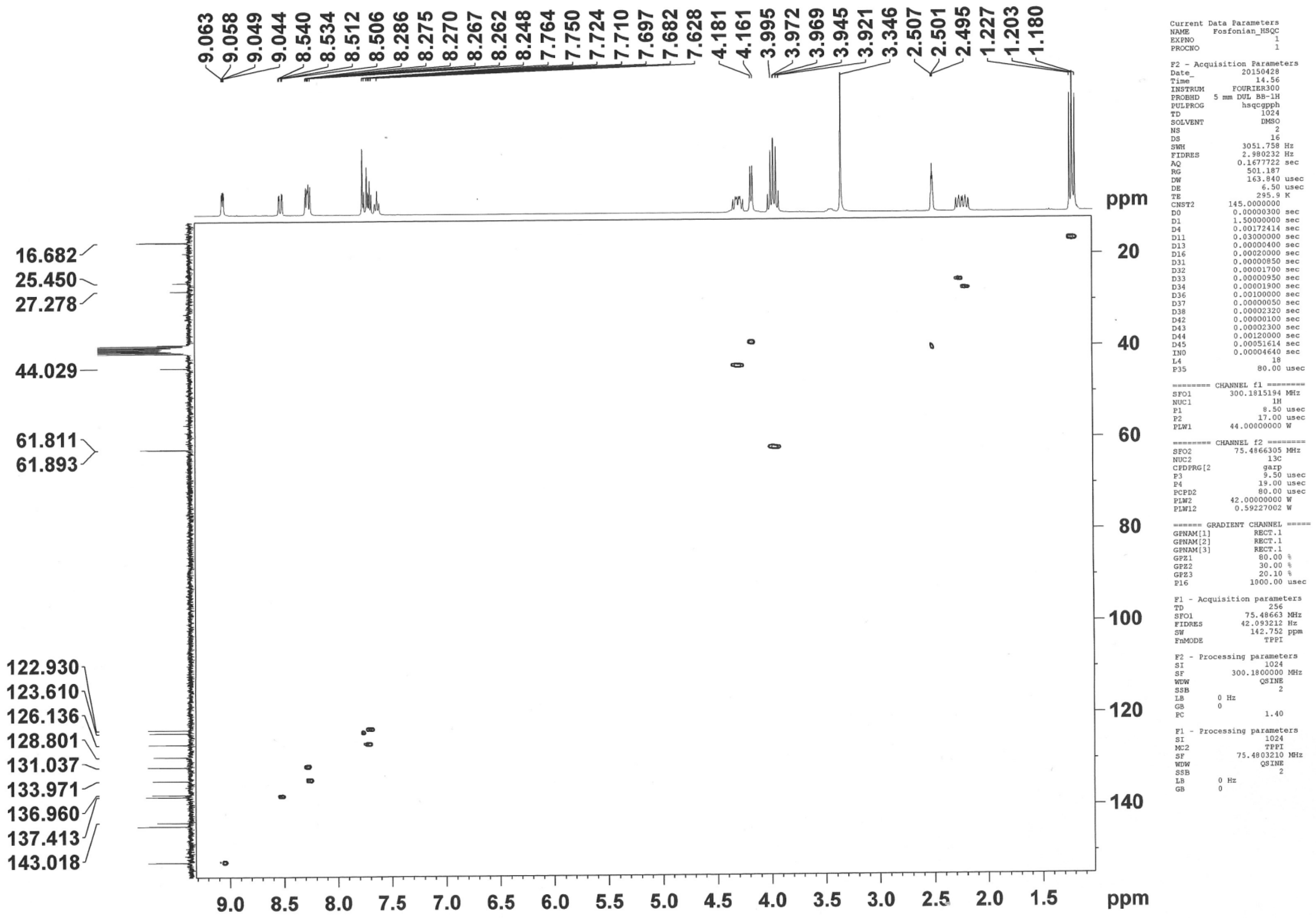

Figure S19. HSQC spectra diethyl 2-{4-[methyl-(8-sulfamoylquinolyl)-1*H*-1,2,3-triazol-1-yl]}ethylphosphonate (**9b**)

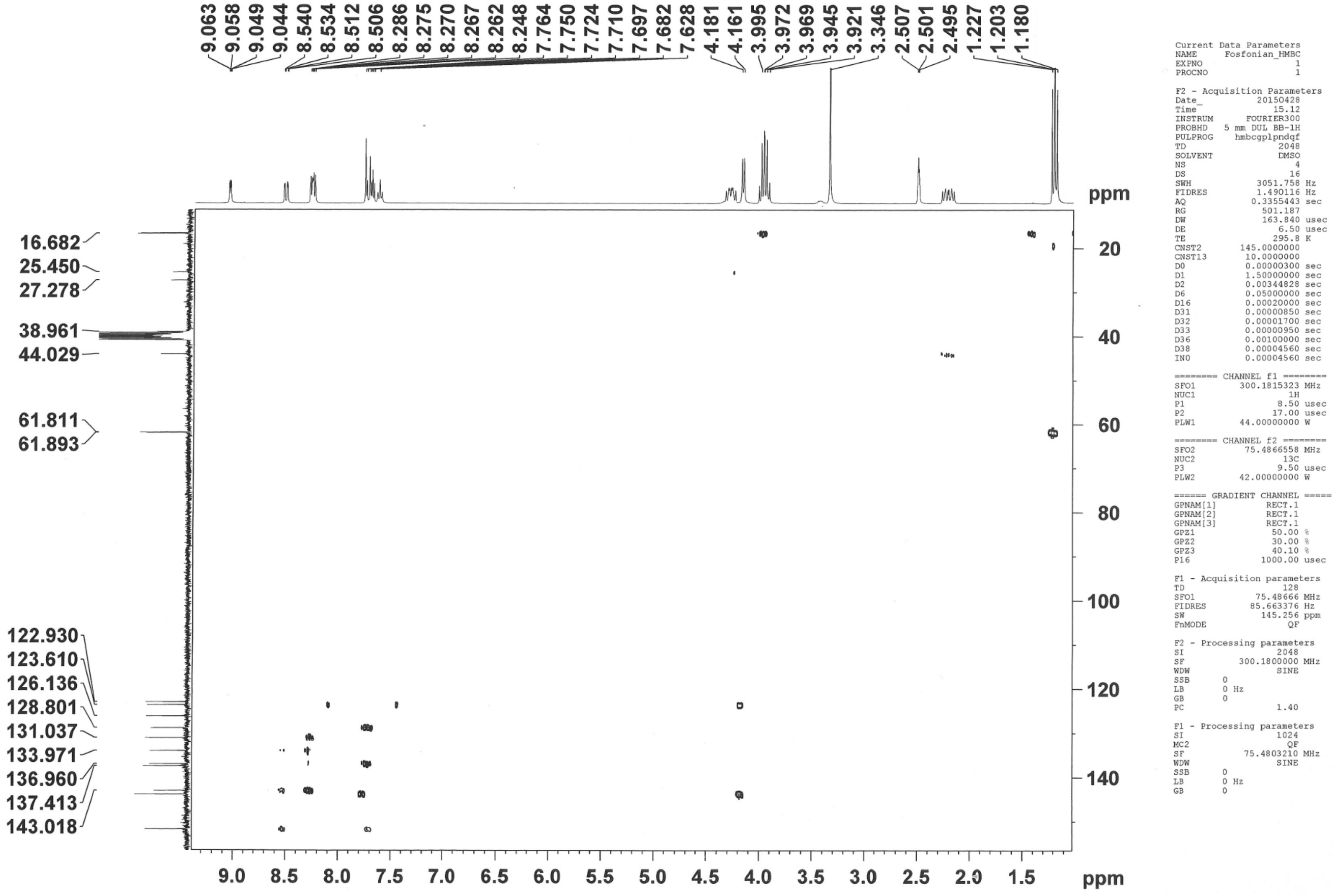

Figure S20. HMBC spectra diethyl 2-{4-[methyl-(8-sulfamoylquinolyl)-1*H*-1,2,3-triazol-1-yl]}ethylphosphonate (**9b**)

Compound Spectrum List Report

Analysis Info

Analysis Name	D:\Data\KMEster.d			
Method	low_mass.m	Operator	KM	
Sample Name	TM Low concentration	Instrument	impact II	1825265.10082
Comment				

Acquisition Parameter

Source Type	ESI	Ion Polarity	Positive	Set Nebulizer	0.3 Bar
Focus	Active	Set Capillary	4000 V	Set Dry Heater	240 °C
Scan Begin	100 m/z	Set End Plate Offset	-500 V	Set Dry Gas	4.0 l/min
Scan End	1000 m/z	Set Charging Voltage	2000 V	Set Divert Valve	Source
		Set Corona	0 nA	Set APCI Heater	0 °C

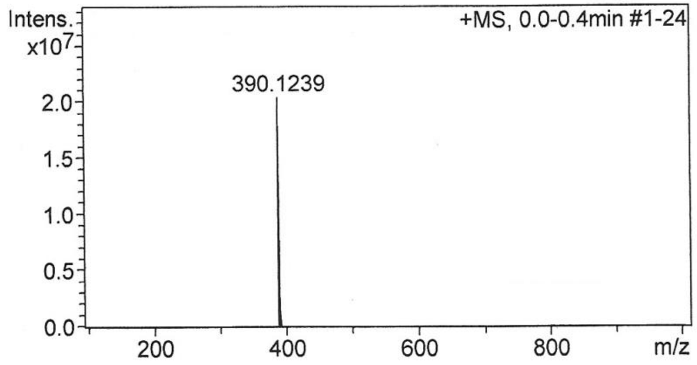

#	m/z	Res.	S/N	I	I %	FWHM
1	390.1239	22292	75359.1	20377458	100.0	0.0175

Figure S21 . HR MS spectra of ethyl 3-{[4-(8-sulfamoylquinolyl)methyl]-1*H*-1,2,3-triazol-1-yl}propanoate (**9c**)

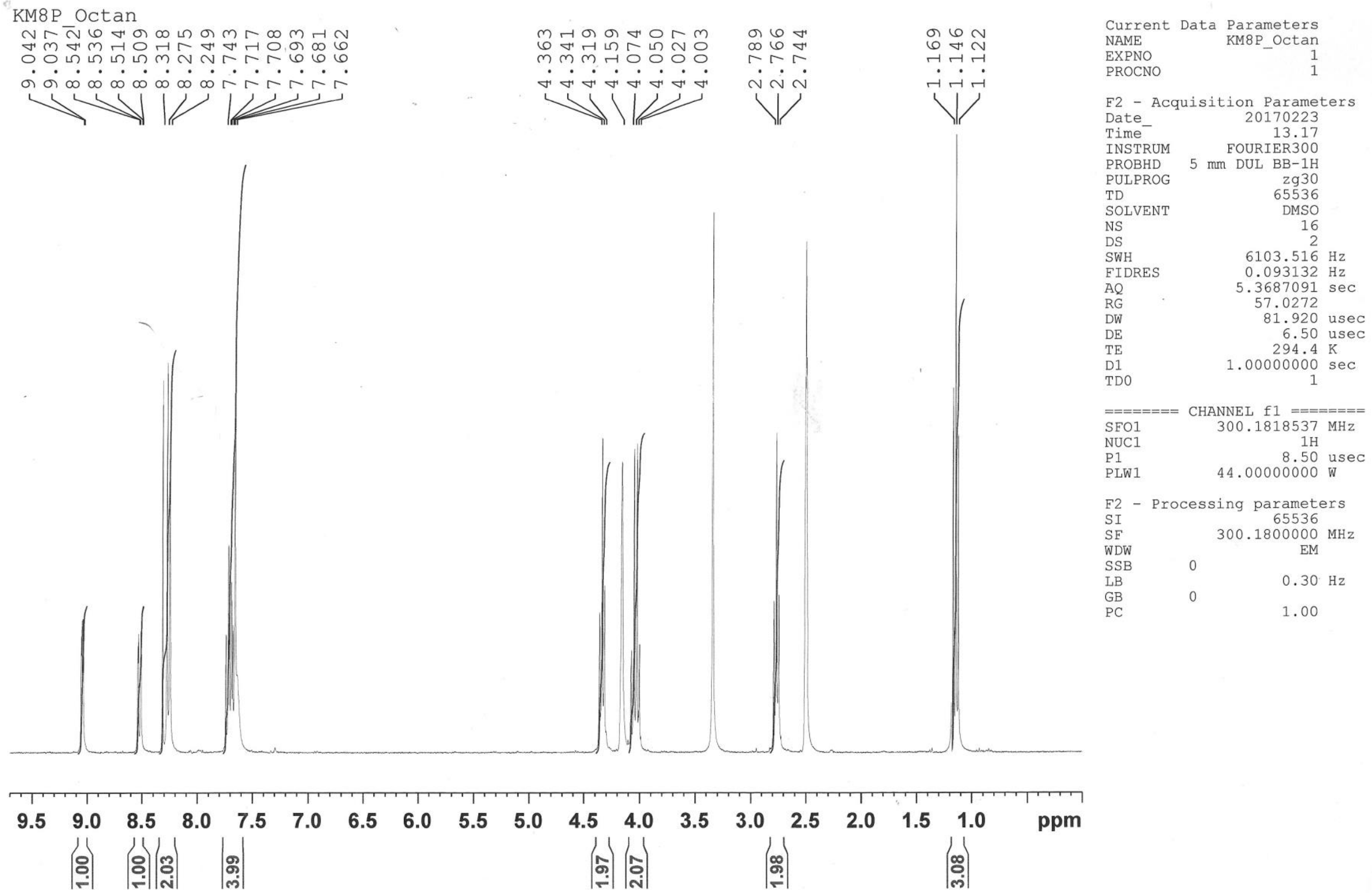

Figure S22 . ^{1}H NMR spectra of ethyl 3-{[4-(8-sulfamoylquinolyl)methyl]-1*H*-1,2,3-triazol-1-yl}propanoate (**9c**)

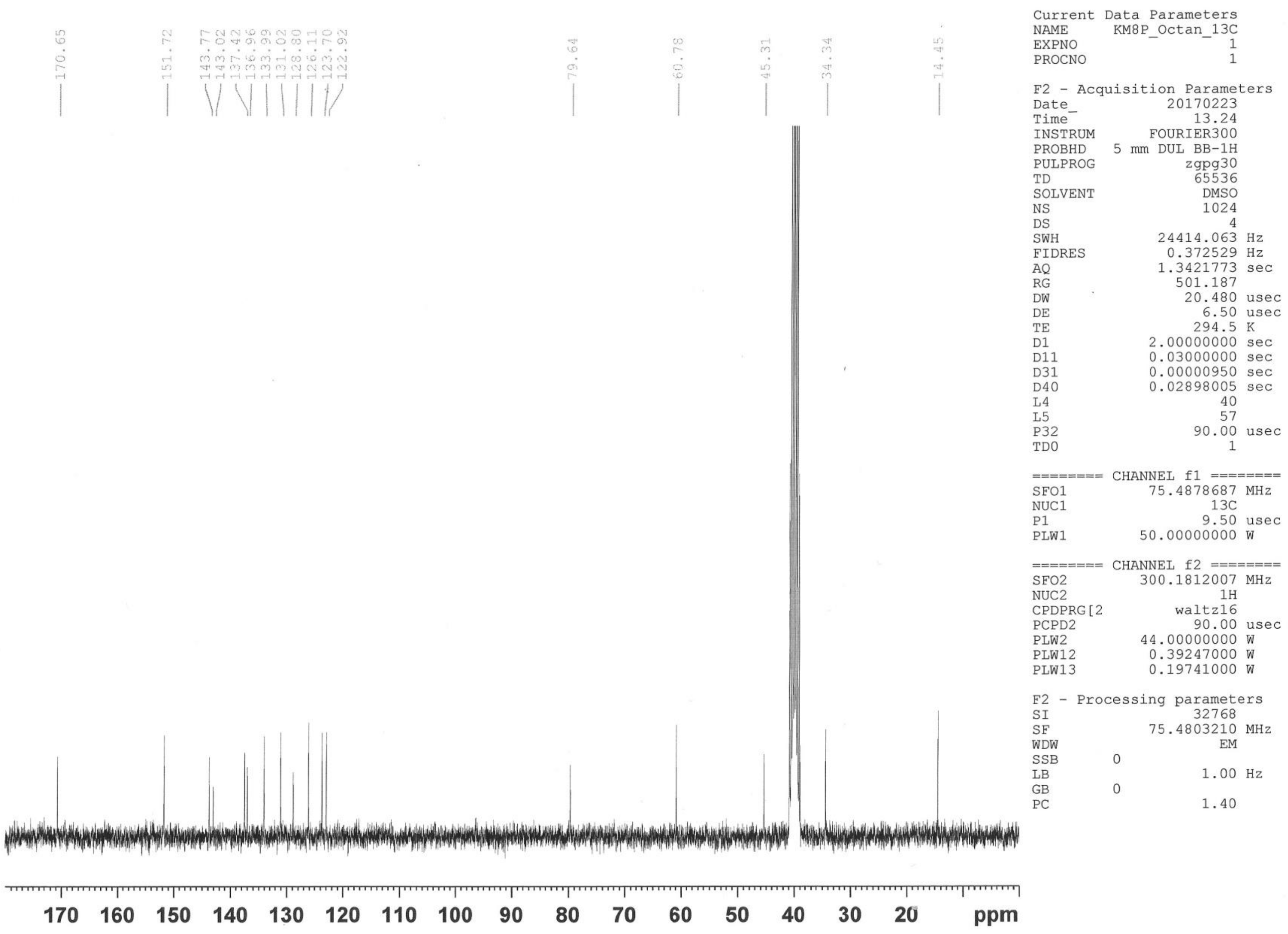

Figure S23 . ^{13}C NMR spectra of ethyl 3-{[4-(8-sulfamoylquinolyl)methyl]-1*H*-1,2,3-triazol-1-yl}propanoate (**9c**)

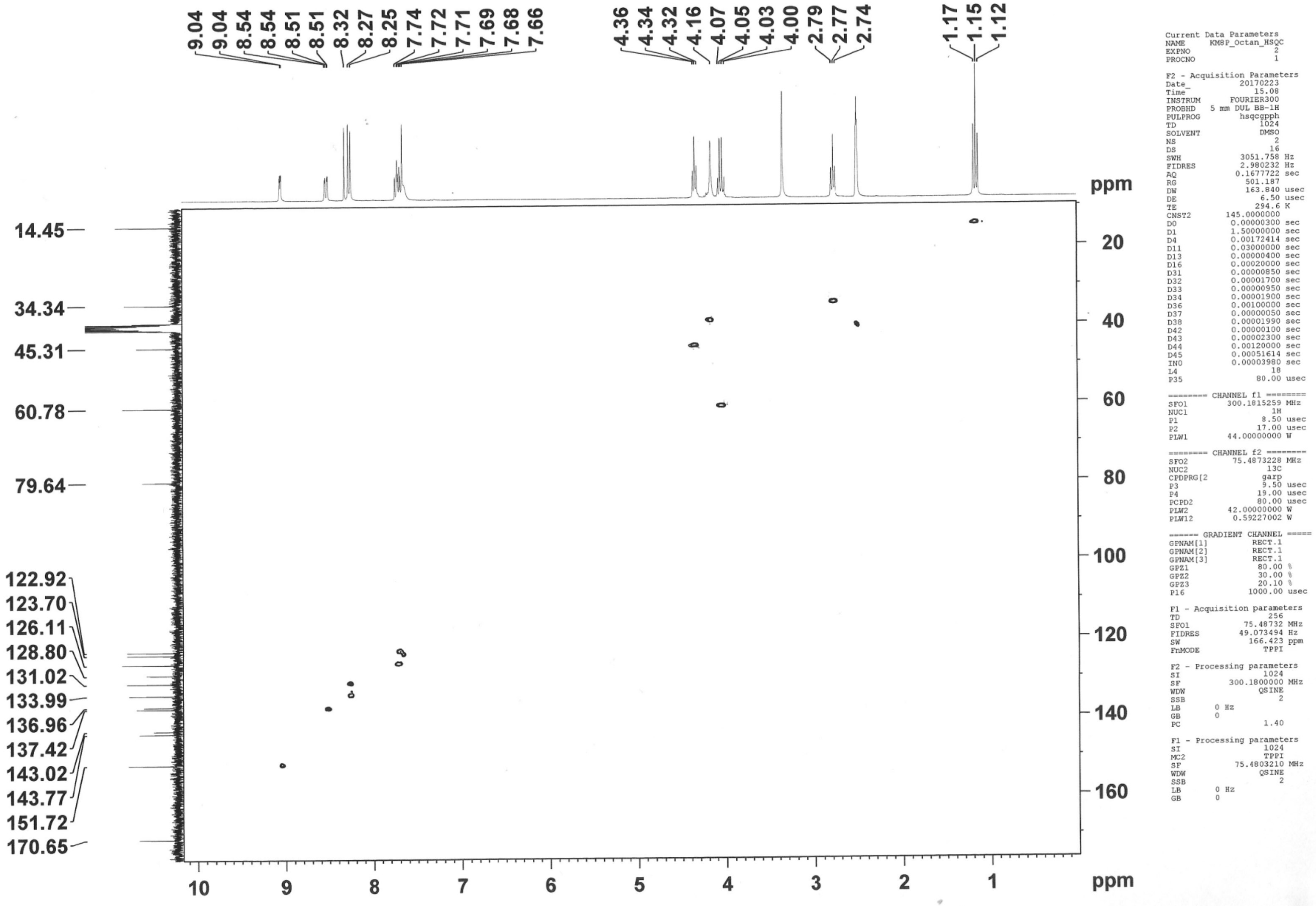

Figure S24 . [1]HSQC spectra of ethyl 3-{[4-(8-sulfamoylquinolyl)methyl]-1*H*-1,2,3-triazol-1-yl}propanoate (**9c**)

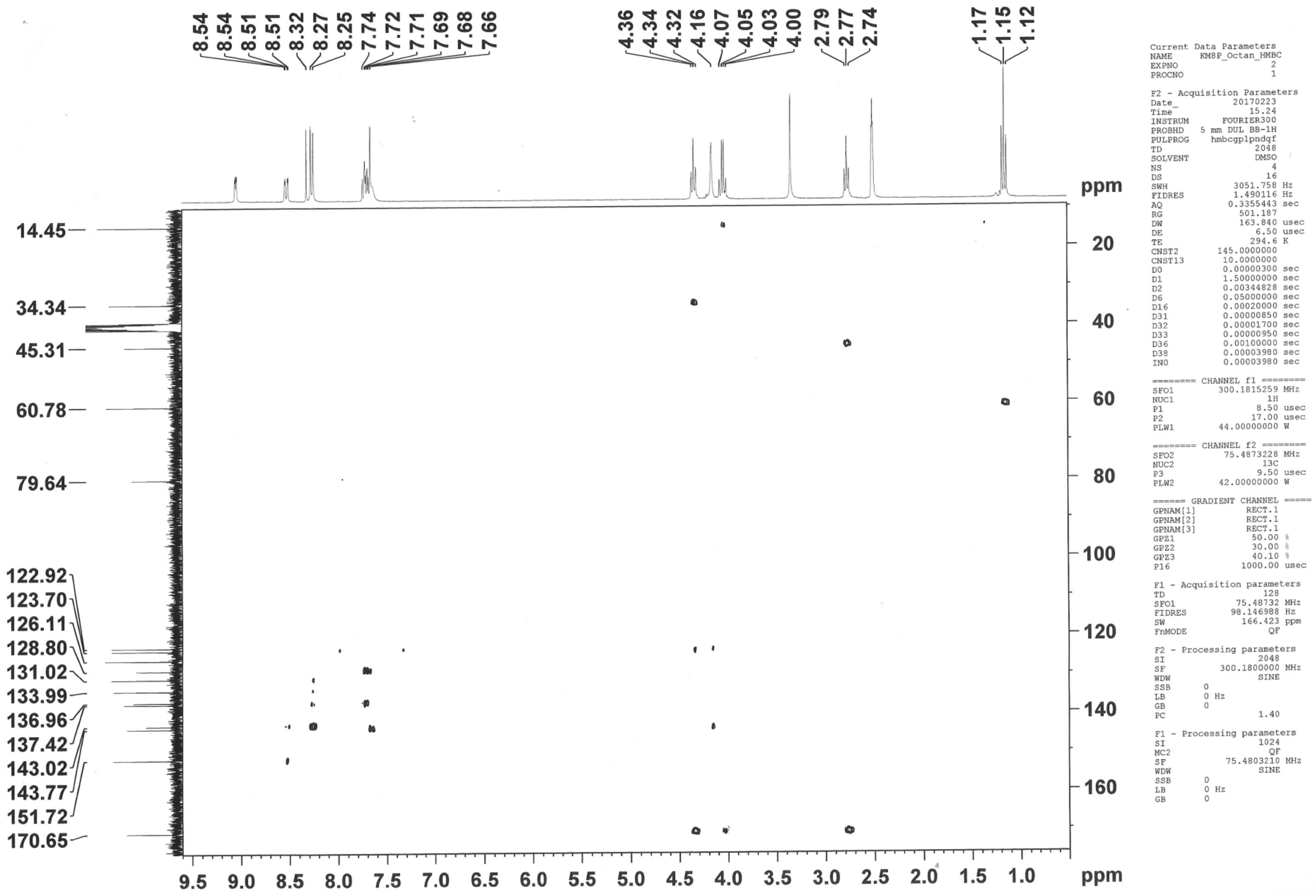

Figure S25 . [1]HMBC spectra of ethyl 3-{[4-(8-sulfamoylquinolyl)methyl]-1*H*-1,2,3-triazol-1-yl}propanoate (**9c**)

Compound Spectrum List Report

Analysis Info

Analysis Name	D:\Data\KMBuyl.d			
Method	low_mass.m	Operator	KM	
Sample Name	TM Low concentration	Instrument	impact II	1825265.10082
Comment				

Acquisition Parameter

Source Type	ESI	Ion Polarity	Positive	Set Nebulizer	0.3 Bar
Focus	Active	Set Capillary	4000 V	Set Dry Heater	240 °C
Scan Begin	100 m/z	Set End Plate Offset	-500 V	Set Dry Gas	4.0 l/min
Scan End	1000 m/z	Set Charging Voltage	2000 V	Set Divert Valve	Source
		Set Corona	0 nA	Set APCI Heater	0 °C

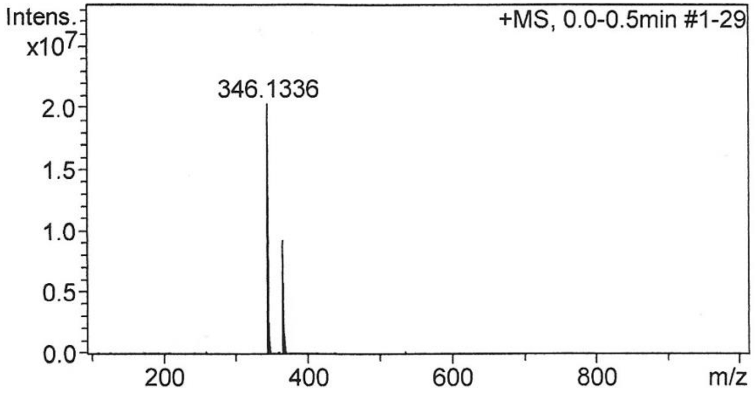

#	m/z	Res.	S/N	I	I %	FWHM
1	346.1336	20957	86756.1	20377468	100.0	0.0165
2	368.1154	43608	36143.5	9331787	45.8	0.0084

Figure S26 . HR MS spectra of 8-*N*-{[1-(1-butyl)-1*H*-1,2,3-triazol-4-yl]methyl}quinolinesulfonamide (**9d**)

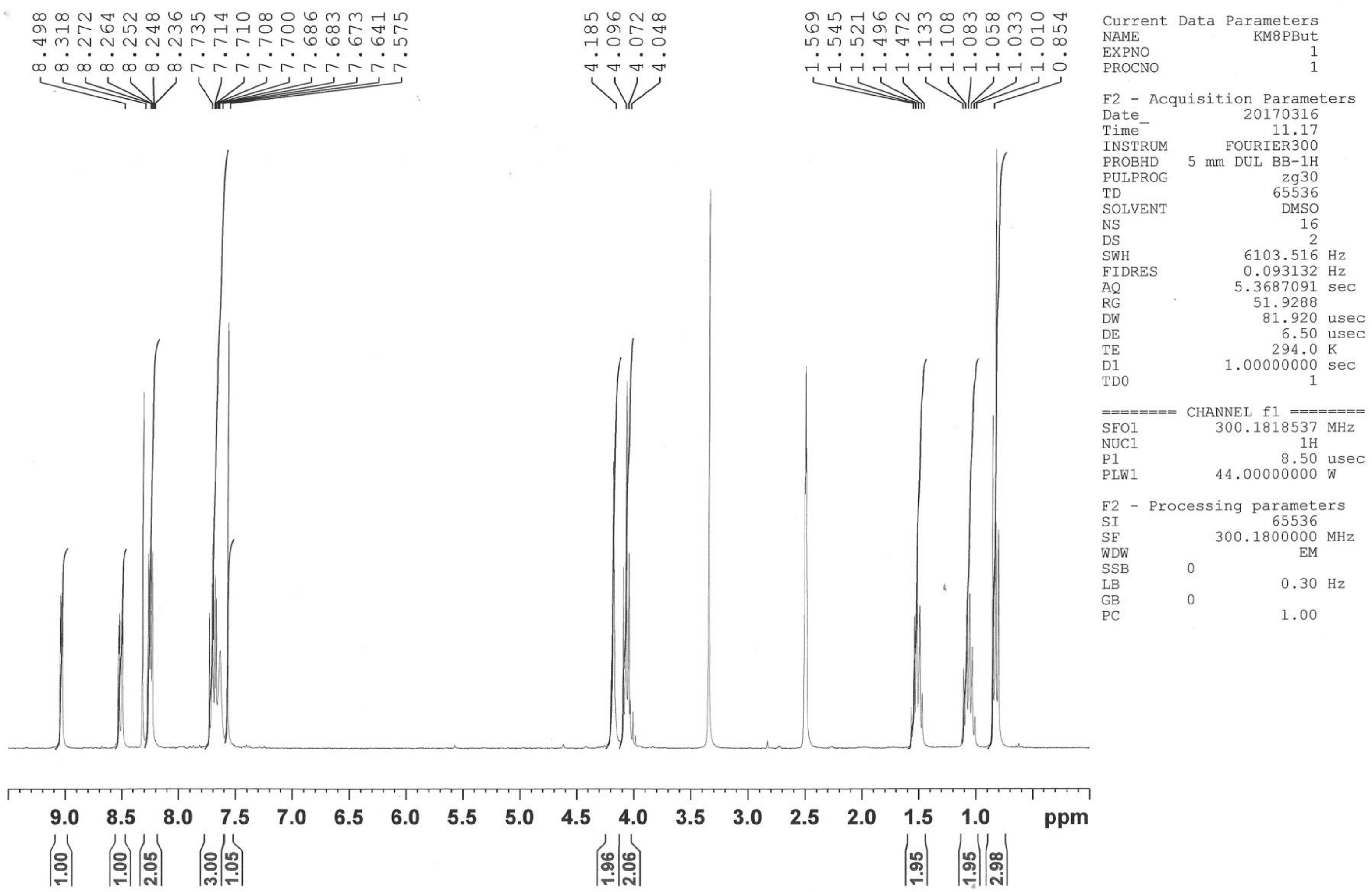

Figure S27 . ^{1}H NMR spectra of 8-*N*-{[1-(1-butyl)-1*H*-1,2,3-triazol-4-yl]methyl}quinolinesulfonamide (**9d**)

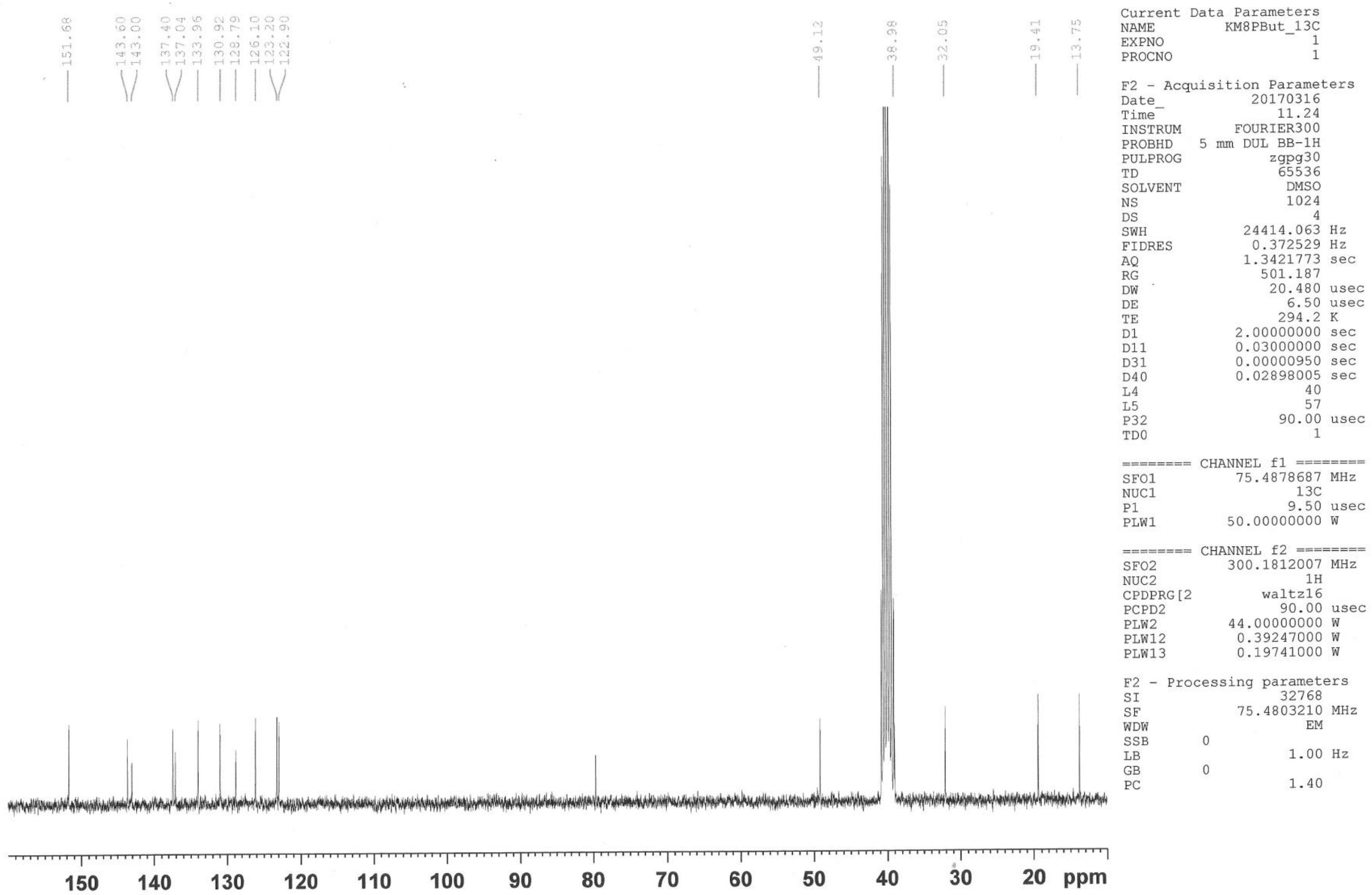

Figure S28. ^{13}C NMR spectra of 8-*N*-{[1-(1-butyl)-1*H*-1,2,3-triazol-4-yl]methyl}quinolinesulfonamide (**9d**)

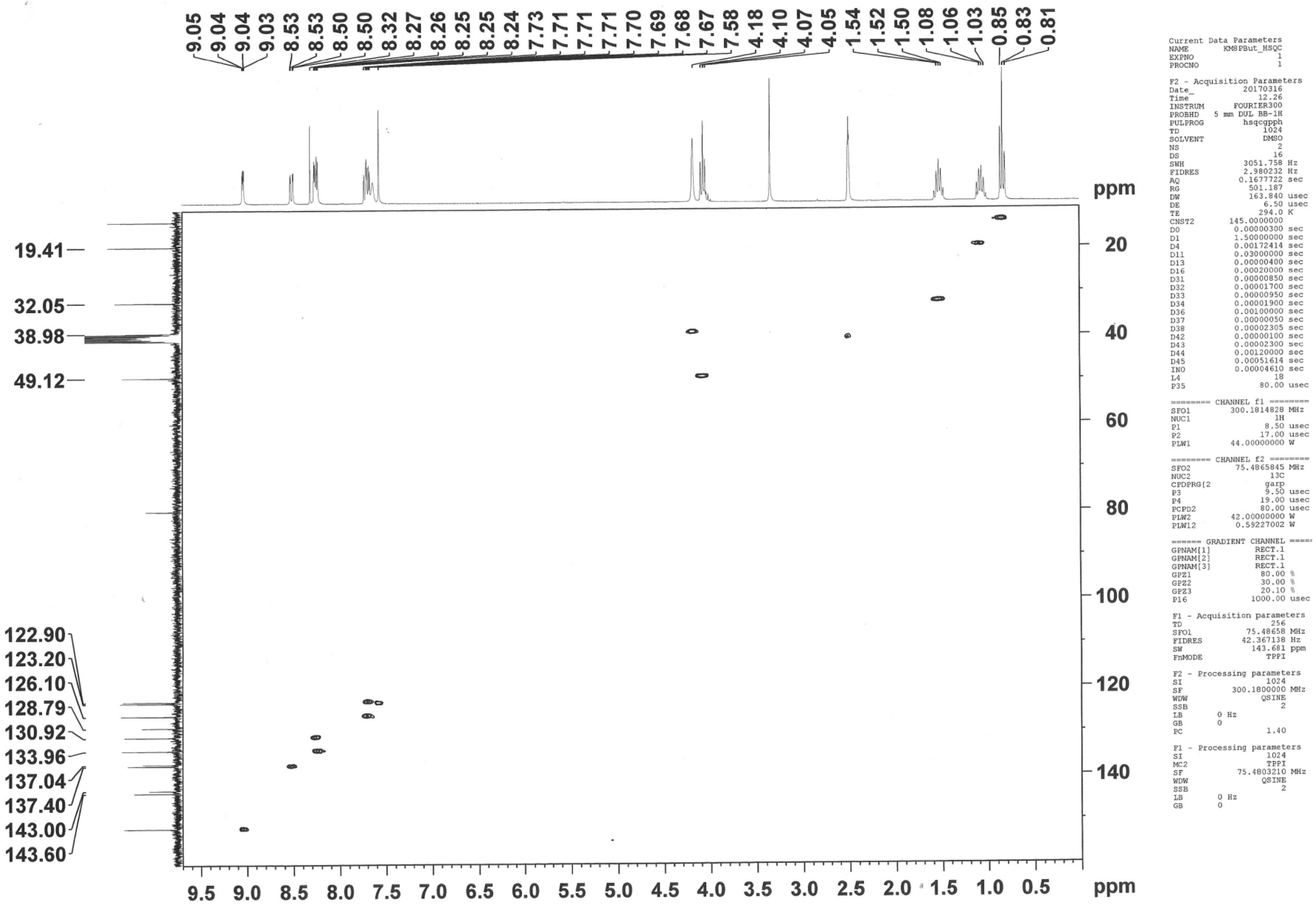

Figure S29. HSQC spectra of 8-*N*-{[1-(1-butyl)-1*H*-1,2,3-triazol-4-yl]methyl}quinolinesulfonamide (**9d**)

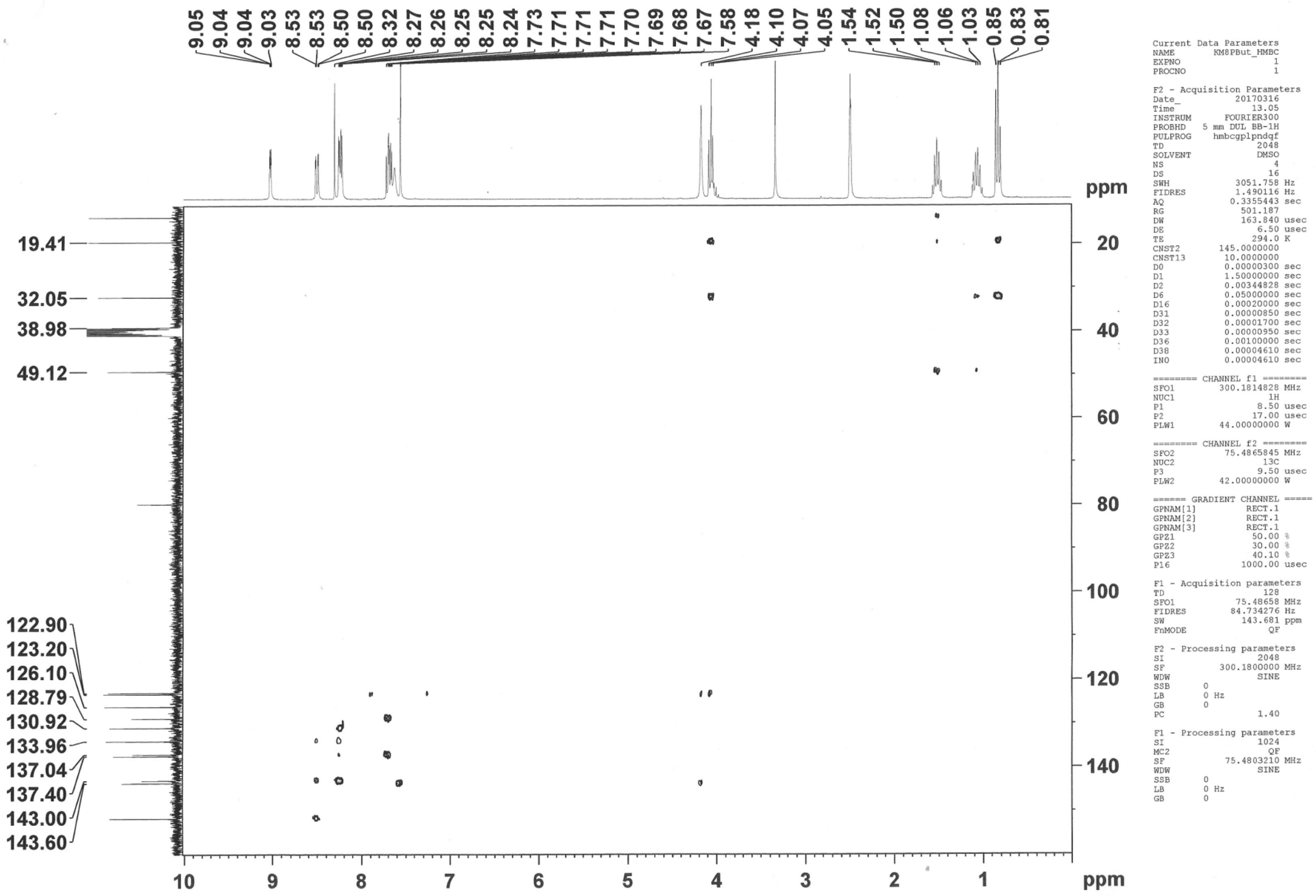

Figure S30. HMBC spectra of 8-*N*-{[1-(1-butyl)-1*H*-1,2,3-triazol-4-yl]methyl}quinolinesulfonamide (**9d**)

Compound Spectrum List Report

Analysis Info

Analysis Name D:\Data\KMMeBetulinaPositive.d
Method low_mass.m
Sample Name TM Low concentration
Comment
Operator KM
Instrument impact II 1825265.10082

Acquisition Parameter

Source Type	ESI	Ion Polarity	Positive	Set Nebulizer	0.3 Bar
Focus	Active	Set Capillary	4000 V	Set Dry Heater	240 °C
Scan Begin	100 m/z	Set End Plate Offset	-500 V	Set Dry Gas	4.0 l/min
Scan End	1000 m/z	Set Charging Voltage	2000 V	Set Divert Valve	Source
		Set Corona	0 nA	Set APCI Heater	0 °C

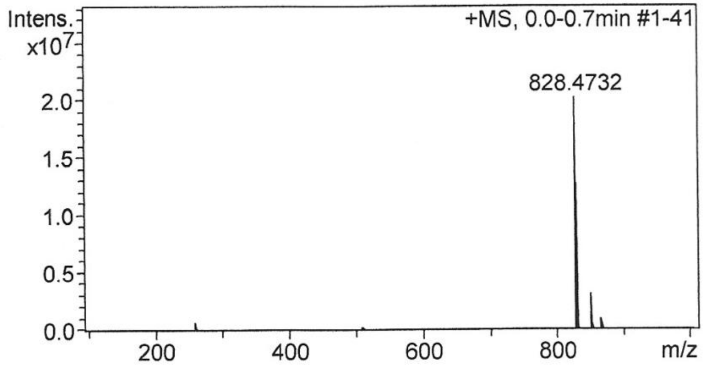

#	m/z	Res.	S/N	I	I %	FWHM
1	828.4732	36740	57187.3	20214546	100.0	0.0225
2	850.4540	54137	9022.8	3173013	15.7	0.0157
3	866.4274	43927	2836.8	980902	4.9	0.0197

Figure S31. HR MS spectra of 8-*N*-methyl-*N*-({1-[3β, 28-diacetoxylup-20(29)-en-30-yl]-1*H*-1,2,3-triazol-4-yl}methyl)-quinolinesulfonamide (**9e**)

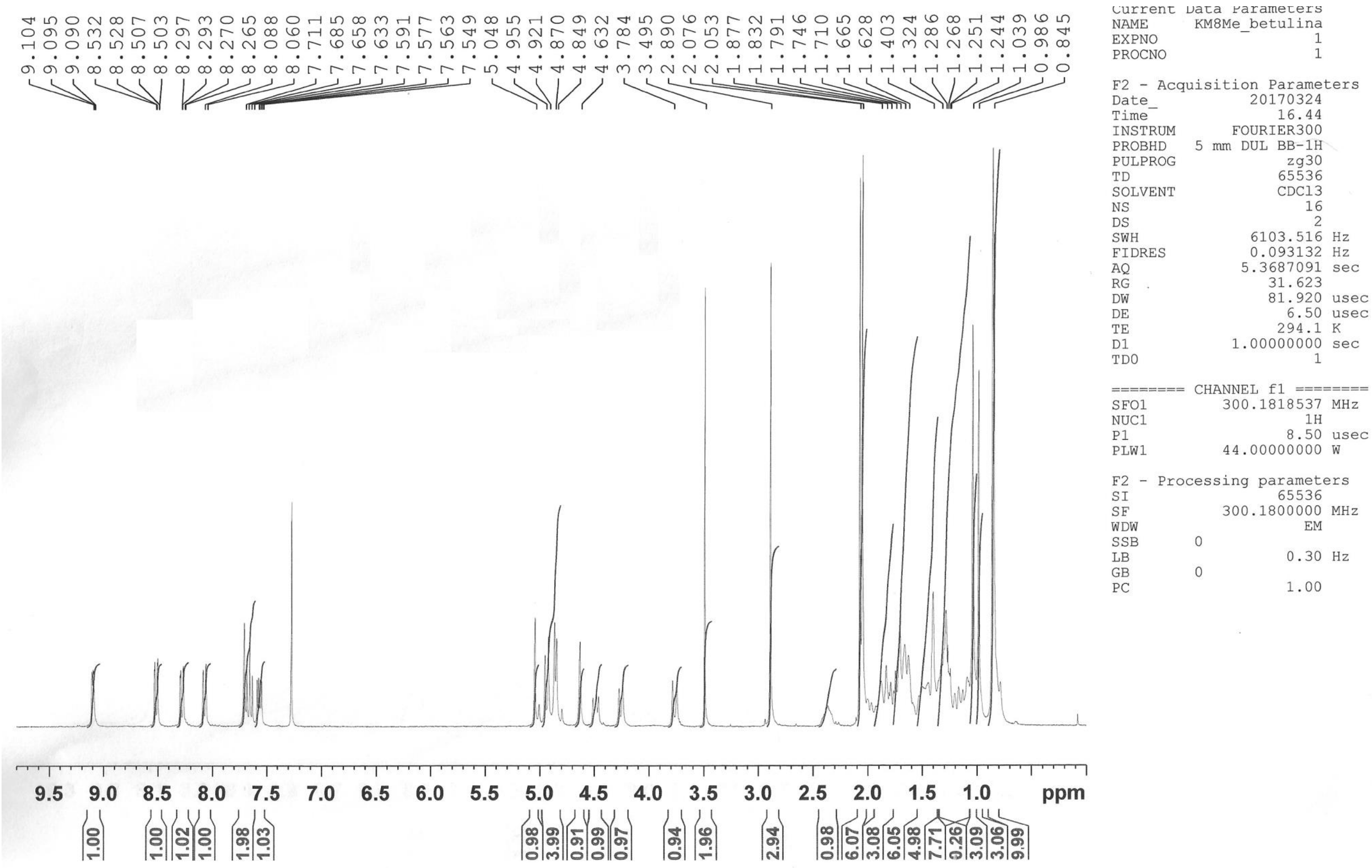

Figure S32 . ^{1}H NMR spectra of 8-*N*-methyl-*N*-({1-[3β, 28-diacetoxylup-20(29)-en-30-yl]-1*H*-1,2,3-triazol-4-yl}methyl)-quinolinesulfonamide (**9e**)

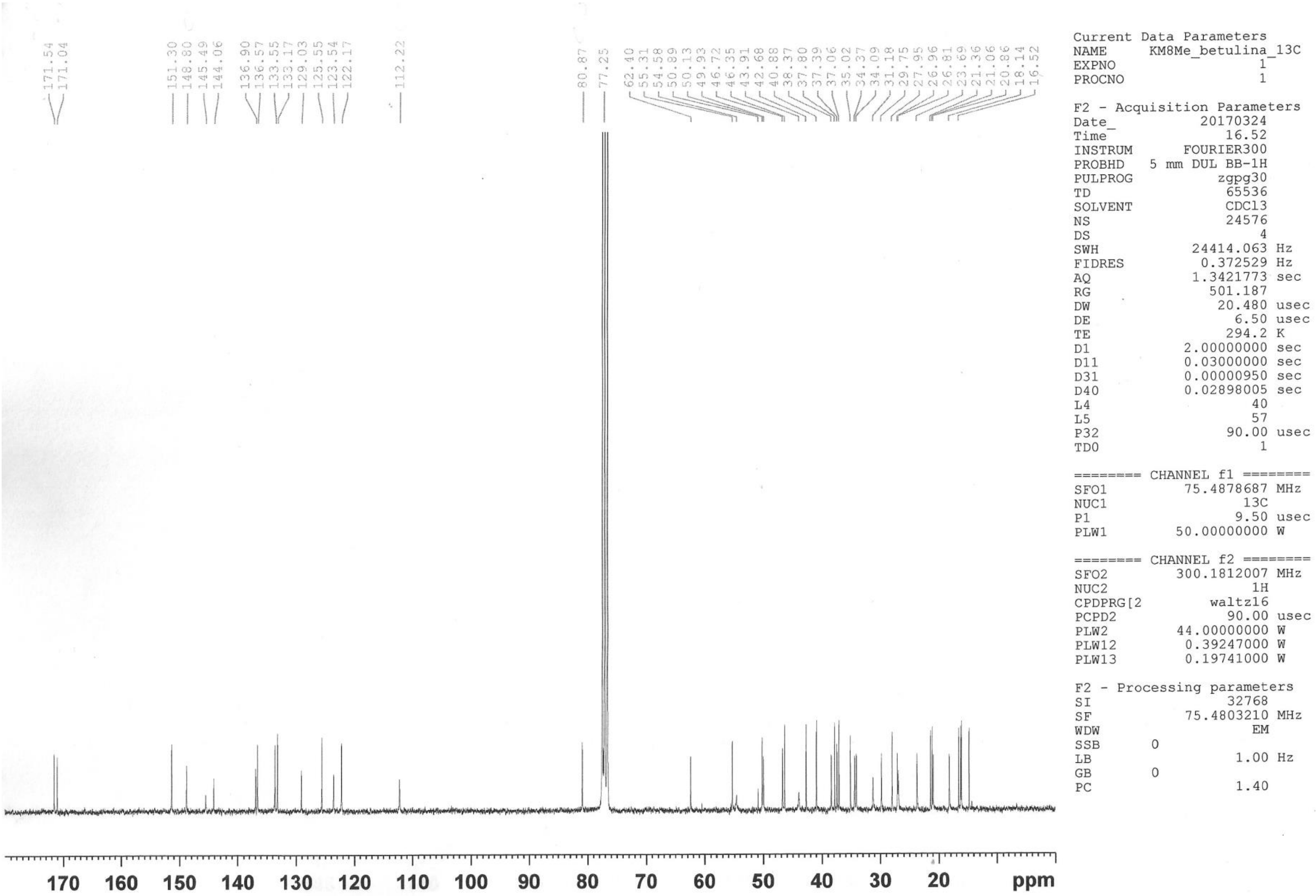

Figure S33 . ^{13}C NMR spectra of 8-*N*-methyl-*N*-({1-[3β, 28-diacetoxylup-20(29)-en-30-yl]-1*H*-1,2,3-triazol-4-yl}methyl)-quinolinesulfonamide (**9e**)

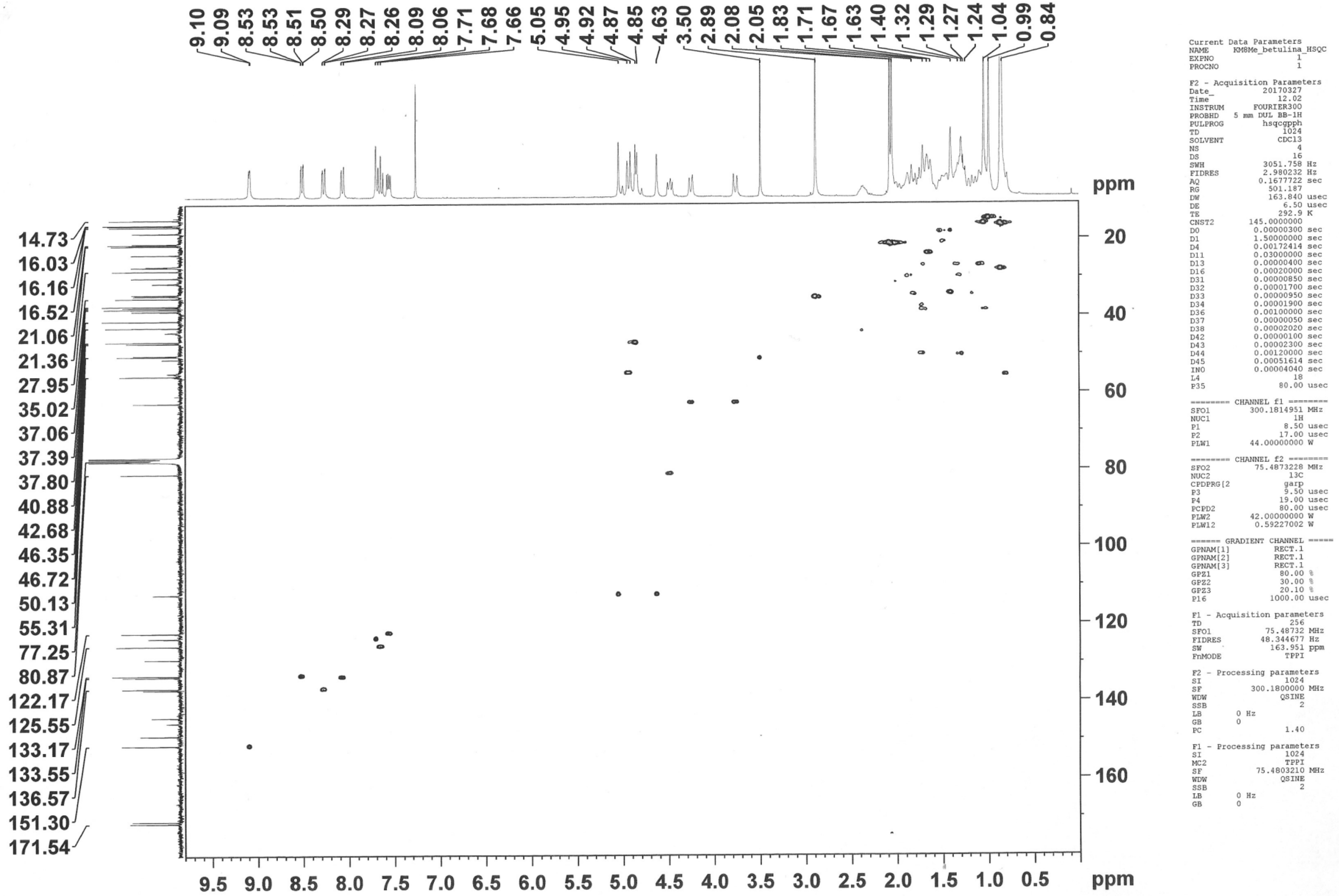

Figure S34 . HSQC spectra of 8-*N*-methyl-*N*-({1-[3β, 28-diacetoxylup-20(29)-en-30-yl]-1*H*-1,2,3-triazol-4-yl}methyl)-quinolinesulfonamide (**9e**)

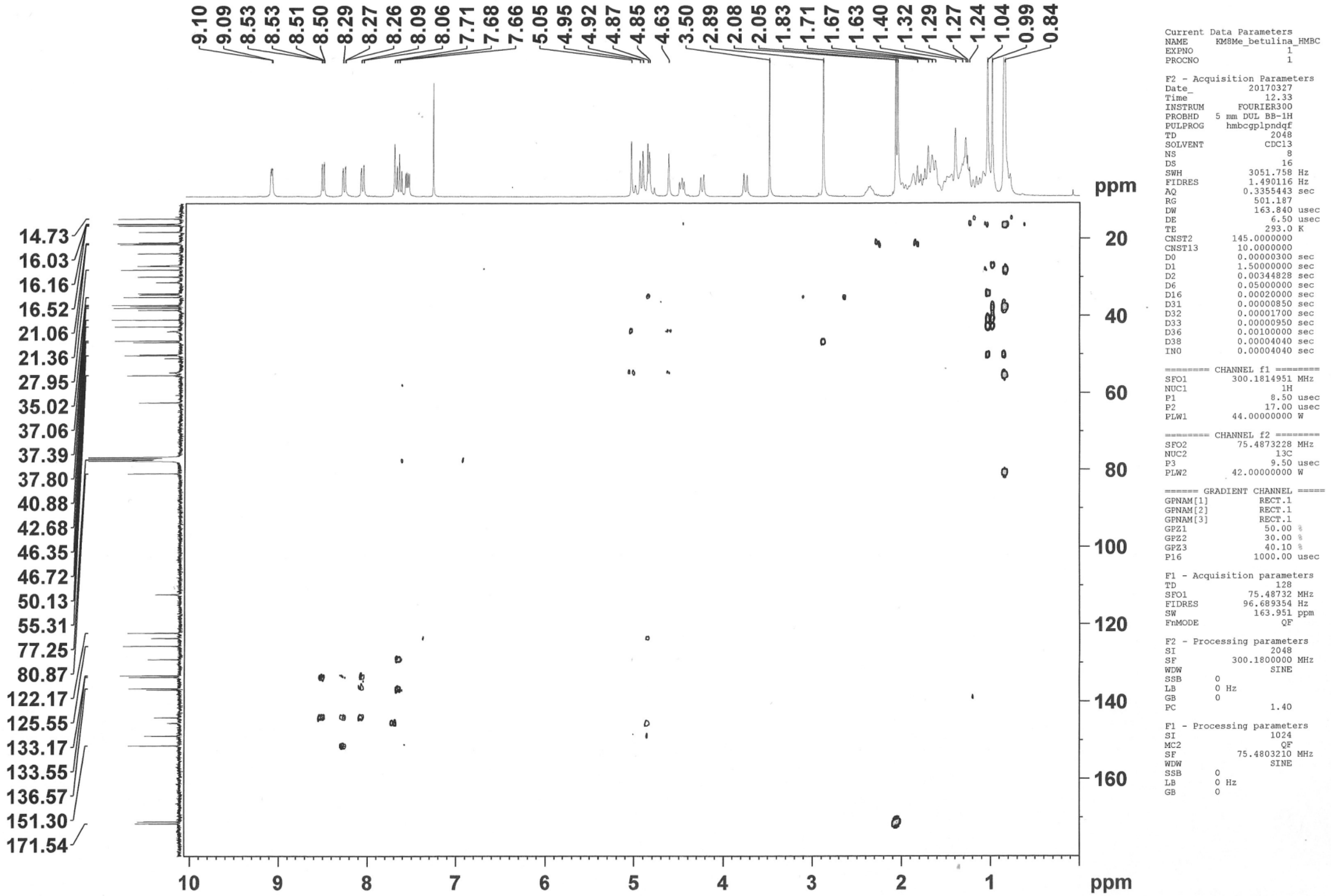

Figure S35 . HMBC spectra of 8-*N*-methyl-*N*-({1-[3β, 28-diacetoxylup-20(29)-en-30-yl]-1*H*-1,2,3-triazol-4-yl}methyl)-quinolinesulfonamide (**9e**)

Chromatogram and Results

Injection Details

Injection Name:	**9a**	*Run Time (min):*	**5.00**
Vial Number:	**RA1**	*Injection Volume:*	**5.00**
Injection Type:	**Unknown**	*Channel:*	**UV_VIS_1**
Calibration Level:		*Wavelength:*	**277.0**
Instrument Method:	**ACN 80 HCOOH**	*Bandwidth:*	**2**
Processing Method:	**LC MS**	*Dilution Factor:*	**1.0000**
Injection Date/Time:	**15/Feb/23 15:05**	*Sample Weight:*	**1.0000**

Chromatogram

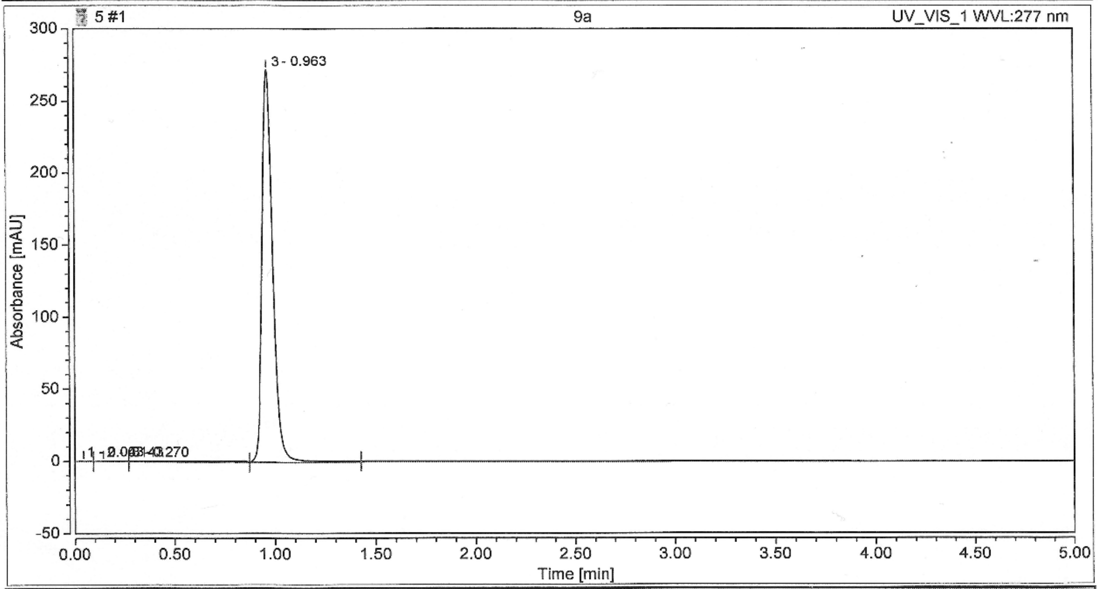

Integration Results

No.	Peak Name	Retention Time min	Area mAU*min	Height mAU	Relative Area %	Relative Height %	Amount n.a.
1		0.043	0.012	0.186	0.06	0.07	n.a.
2		0.143	0.047	0.242	0.25	0.09	n.a.
3		0.963	17.259	272.469	99.69	99.85	n.a.
**Total:**			**17.318**	**272.897**	**100.00**	**100.00**	

Figure S36. HPLC analysis of 8-*N*-{[1-(7-chloroquinolin-4-yl)-1*H*-1,2,3-triazol-4-yl]methyl}quinolinesulfonamide (**9a**)

Chromatogram and Results

Injection Details

Injection Name:	**9b**	*Run Time (min):*	**5.00**
Vial Number:	**RA2**	*Injection Volume:*	**5.00**
Injection Type:	**Unknown**	*Channel:*	**UV_VIS_1**
Calibration Level:		*Wavelength:*	**277.0**
Instrument Method:	**ACN 80 HCOOH**	*Bandwidth:*	**2**
Processing Method:	**LC MS**	*Dilution Factor:*	**1.0000**
Injection Date/Time:	**15/Feb/23 14:36**	*Sample Weight:*	**1.0000**

Chromatogram

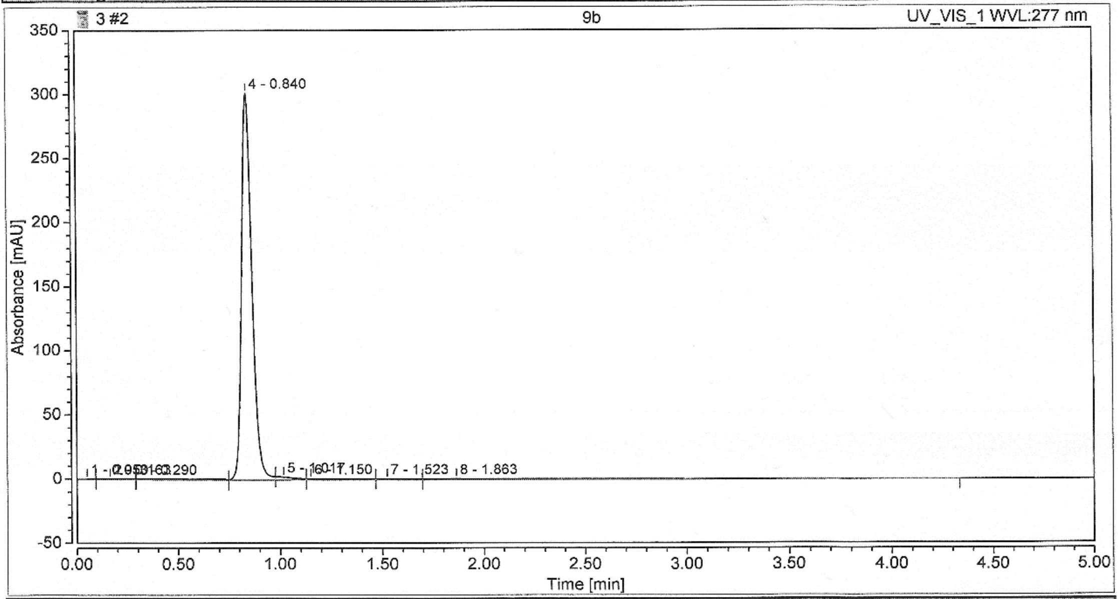

Integration Results

No.	Peak Name	Retention Time min	Area mAU*min	Height mAU	Relative Area %	Relative Height %	Amount n.a.
1		0.050	0.012	0.186	0.06	0.06	n.a.
2		0.163	0.056	0.275	0.29	0.09	n.a.
3		0.290	0.261	0.376	1.33	0.12	n.a.
4		0.840	17.635	301.871	90.15	98.19	n.a.
5		1.017	0.253	2.218	1.29	0.72	n.a.
6		1.150	0.282	0.965	1.44	0.31	n.a.
7		1.523	0.170	0.832	0.87	0.27	n.a.
8		1.863	0.892	0.724	4.56	0.24	n.a.
**Total:**			**19.562**	**307.447**	**100.00**	**100.00**	

Figure S37. diethyl 2-{4-[methyl-(8-sulfamoylquinolyl)-1*H*-1,2,3-triazol-1-yl]}ethylphosphonate (**9b**)

Chromatogram and Results

Injection Details

Injection Name:	**9c**	*Run Time (min):*	**5.00**
Vial Number:	**RA3**	*Injection Volume:*	**5.00**
Injection Type:	**Unknown**	*Channel:*	**UV_VIS_1**
Calibration Level:		*Wavelength:*	**277.0**
Instrument Method:	**ACN 80 HCOOH**	*Bandwidth:*	**2**
Processing Method:	**LC MS**	*Dilution Factor:*	**1.0000**
Injection Date/Time:	**15/Feb/23 14:42**	*Sample Weight:*	**1.0000**

Chromatogram

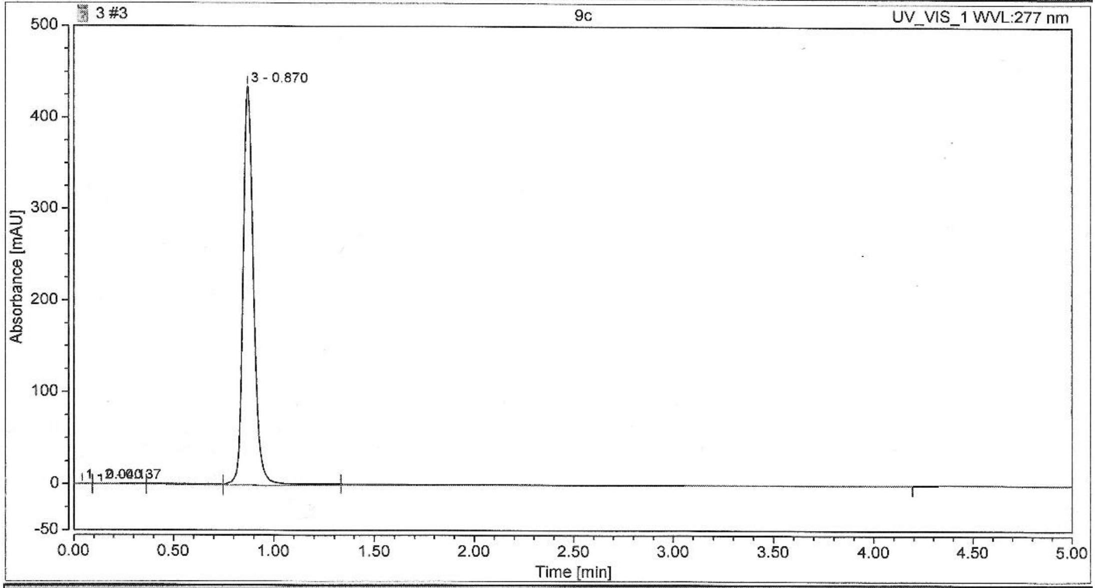

Integration Results

No.	Peak Name	Retention Time min	Area mAU*min	Height mAU	Relative Area %	Relative Height %	Amount n.a.
1		0.040	0.013	0.193	0.05	0.04	n.a.
2		0.137	0.084	0.243	0.30	0.05	n.a.
3		0.870	0.229	0.841	99.65	99.40	n.a.
**Total:**			**25.842**	**435.180**	**100.00**	**100.00**	

Figure S38. ethyl 3-{[4-(8-sulfamoylquinolyl)methyl]-1*H*-1,2,3-triazol-1-yl}propanoate (**9c**)

Chromatogram and Results

Injection Details

Injection Name:	9d	*Run Time (min):*	5.00
Vial Number:	RA4	*Injection Volume:*	5.00
Injection Type:	Unknown	*Channel:*	UV_VIS_1
Calibration Level:		*Wavelength:*	277.0
Instrument Method:	ACN 80 HCOOH	*Bandwidth:*	2
Processing Method:	LC MS	*Dilution Factor:*	1.0000
Injection Date/Time:	15/Feb/23 14:48	*Sample Weight:*	1.0000

Chromatogram

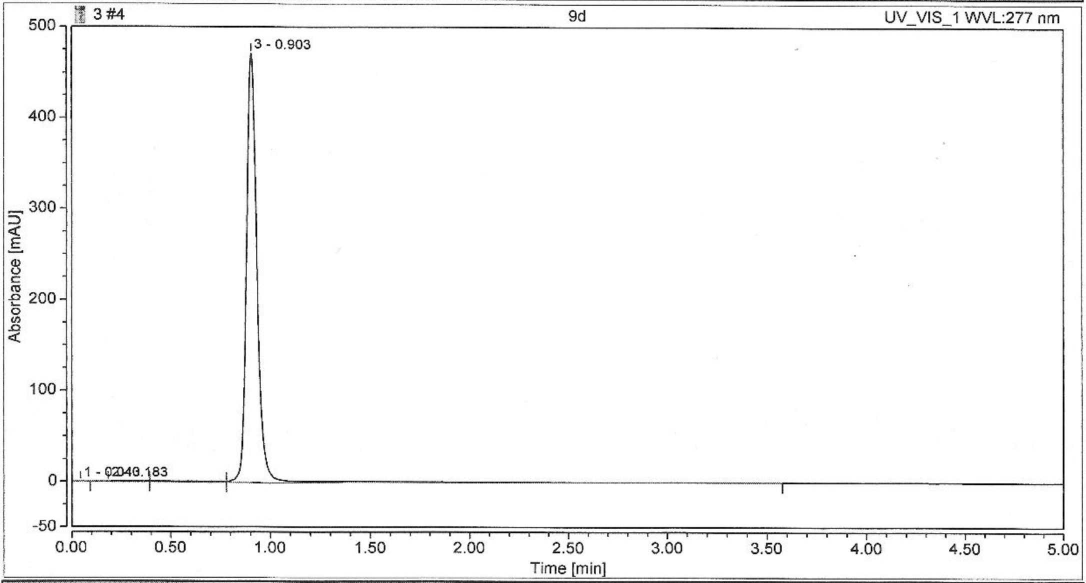

Integration Results

No.	Peak Name	Retention Time min	Area mAU*min	Height mAU	Relative Area %	Relative Height %	Amount n.a.
1		0.043	0.007	0.131	0.02	0.03	n.a.
2		0.183	0.087	0.263	0.30	0.05	n.a.
3		0.903	28.102	471.386	99.68	99.92	n.a.
**Total:**			**28.196**	**471.780**	**100.00**	**100.00**	

Figure S39. 8-*N*-{[1-(1-butyl)-1*H*-1,2,3-triazol-4-yl]methyl}quinolinesulfonamide (9d)

Chromatogram and Results

Injection Details			
Injection Name:	**9e**	*Run Time (min):*	**10.00**
Vial Number:	**RA5**	*Injection Volume:*	**5.00**
Injection Type:	**Unknown**	*Channel:*	**UV_VIS_1**
Calibration Level:		*Wavelength:*	**277.0**
Instrument Method:	**ACN 80 FA 20 10 min**	*Bandwidth:*	**2**
Processing Method:	**LC MS**	*Dilution Factor:*	**1.0000**
Injection Date/Time:	**15/Feb/23 14:54**	*Sample Weight:*	**1.0000**

Chromatogram

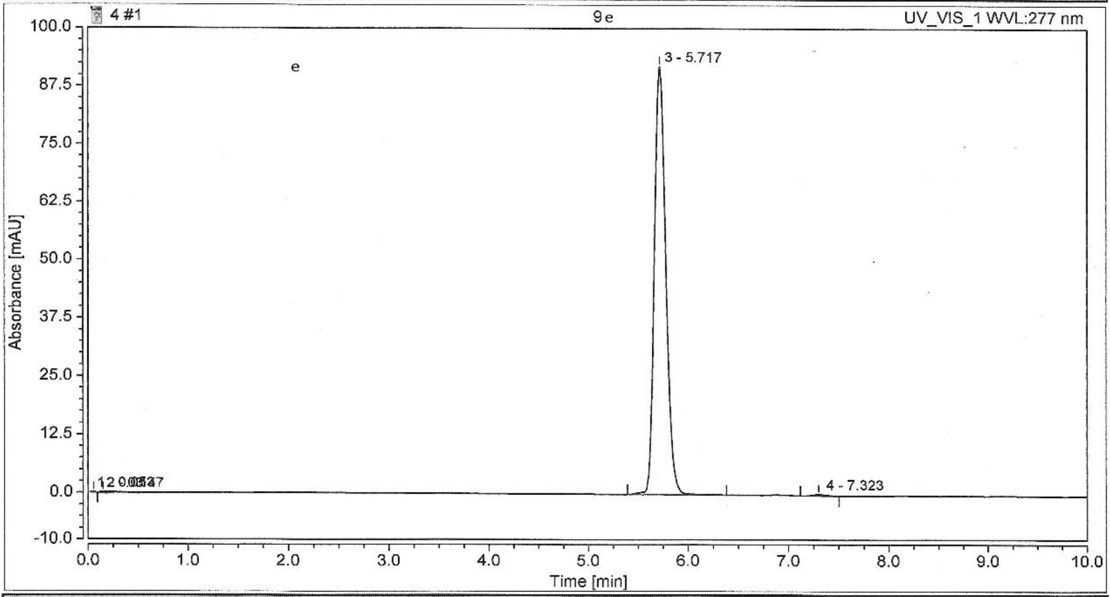

Integration Results

No.	Peak Name	Retention Time min	Area mAU*min	Height mAU	Relative Area %	Relative Height %	Amount n.a.
1		0.053	0.006	0.122	0.04	0.08	n.a.
2		0.147	0.092	0.221	0.54	0.14	n.a.
3		5.717	12.133	92.096	97.96	99.46	n.a.
4		7.323	0.248	0.486	1.46	0.32	n.a.
**Total:**			**12.479**	**92.925**	**100.00**	**100.00**	

Figure S40. 8-*N*-methyl-*N*-({1-[3β, 28-diacetoxylup-20(29)-en-30-yl]-1*H*-1,2,3-triazol-4-yl}methyl)-quinolinesulfonamide (**9e**)
